# N4BP1 functions as a dimerization-dependent linear ubiquitin reader which regulates TNF signalling

**DOI:** 10.1038/s41420-024-01913-8

**Published:** 2024-04-20

**Authors:** Katarzyna W. Kliza, Wei Song, Irene Pinzuti, Simone Schaubeck, Simone Kunzelmann, David Kuntin, Arianna Fornili, Alessandro Pandini, Kay Hofmann, James A. Garnett, Benjamin Stieglitz, Koraljka Husnjak

**Affiliations:** 1 Institute of Biochemistry II, Goethe University School of Medicine, Frankfurt (Main), Germany; 2 School of Biological and Behavioural Sciences, Queen Mary University of London, London, UK; 3 Structural Biology Science Technology Platform, Francis Crick Institute, London, UK; 4 School of Physical and Chemical Sciences, Queen Mary University of London, London, UK; 5 Department of Computer Science, Brunel University London, Uxbridge, UK; 6 Institute for Genetics, University of Cologne, Cologne, Germany; 7 Centre for Host-Microbiome Interactions, Dental Institute, King’s College London, London, UK; 8 Present Address: Max Planck Institute of Molecular Physiology, Otto-Hahn-Straße 11, 44227 Dortmund, Germany; 9 Present Address: Department of Oncology, University of Oxford, Oxford, UK; 10 Present Address: Department of Biology, University of York, Wentworth Way, York, UK

**Keywords:** Solution-state NMR, NF-kappaB

## Abstract

Signalling through TNFR1 modulates proinflammatory gene transcription and programmed cell death, and its impairment causes autoimmune diseases and cancer. NEDD4-binding protein 1 (N4BP1) is a critical suppressor of proinflammatory cytokine production that acts as a regulator of innate immune signalling and inflammation. However, our current understanding about the molecular properties that enable N4BP1 to exert its suppressive potential remain limited. Here, we show that N4BP1 is a novel linear ubiquitin reader that negatively regulates NFκB signalling by its unique dimerization-dependent ubiquitin-binding module that we named LUBIN. Dimeric N4BP1 strategically positions two non-selective ubiquitin-binding domains to ensure preferential recognition of linear ubiquitin. Under proinflammatory conditions, N4BP1 is recruited to the nascent TNFR1 signalling complex, where it regulates duration of proinflammatory signalling in LUBIN-dependent manner. N4BP1 deficiency accelerates TNFα-induced cell death by increasing complex II assembly. Under proapoptotic conditions, caspase-8 mediates proteolytic processing of N4BP1, resulting in rapid degradation of N4BP1 by the 26 S proteasome, and acceleration of apoptosis. In summary, our findings demonstrate that N4BP1 dimerization creates a novel type of ubiquitin reader that selectively recognises linear ubiquitin which enables the timely and coordinated regulation of TNFR1-mediated inflammation and cell death.

## Introduction

TNFα is a potent, pleiotropic cytokine, regulating inflammation, immunity and programmed cell death [1]. Activation of TNFR1 induces the assembly of the membrane-bound TNFR1 signalling complex (TNFR1-SC) composed of adaptor proteins (i.e., TRADD), kinase RIP1 and E3 ligases, including LUBAC [2]. TNFR1 signalling highly depends on numerous posttranslational modifications, including linear ubiquitination [2, 3]. The E3 ligase complex LUBAC stabilizes TNFR1-SC by assembling linear ubiquitin (Ub) chains on several complex components (TNFR1, RIP1, NEMO). These modifications are recognized by a subset of linear Ub-binding domain (UBD)-containing (LUBID) proteins that further regulate downstream signalling, including the UBAN (Ub binding in ABIN and NEMO) domain in NFκB signalling essential modulator (NEMO) [3–7]. Together with K11-, K48- and K63-linked Ub chains [6, 8], these Ub linkages provide a platform for the recruitment of the kinase complex IKK to initiate NFκB pathway for pro-survival gene induction [2, 9].

Prolonged activation of TNFR1 signalling can also trigger the assembly of the cytosolic complex II, composed of TRADD, FADD, RIP1, c-FLIP and procaspase-8, subsequently leading to caspase-8 (CASP8) activation [10]. Among others, apoptosis progression requires removal of M1 linkages from FADD and linear Ub-modified substrates within TNFR1-SC [3, 11]. In line with that, LUBAC deficiencies promote complex II assembly and induce aberrant TNFα-mediated endothelial cell death [12]. N4BP1 was initially described as a substrate of E3 ligase NEDD4 [13] and shown to inhibit ubiquitination and proteasomal degradation of ITCH E3 ligase substrates [14]. N4BP1 was also identified, but not further studied, as a Ub binder in a protein array and Ub-interactor affinity enrichment-MS (UbIA-MS) screens [15, 16]. N4BP1 was previously identified as one of the genes negatively regulating basal NFκB activity [17] and shown to inhibit both canonical and noncanonical NFκB in neuroblastoma [18]. Furthermore, Gitlin et al. identified N4BP1 as a suppressor of cytokine production negatively regulated by CASP8 [19], whereas Shi et al. describe that N4BP1suppresses TLR-dependent activation of NFκB by binding and inhibiting NEMO [20].

We now show that N4BP1 acts as a novel linear ubiquitin reader that negatively regulates NFκB signalling by its unique dimerization-dependent ubiquitin-binding module that we named “Linear Ub-Interacting Domain in N4BP1” (LUBIN).

## Results

### N4BP1 selectively interacts with linear ubiquitin chains

**Fig. 1:**
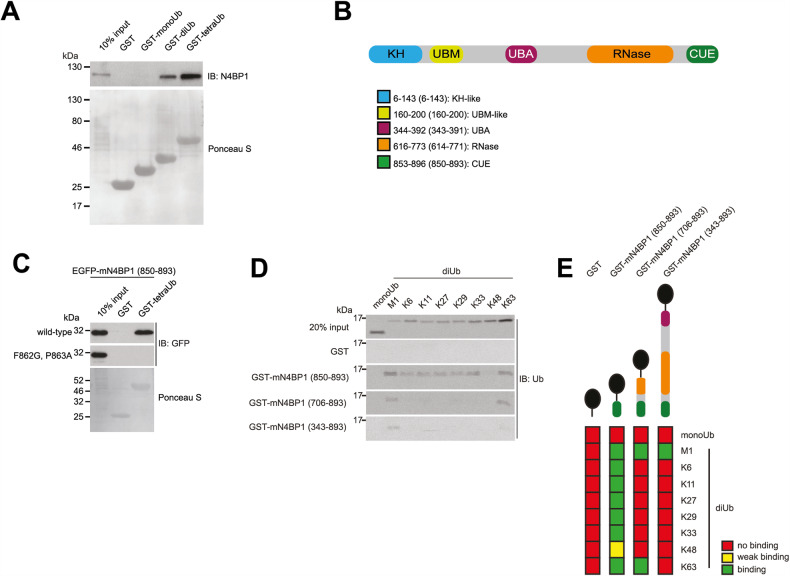
N4BP1 is a novel linear Ub-binding protein. **A** GST pull-down assay of endogenous mouse N4BP1 with GST fusions of mono-, di- and tetraUb. **B** Schematic representation of human N4BP1 with predicted domains. Domains: KH-like (K Homology), UBM-like (Ub-binding motif-like), divergent UBA (Ub-associated), NYN RNase and divergent CUE. Legend: the numbers outside and inside the brackets indicate amino acid residues of human and mouse N4BP1, respectively. **C** GST pull-down assay with GST fusions of mono- and tetraUb with N4BP1 CUE domain and predicted Ub binding-deficient (F862G, P863A) mutant transiently overexpressed in HEK293T cells as EGFP fusions. **D** GST pull-down assay with indicated recombinant N4BP1 fragments and chemically synthesized diUb chains linked by M1, K6, K11, K27, K29, K33, K48 and K63, as well as recombinant monoUb. **E** Schematic representation of the results is shown in (**D**). Red colour depicts the lack of binding, whereas yellow and green show weak and strong binding, respectively.

By using a Y2H assay, we identified a novel UBD in N4BP1 and demonstrated that endogenous N4BP1 binds linear di- and tetra-Ub but fails to interact with mono-Ub (Fig. 1A). The minimal UBD obtained by Y2H encompasses the divergent CUE domain of N4BP1 (Fig. 1B). Bioinformatic analysis identified two additional putative UBDs in N4BP1: non-functional UBM-like domain and divergent UBA domain (Fig. 1B, S1A, S1B), which binds to all Ub species (Fig. S1A). However, our Ub-binding analysis indicated that N4BP1’s divergent CUE domain is indispensable for specific recognition of linear Ub chains (Fig. S1A). Accordingly, mutation of the canonical Ub-binding motif, FP (F862G/P863A in mouse N4BP1), which is conserved among CUE domains (Fig. S1C) [21–23], abolished the binding between the isolated CUE domain and linear tetraUb (Fig. 1C).

Interestingly, when we probed Ub chain binding of different linkages with N4BP1 fragments of different sizes, we noticed that the CUE domain is required but not sufficient to generate linear Ub chain binding specificity (Fig. 1D, E). The isolated CUE domain displays robust interaction with all type of Ub chains when tested with synthetic di-Ubs [16, 24, 25]. Only binding to K48 linked di-Ub was less prominent. However, larger fragments which include domains adjacent to the CUE of N4BP1 clearly discriminate against isopeptide linked Ub chains and preferentially interact with M1-linked, linear Ub chains. We therefore conclude that N4BP1 involves additional structural elements outside the CUE domain which forms together a specificity module which mediate selective linear Ub chain binding and call this component LUBIN (Linear Ub-Interacting Domain Assembly in N4BP1).

### N4BP1 is a negative regulator of NFκB signalling

**Fig. 2:**
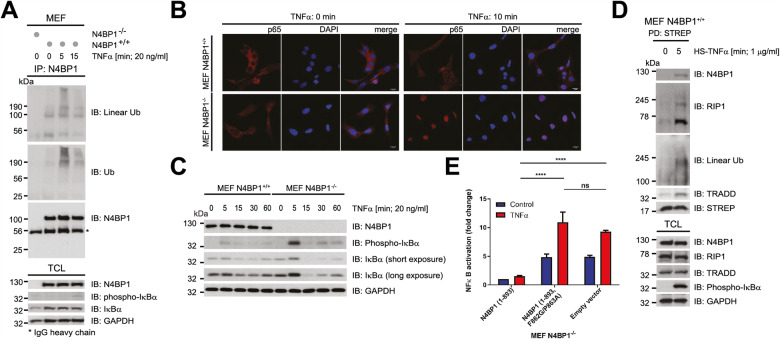
N4BP1 is a novel negative regulator of TNFR1 signalling. **A** Binding of M1-linked HMW Ub species to N4BP1. Immunoprecipitated endogenous N4BP1 and total cell lysates upon TNFα (20 ng/ml) treatment for indicated time periods were analysed by Western blotting with indicated antibodies. **B** Effect of N4BP1 on nuclear translocation of p65 upon TNFα stimulation. Sixteen hours post-starvation, N4BP1^+/+^ and N4BP1^−/−^ MEFs were treated with TNFα (20 ng/ml) for 10 min. Localization of p65 was detected by indirect immunofluorescence using anti-p65 antibody (1st and 4th column). Nucleoli were stained with DAPI (2nd and 5th column). **C** The effect of N4BP1 on activation of TNFα-mediated NFκB pathway. After 16 h of serum starvation, N4BP1^+/+^ and N4BP1^−/−^ MEFs were treated with TNFα (20 ng/ml) for indicated time periods. Total cell lysates were analysed by Western blotting with indicated antibodies. **D** Analysis of N4BP1 interaction with TNFR1-SC complex. After 16 h starvation, N4BP1^+/+^ MEFs were stimulated with recombinant HS-TNFα (1 μg/ml, 5 min) and TNFα-bound signalling complex was pulled down with Strep-tactin resins and subsequently resolved and analysed by Western blotting with indicated antibodies. **E** The effect of linear Ub binding-deficient N4BP1 on NFκB transcriptional activity. N4BP1^−/−^ MEFs stably expressing either empty vector, HA-N4BP1 (1-893) or HA-N4BP1 (1-893, F862G/P863A) were transiently transfected with pNFκB-Luc and pUT651 plasmids encoding luciferase and β-galactosidase, respectively. After 24 h, cells were starved for 16 h, followed by 6 h stimulation with TNFα (20 ng/ml). Lysates were subjected to luciferase and β-galactosidase assays. Three independent experimental replicates consisting of technical duplicates were performed. Results are shown as means and s.e.m. (*n* = 3). n.s. no statistically significant difference, *P < 0.05 and ****P < 0.0001, determined by two-way ANOVA test *post hoc* Sidak’s multiple comparisons test.

Basal cellular levels of linear Ub species are very low and their assembly is induced by stimuli, such as TNFα, IL-1β and poly (I:C) [5]. Identification of TNFα-induced linear Ub high molecular weight (HMW) species in immunoprecipitated endogenous N4BP1 samples, further confirmed the ability of N4BP1 to bind linear Ub chains (Fig. 2A, S2A), and prompted us to examine the role of N4BP1 in TNFα-induced NFκB signalling. Towards that aim, we utilized the N4BP1 knock-out (N4BP1^−/−^) and wild-type (N4BP1^+/+)^ mouse embryonic fibroblasts (MEFs) that express similar levels of various proteins involved in TNFR1 signalling pathway (Fig. S2B). We observed a strong increase of TNFα-induced NFκB transcription activity (Fig. S2C), the expression of TNFα target genes CXCL1 and IL-6 (Fig. S2D), and significantly increased nuclear translocation of NFκB subunit p65 (Fig. 2B, S2E) in N4BP1^−/−^ MEFs, contrary to N4BP1^+/+^ MEFs. Consistently, kinetics of the IκBα phosphorylation and degradation differ in N4BP1^−/−^ and N4BP1^+/+^ MEFs (Fig. 2C, S2F). Noteworthy, cytoplasmic N4BP1 (Fig. S2G–H) [26] specifically affects TNFα-induced NFκB pathway, as IκBα degradation kinetics induced by IL-1β stimulation remains unaltered in N4BP1^−/−^, compared to N4BP1^+/+^ MEFs (Fig. S2I). The effect is not an artefact of differential immortalization of N4BP1^−/−^ and N4BP1^+/+^ MEFs, since N4BP1 KO^CRISPR^ MEFs, in which N4BP1 was depleted in immortalized N4BP1^+/+^ MEFs by using CRISPR-Cas9 approach, behaved similarly to immortalized N4BP1^−/−^ MEFs (Fig. S2J). N4BP1 functions in close proximity to TNFR1, as it is recruited to the nascent TNFR1-SC within 5 min of TNFα stimulation (Fig. 2D). To determine the correlation between N4BP1 ability to bind linear Ub chains and its function in proinflammatory TNFR1 signalling, we monitored the activation of NFκB pathway upon TNFα stimulation in N4BP1^-/-^ MEFs reconstituted with either empty vector, HA-N4BP1 (1-893) or HA-N4BP1 (1-893, F862G/P863 A) (Fig. S3A). Contrary to N4BP1^+/+^, Ub binding-deficient N4BP1 mutant failed to restrict activation of NFκB pathway (Fig. 2E, S3B). The presence of N4BP1 stabilized TNFα-induced linear Ub HMW conjugates over time, implying the competition between N4BP1 and linear Ub-specific deubiquitinating enzymes (DUBs) for M1 linkages (Fig. S3C). We therefore conclude that N4BP1 is a component of TNFR1-SC that modulates proinflammatory TNFR1 signalling through its ability to specifically recognize linear Ub chains.

### N4BP1 is cleaved by CASP8 upon prolonged TNFα stimulation

**Fig. 3:**
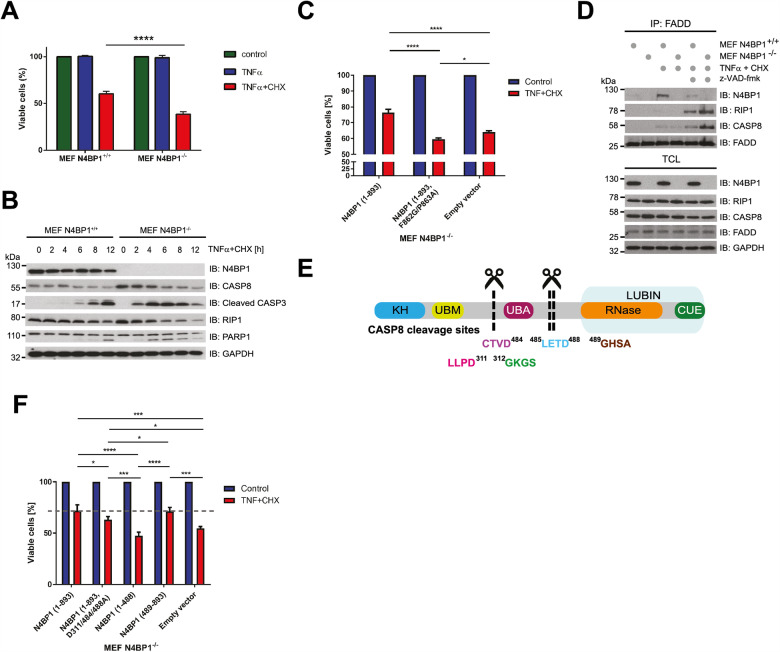
CASP8-mediated proteolytic processing of N4BP1 promotes TNFα-induced cell death. **A** Cell viability of N4BP1^+/+^ and N4BP1^−/−^ MEFs left untreated or exposed for 12 h to TNFα alone (10 ng/ml) or TNFα combined with CHX (10 ng/ml and 0.5 μg/ml, respectively). Cell survival was determined by crystal violet staining. Results are shown as means and s.e.m. (*n* = 3). ****P < 0.0001, determined by two-way ANOVA, *post hoc* Sidak’s multiple comparisons test. **B** The effect of N4BP1 on apoptotic cell death. N4BP1^+/+^ and N4BP1^−/−^ MEFs were treated with TNFα (10 ng/ml) and CHX (0.5 μg/ml) for indicated time periods. The proteolytic processing of apoptotic markers was measured by monitoring the appearance of a cleaved form of CASP3, the disappearance of the full-length CASP8 and RIP1, and both cleaved and uncleaved forms of PARP1. **C** Cell viability of N4BP1^−/−^ MEFs reconstituted with empty vector, N4BP1 (1-893) or N4BP1 (1-893, F862G/P863A) under apoptotic conditions. Cells were left untreated or exposed for 10 h to TNFα and CHX (10 ng/ml and 0.5 μg/ml, respectively). Cell viability was determined by the CellTiter-Glo Luminescent assay. Three independent experimental replicates consisting of technical triplicates were performed. Results are shown as means and s.e.m. (*n* = 3). n.s., no statistically significant difference, *P < 0.05, ****P < 0.0001, determined by two-way ANOVA, *post hoc* Sidak’s multiple comparisons test. **D** Effect of N4BP1 on proapoptotic complex II formation. N4BP1^+/+^ and N4BP1^−/−^ MEFs were left untreated or exposed to TNFα and CHX (10 ng/ml and 0.5 μg/ml, respectively) without or with z-VAD-fmk (20 μM) for 90 min. Immunoprecipitated endogenous FADD and total cell lysates were analysed by Western blotting. **E** Schematic representation of N4BP1 protein with indicated CASP8 cleavage sites. **F** The effect of N4BP1 and its cleavage fragments on cell viability. N4BP1^−/−^ MEFs reconstituted with empty vector, HA-N4BP1 (1-893), HA-N4BP1 (1-893, D311/484/488 A), HA-N4BP1 (1-488) or HA-N4BP1 (489–893) were either exposed for 10 h to TNFα (10 ng/ml) and CHX (0.5 μg/ml) or left untreated. Cell viability was determined by the CellTiter-Glo Luminescent assay. Three independent experimental replicates consisting of technical duplicates were performed. Results are shown as means and s.e.m. (*n* = 3). n.s. no statistically significant difference, ****P < 0.0001, determined by two-way ANOVA, followed by *post hoc* Tukey’s multiple comparisons test.

The presence of M1 linkages within TNFR1-SC preserves the architecture of the complex and prevents assembly of the complex II, which is a prerequisite for apoptotic cell death [2]. As such, we next examined the effect of N4BP1 on TNFα-mediated cell death. N4BP1-deficient MEFs were significantly sensitized to apoptosis induced by the coadministration of TNFα and CHX (Fig. 3A, B), a combined treatment commonly used to monitor TNFα -dependent cell death over time [27]. The suppressive effect of N4BP1 on apoptosis depends on a functional LUBIN, as N4BP1^−/−^ MEFs reconstituted with HA-N4BP1 (1-893, F862G/P863A) are significantly more susceptible to cell death than N4BP1^−/−^ MEFs stably expressing HA-N4BP1 (1-893) (Fig. 3C). Accordingly, N4BP1 deficiency enhanced the assembly of RIP1 and proximal components of the complex II (FADD and CASP8) under proapoptotic conditions (Fig. 3D, lanes 5 and 6). Moreover, N4BP1 was found in complex II upon cell death-inducing treatments (Fig. 3D, lanes 3 and 5). FADD is modified by LUBAC and deubiquitinated upon apoptosis induction [11]. Hence, the observed recruitment of N4BP1 to complex II could be explained by the recognition of linear Ub-modified FADD by N4BP1 LUBIN, which presumably sequesters FADD in TNFR1-SC, thereby slowing down the complex II formation. Interestingly, we observed that prolonged TNFα stimulation results in the proteolytic cleavage of N4BP1 (Fig. S4A). Important regulators of TNFR1 signalling, such as RIP1, CYLD and catalytic LUBAC subunit HOIP, are targets of CASP8-mediated cleavage [11, 28, 29]. Based on the computational analysis [30], CASP8 was the most promising candidate to cleave N4BP1. Indeed, N4BP1 interacts and is processed by CASP8 both in vivo and in vitro (Fig. S4B–E), with cleavage pattern indicating multiple cleavage sites within N4BP1 (Fig. S4F–G). Mass spectrometry (MS)-based analysis of TNFα- and CHX-treated, immunoprecipitated N4BP1, identified two N4BP1 peptides ^477^QNSSCTVDLETD^488^ and ^297^QFSLENVPEGELLPD^311^ that are most likely a result of the CASP8 proteolytic activity (Fig. S5A–B). The mutational analysis confirmed N4BP1 residues D311 and D488 as major CASP8 recognition sites and showed that D484 residue is also cleaved, likely due to its close proximity to D488 (Fig. 3E, S5C). Proteolytic processing of N4BP1 generates a N4BP1 (1-488) fragment, which preserves the ability to unspecifically bind Ub (Fig. S5D), and the N4BP1 (489–893) fragment, which retains ability to specifically interact with linear Ub chains (Fig. S5D). N4BP1^−/−^ MEFs reconstituted with full-length HA-N4BP1 (1-893), uncleavable N4BP1 (1-893, D311/484/488 A) or LUBIN-containing HA-N4BP1 (489–893) could suppress TNFα-mediated cell death, contrary to cells reconstituted with either empty vector or HA-N4BP1 (1-488) (Fig. 3F). The effect of LUBIN-containing N4BP1 fragment was striking, considering its very low expression level in N4BP1^−/−^ MEFs (Fig. S5E). Noteworthy, we observed that LUBIN-containing N4BP1 (489–893) fragment is unstable and undergoes proteasomal degradation (Fig. S5F) and we therefore conclude that CASP8-mediated proteolytic processing of N4BP1 removes antiapoptotic LUBIN-containing fragment, thereby facilitating apoptosis.

### An N4BP1 dimer functions as a unique linear ubiquitin reader

**Fig. 4:**
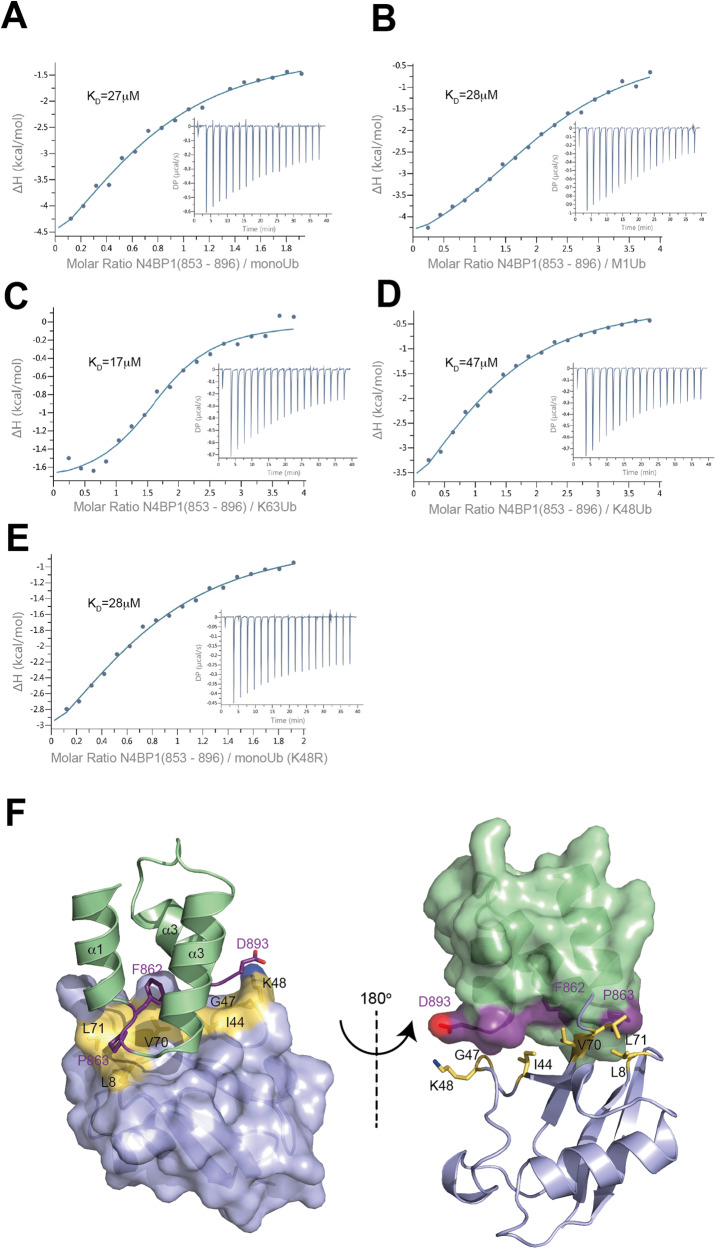
The isolated CUE domain of N4BP1 is a nonselective mono ubiquitin binding module. Isothermal titration calorimetry measurements demonstrate that N4BP1 (850–893) displays similar affinities for monoUb (**A**), M1-diUb (**B**), K63-diUb (**C**), K48-diUb (**D**), monoUb (K48R) (**E**). **F** Structural model of the CUE domain of N4BP1 (green) in the complex with Ub (blue) in a cartoon and surface presentation. Residues of N4BP1 and Ub, which form the interface of the complex are shown in purple and yellow respectively.

**Fig. 5:**
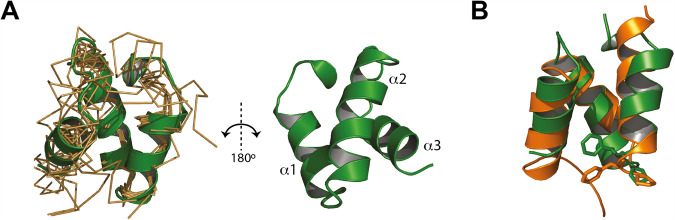
Solution structure of the N4BP1 CUE domain. **A** Ribbon diagram and backbone bundle presentation of conformers with the lowest residual target functions of residues of N4BP1 850–893. **B** Overlay of the CUE domains of N4BP1 (green) and GP78 (ornage). The canonical FP motif is indicated as ball-and-stick model.

We next set out to investigate the Ub-binding properties of N4BP1 in more detail with the aim to elucidate the molecular basis of LUBIN-mediated linear Ub chain binding specificity under in vitro conditions with purified, tag-free proteins. We first quantified the binding affinities for N4BP1 by isothermal titration calorimetry and determined K_D_ values for the isolated CUE domain (Table 1, Fig. 4A–D). Since the obtained equilibrium dissociation constants did not indicate a clear preferential interaction between N4BP1 and M1-linked Ub chains, we aimed to further understand the Ub binding mode of N4BP1 in atomic detail and characterised the structure of the CUE domain and its Ub binding properties by NMR spectroscopy. We established a structural model for the N4BP1 CUE domain spanning residues 850–893 (Fig. 5A). The obtained structure folds into the canonical three-helical bundle, as observed for other CUE domains [31] (Fig. 5B).

**Table 1:** K_D_ values of different N4BP1 fragments with various ubiquitin linkages determined by ITC and SPR measurements.

ITC		K_D_ [μM]	ΔH [kcal/mol]	-TΔS [kcal/mol]	ΔG [kcal/mol]	*N*
N4BP1 (850–893)	monoUb	27.5 ± 5.6	−5.2	−1.03	−6.21	0.915
	M1-diUb	28.1 ± 2.7	−5.3	−0.86	−6.21	2.16
	K63-diUb	16.7 ± 8.2	−5.88	−3.15	−6.62	1.71
	K48-diUb	47.0 ± 5.6	−5.87	−0.42	−5.67	1.29
	monoUb K48A	no binding detected	-	-	-	-
	monoUb K48R	28.1 ± 9	−4.61	−1.58	−6.2	0.82

SPR		K_D_ [μM]				
N4BP1 (613-774)	monoUb	no binding detected				
	M1-diUb					
	K63-diUb					
	K48-diUb					
N4BP1 (613–893)	monoUb	84.93				
	M1-diUb	0.43				
	K63-diUb	10.85				
	K48-diUb	34.23

**Fig. 6:**
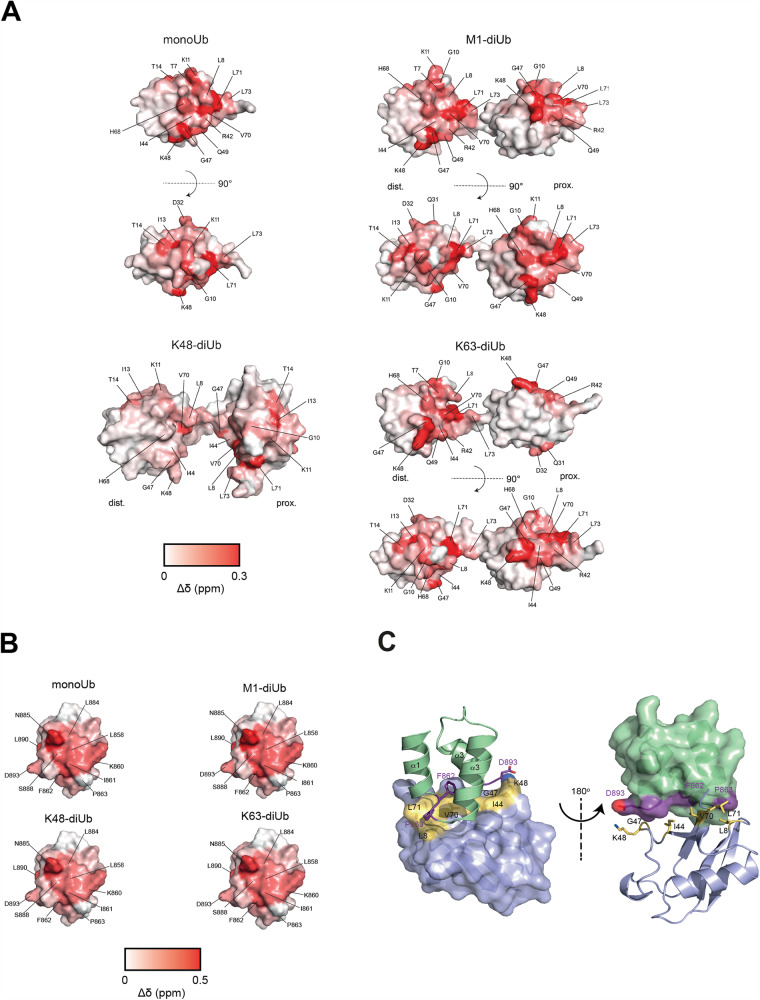
Interface analysis of N4BP1 CUE—Ubiquitin complexes by NMR chemical shift mapping. **A** Perturbed surface of different Ub linkages upon binding to N4BP1 (850–893). The chemical shift perturbations for each residue were mapped onto the surface of monoUb (PDB ID: 1UBQ), M1-diUb (PDB ID: 2W9N), K63-diUb (PDB ID: 3H7P) and K48-diUb (PDB ID: 1ZO6). Residues with strong chemical shift perturbations are indicated. Red gradient indicates the intensity of the observed chemical shift perturbations (Δδ). ^1^H-^15^N-HSQC signals, which were completely broadened, were set to a maximum value of 0.3 ppm (prox.: proximal Ub; dist.: distal Ub). **B** Perturbed surface of the CUE domain upon binding to the corresponding Ub linkage. Residues with strong chemical shift perturbations are indicated. Red gradient indicates the intensity of the observed chemical shift perturbations (Δδ). ^1^H-^15^N-HSQC signals, which were completely broadened, were set to a maximum value of 0.5 ppm. **C** Structural model of the CUE domain of N4BP1 (green) in the complex with Ub (blue) in a cartoon and surface presentation. Residues of N4BP1 and Ub, which form the interface of the complex are shown in purple and yellow respectively.

To reveal if and how the CUE domain forms distinctive interfaces with different Ub linkages, we performed ^1^H-^15^N HSQC NMR titration experiments with monoUb, K48-, K63- and M1-linked diUb (Fig. 6A, B, S6A, B). All interface areas between the CUE domain and Ub species are highly similar, confirming that the isolated CUE domain of N4BP1 recognizes Ub in a nonspecific manner. We found that it forms a small hydrophobic interface of 830 Å^2^ with Ub, which comprises the aliphatic portion of the C-terminal D893 of helix α3 and F862 and P863 residues located between helices α1 and α2 (Fig. 6C) and recognizes a nonpolar surface area of Ub, involving the hydrophobic patch around I44. Chemical shift mapping of monoUb, K63- or M1-linked diUb show marked chemical shift perturbation (CSP) values for K48 residue of Ub, which is absent in K48-linked diUb (Fig. S7A) and contributes to the interface by forming a contact with D893 of the CUE domain of N4BP1(Fig. 6C). Mutations D893A in N4BP1 or K48A in Ub, abolish complex formation, indicating that this polar interaction is essential for robust binding (Fig. S7B, Table 1). Interestingly, mutation K48R in Ub does not affect the affinity towards N4BP1 (Fig. S7C), which demonstrates that a positive charge at the position K48 plays a dominant role in the interaction with N4BP1, a finding that is in line with the reduced ability of the isolated CUE domain to co-precipitate K48-diUb (Fig. 1D, E). Here, the side chain of the K48 participates in the formation of the isopeptide bond, and therefore, only the distal Ub moiety of K48-linked Ub chains can fully engage with the CUE domain of N4BP1, causing a reduced binding affinity compared to other Ub linkages.

**Fig. 7:**
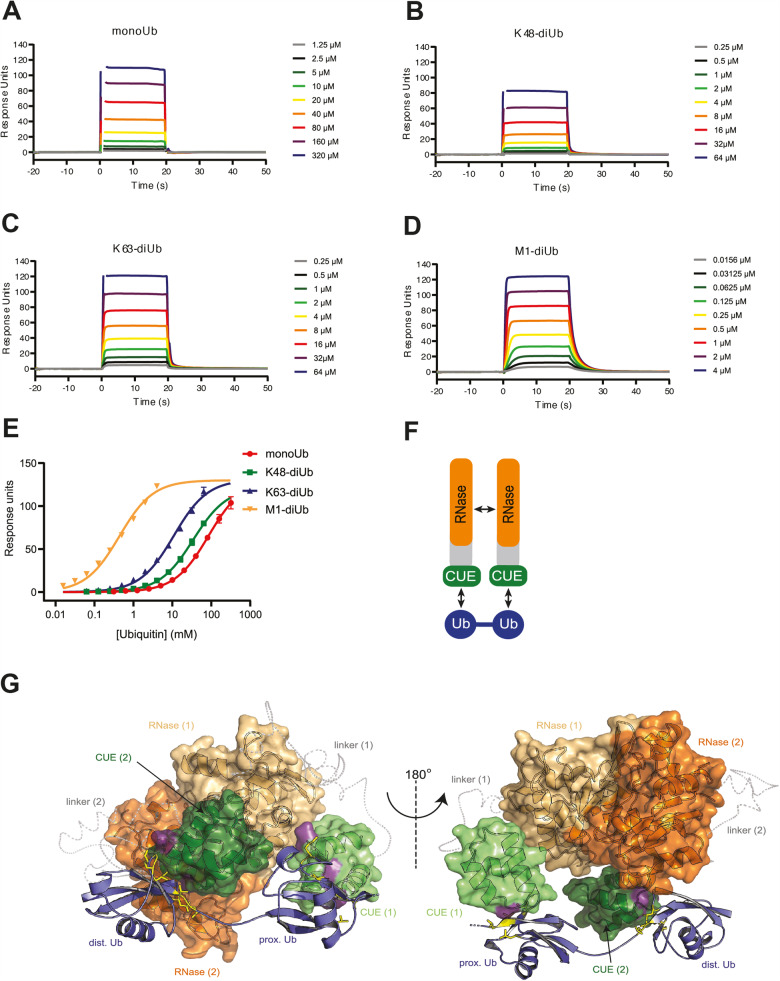
N4BP1 achieves linear ubiquitin binding through dimerization of its RNase and CUE domains. Quantitative analysis of Ub binding specificity of dimeric N4BP1 by surface plasmon resonance (SPR). Sensograms for N4BP1 (613–893) interaction with monoUb (**A**), K48-diUb (**B**), K63-diUb (**C**) and M1-diUb (**D**) at different concentrations. **E** Average binding responses of increasing concentrations of monoUb and K48-, K63- and M1-linked diUb were fitted to a saturation equilibrium binding model to obtain equilibrium dissociation constants. **F** Schematic representation of N4BP1 mediated linear Ub interaction. Dimerization of the RNase domains allows selective binding of M1-linked di-Ub via the CUE domains. **G** Structural model of the N4BP1 (613–893) dimer in complex with M1-linked diUb. A homology model of the RNase domain was generated using residues 135-339 of the X-ray structure of dimeric MCPIP1 (PDB ID: 5H9W) as a template. The RNase domain (orange) is interconnected to the NMR based solution structure of the CUE domain (green) via a flexible linker region (residues 775-849), which is shown as a C-α backbone trace (grey, dashed line). The dimeric arrangement of N4BP1 is compatible with simultaneous interaction of both CUE domains with the experimentally defined interface areas of M1-linked diUb (blue). Residues involved in N4BP1 recognition are shown in yellow and the corresponding contact surface of the CUE domain is depicted in purple.

As discussed above, our initial pull-down experiments have already indicated that linear Ub chain binding specificity requires domains outside the C-terminal CUE domain which prompted us to assume the existence of a specificity module (LUBIN). To further understand the molecular basis of LUBIN interaction with M1-linked Ub, we speculated that the adjacent RNase domain forms a cooperative unit together with the C-terminal CUE domain. We therefore probed the interaction of this tandem domain fragment with various di-Ub linkages by SPR measurements (Fig. 7A–D). Remarkably, the extended construct that comprised the RNase and CUE domains (aa613-893) shows a 65-fold increase in M1-Ub binding, while all other tested Ub interactions display no significant differences when compared to the isolated CUE domain (Fig. 7E, Table 1). However, we did not observe any interaction between the isolated RNase domain (613-774) and Ub linkages (Table 1), which indicates that this domain is not directly involved Ub chain binding but at the same time further raises the question of how N4BP1 selective interaction of Met1-linked Ub chains is achieved. Intriguingly, N4BP1 constructs comprising the RNase domain had a strong tendency to self-associate in vivo and in vitro (Fig. S8A–D), suggesting that N4BP1 dimerizes through its RNAse domain. Interestingly, the protein MCPIP1, which shares 52% sequence identity with N4BP1, features the same oligomeric nature caused by the formation of an asymmetric dimer of its RNase domain [32]. A homology model of the N4BP1 RNase domain, based on the structure of the MCPIP1 RNase domain, shows that an N4BP1 dimer can be formed (Fig. S8E). To explain how N4BP1 dimerization creates selectivity towards linear linkages, we hypothesized that the spatial arrangement of the dimer brings the two adjacent CUE domains into close vicinity, which could result in the simultaneous interaction of the CUE domains with the same linear M1-linked diUb molecule (Fig. 7F). To further examine this scenario, we deployed a homology modelling and docking approach to generate a model of N4BP1 (613–893) in complex with M1-linked diUb, which aligns with the experimental constraints derived from our CSP experiments and mutational analysis of interface residues between the CUE domain and Ub. The refined model, which satisfies all experimental criteria, demonstrates that dimerization of N4BP1 via the RNase domain is well suited to generate a spatial composite arrangement of the CUE domains that permits binding of M1-diUb with high affinity (Fig. 7G).

## Discussion

Nepravishta et al. [22] have described the structural model of the CoCUN domain that encompasses the last 50 amino acids of human N4BP1 and binds to the I44 hydrophobic patch of monoUb, similar to our findings. Interestingly, related proteins MCPIP1-4 and KHNYN also contain divergent CUE domains (Fig. S1C), but only KHNYN CUE binds Ub [33].

MCPIP1 possesses ribonuclease activity within its PIN domain and plays a critical role in the inflammatory response by degrading mRNA of numerous cytokines [34]. MCPIP1 PIN domain undergoes head-to-tail intermolecular dimerization, enabling mRNA processing, and mutations preventing oligomerization abolish RNase activity [32]. N4BP1 was also recently identified as an interferon-inducible inhibitor of HIV-1 in primary T cells and macrophages, and shown to specifically degrade HIV-1 mRNA to control HIV-1 latency and reactivation [35]. KH domains found in many proteins bind nucleic acids [36] and it has been recently shown that KHNYN requires both its KH-like and NYN RNase domains for its antiviral activity [37]. It is however unclear if the N-terminal KH-like domains present in both KHNYN and N4BP1 are able to bind RNA. Future studies should systematically screen for specific N4BP1 RNase substrates and their potential role in NFκB signalling, as well as the role of its KH-like domain in both NFκB signalling and antiviral activity.

Two classes of structurally distinct LUBID interaction modes have been characterized so far [7]. The UBAN domains in NEMO, OPTN and ABIN form a parallel coiled-coil, which enables interactions with M1 linkages (Fig. S9A) [9]. In contrast, HOIL-1L and A20 utilize structural elements that adopt a zinc finger fold to recognize and bind linear Ub chains with high affinity (Fig. S9B) [38, 39]. Our findings indicate that N4BP1 utilizes the third and hitherto uncharacterized interaction mode, which depends on its RNase domain as a dimerization module (Fig. S9C). Homodimerization of N4BP1 elicits a specific orientation of the two CUE domains, which facilitates a formation of a highly stable complex with M1-linked diUb. Some CUE domains have been reported to self-associate and form functional dimers [40, 41]. In fact, our analytical gel filtration experiments show that the elution volume of the CUE domain corresponds to a molecular weight of 8.4 kDa (Fig. S6C, D), which is larger than the calculated molecular weight (5.4 kDa) of a monomer, thus indicating a tendency for self-association that potentially contributes to selective M1-Ub chain interaction of the N4BP1 dimer. However, our ITC data of the isolated CUE domain do not show a marked preference to bind to linear Ub over monoUb (Fig. 4). We therefore conclude that the potential of the CUE domain to dimerise is not sufficient to induce Ub chain binding specificity, which is only realised in the context of the N4BP1 constructs comprising the RNase domain. According to our knowledge, this is the first example of a UBD that does not display any Ub chain binding preference on its own, but can mediate a highly selective Ub chain interaction in combination with a dimerization module. Our results suggest a new combinatorial mechanism, which exploits the modular nature of domains with different functions to establish a high affinity interaction with M1-linked Ub chains.

Shi et al. [20] report that N4BP1 inhibits TLR-dependent activation of NFκB by interacting with NEMO. In the proposed model, N4BP1 blocks NEMO oligomerization by binding to its C-terminal oligomerization domain, which hinders IKKα/β recruitment and NFκB activation. Interestingly, several Ub-binding domains, i.e., UBA and CUE domains in N4BP1, as well as NEMO CoZi domain (containing UBAN) in NEMO are necessary for the binding, implying the role of ubiquitin linkages in their interaction. Together with our data, it is becoming increasingly evident that N4BP1 carries several important functions in TNFR1 signalling, including the regulation of NEMO and ubiquitin linkages to regulate the pathway.

Several LUBID-containing proteins also play an important role in TNFR1 signalling. UBAN-containing OPTN acts as a negative regulator of TNFR1 signalling upon TNFα stimulation, with linear Ub and CASP8 binding being critical for NFκB and apoptosis suppression respectively [42–44]. Abolished Ub binding by UBAN-containing protein ABIN-1 promotes NFκB and proinflammatory signalling, enhancing the production of proinflammatory mediators [45–47]. Furthermore, ABIN-1 prevents cell death by inhibiting CASP8 recruitment to FADD in UBAN-dependent manner [48], presumably by binding linear Ub-modified FADD that prevents it from forming complex II with CASP8 [11]. Ub-editing enzyme A20 is recruited to TNFR1-SC through its M1- (ZnF7) and K63-specific (ZnF4) UBDs [49, 50]. By protecting linear Ub chains from DUB cleavage, A20 prevents formation of the complex II [50]. These data are in agreement with our results, showing how distinct linear Ub readers within TNFR1-SC regulate TNFR1 signalling, as well as with data showing how deficiency of LUBAC components promotes complex II assembly and induces aberrant TNF-mediated endothelial cell death [51].

Gitlin et al. [19] identified N4BP1 as a suppressor of cytokine production that is inactivated by CASP8, similar to our findings. Furthermore, they could also demonstrate that the major CASP8 cleavage site in N4BP1 is residue D488. Mice lacking N4BP1 show increased production of a subset of cytokines, which is in agreement with our data. We here provide a detailed mechanism on how N4BP1 exerts its activity in TNFR1 signalling through the newly identified LUBIN, and explain how CASP8 cleavage leads to removal of functional LUBIN that is no longer able to stabilize linear Ub linkages and the integrity of TNFR1-SC. Similarly, HOIP is also cleaved upon the induction of apoptosis and subjected to proteasomal degradation. Whereas the C-terminal fragment of HOIP retains NFκB activity, linear ubiquitination of NEMO and FADD are decreased [11], facilitating the assembly of complex II and apoptosis. It was also shown that CASP8 inhibitor cFLIP is modified by LUBAC to prevent its proteasomal degradation. Inactivation or depletion of HOIP leads to the removal of cFLIP, releasing CASP8 inhibition [52]. This implies that multiple proapoptotic regulators are kept in check by linear Ub chains generated by LUBAC and that the removal of these linkages unleashes apoptosis.

In summary, we propose that N4BP1 is recruited to the nascent TNFR1-SC upon TNFα stimulation, where it binds linear Ub linkages via LUBIN to regulate the integrity of TNFR1-SC and duration of proinflammatory signalling (Fig. S10). Prolonged TNFα stimulation leads to the activation of CASP8 that cleaves N4BP1. After the C-terminal part of N4BP1 is removed by 26 S proteasome, complex II formation and apoptosis are accelerated. Thus, N4BP1 utilizes a complex mechanism to contribute to tight regulation of the progression of the TNFα-mediated NFκB pathway and cell death.

## Materials and methods

### Cell lines

Cell line HEK293T was purchased from ATCC. Wild-type and CASP8-deficient Jurkat cells were generated by John Blenis (USA) and Clarissa von Haefen (Germany). Primary N4BP1^+/+^ and N4BP1^−/−^ MEFs, kindly provided by Michael Kuehn (USA), were immortalized by transfection with plasmid containing SV40 T-antigen. All cells were regularly checked for *Mycoplasma* infection using VenorGeM Classic from *Minerva Biolabs GmbH* (Ltd).

### Reagents

Puromycin and zeocin were from *Invivogen*. Ac-DEVD-cmk, z-IETD-fmk and z-VAD-fmk were from *Santa Cruz Biotechnology*. TNFα and IL-1β were from *PeproTech*. Polybrene and benzonase endonuclease were from *Merck*. Phosphatase and Protease Inhibitor Cocktails were from *Roche Applied Science*. Crystal violet was from *Carl Roth GmbH* and cycloheximide (CHX) from *Enzo Life Sciences*.

### Plasmids and antibodies

The lists of used plasmids and antibodies are available in Tables S1 and S2, respectively. In hexaUb YTH9 plasmid, five tandem Ub molecules had a terminal GG motif mutated to GV (to prevent cleavage by cellular DUBs during the screen), whereas the proximal Ub lacked GG motif (to prevent its potential conjugation to yeast proteins). For SPR experiments, the N4BP1 (613-774) and N4BP1 (613–893) fragments were cloned into pCold-TF (*Takara*) vector. For analytical gel filtration, the N4BP1 (613-774) and N4BP1 (613–893) fragments were cloned into pCold-I (*Takara*) vector, which include an N-terminal 20 amino acid solubility tag (GGGTPKAPNLEPPLPEEEKEG).

### Yeast two-hybrid

Gal4BD-fused hexaUb plasmid was transformed into Y2HGold *Saccharomyces cerevisiae* strain (*Clontech*) and used as bait in Y2H screen, where it was mated with the human normalized cDNA library (*Clontech*) transformed into Y187 yeast strain (prey). Four independent reporter genes (*AUR1-C*, *ADE2*, *HIS3*, and *MEL1*) were used for selection according to the manufacturer’s instructions. Clone identities were determined by Sanger sequencing (*Microsynth Seqlab*).

### Bioinformatics analysis

Multiple alignments were calculated using the L-Ins-I algorithm of the MAFFT package [53]. Sequence database searches were performed by the generalized profile method [54], using the pftools package. Inter-family similarities were established by Hidden Markov Model (HMM) to HMM comparisons, using the HHSEARCH programme package [55].

### Transfection of mammalian cells

HEK293T cells were transiently transfected with either polyethylenimine (PEI, *Polysciences*) or GeneJuice transfection reagent (*Merck Millipore*). Non-adherent Jurkat cells were electroporated using the Neon Transfection System (*Invitrogen*). MEFs were transiently transfected using GenJet In Vitro DNA Transfection Reagent (*SignaGen Laboratories*). All the transfections were performed according to the manufacturer’s instructions. If the experiment required transfection of several plasmids simultaneously, appropriate amounts of empty vectors were used to equalize the total plasmid amount across the samples.

### Retroviral production

For reconstitution of N4BP1^−/−^ MEFs, various pBabe plasmids (carrying resistance to either puromycin or zeocin) were used. Twenty-four hours after seeding, HEK293T cells were transfected with appropriate plasmid DNA and helper plasmid phai by using GeneJuice. Thirty-six hours post-transfection, DMEM containing retroviruses was filtered, mixed with polybrene (final concentration 4 μg/ml) and transferred to target cells. Forty-eight hours post-infection, selection with either 300 μg/ml of zeocin or 4 μg/ml of puromycin was started. Cells were cultured in the presence of the appropriate antibiotics throughout use.

### Generation of N4BP1 KO MEFs by CRISPR-Cas9

N4BP1 KO^CRISPR^ MEFs were generated by transducing immortalized N4BP1^+/+^ MEFs with the lentiviral particles generated with the modified lentiCRISPRv2 (#52961, Addgene) single vector system [56]. Single gRNA against mouse N4BP1 (GAGTTGCAGCCAGATACGCG) was selected with the Azimuth 2.0 tool of the GPP sgRNA Designer and cloned into BsmBI site of the vector. Polyclonal N4BP1 KO^CRISPR^ MEFs were selected with 2 µg/ml puromycin for 14 days and the obtained cell line was validated by Western blotting.

### Various treatments of mammalian cells

Where indicated, starved cells were treated with either recombinant human IL-1β, mouse TNFα or human HS-TNFα at concentrations 10 ng/ml, 20 ng/ml and 1 μg/ml, respectively. To identify N4BP1 cleavage sites, serum-starved HEK293T cells were treated with recombinant mouse TNFα (20 ng/ml) and additionally, with proteasome inhibitor MG132 (*Tocris Bioscience*) at 10 μM final concentration. For identification of the specific CASP responsible for N4BP1 proteolysis, MEF N4BP1^+/+^ and HEK293T cells were treated with recombinant mouse TNFα (20 ng/ml) and 20 μM of either CASP inhibitors z-VAD-fmk, z-IETD-fmk or Ac-DEVD-cmk. For testing N4BP1 proteolysis in Jurkat cells, TNFα was used at concentration 100 ng/ml. For cell death induction, recombinant mouse TNFα and cycloheximide (CHX) were used at concentration 10 ng/ml and 0.5–1 μg/ml, respectively. For inhibition of apoptosis, cells were treated with mouse TNFα (10 ng/ml), CHX (1 μg/ml) and z-VAD-fmk (20 μM). The duration of treatments is indicated in the figures.

### Expression and purification of recombinant proteins

Various GST fusions were purified as shown previously [57]. HS-TNFα was expressed in *E. coli* with an N-terminal STREP II tag and purified on Strep-tactin Sepharose according to manufacturer’s instructions (*GE Healthcare*), followed by gel filtration using Superdex S75 (*GE Healthcare*) and subsequent dialysis against PBS. Recombinant active and inactive (C360S) CASP8 were purified from BL21 *E. coli* by lysing bacterial pellet in 50 mM Na_2_HPO_4_/NaH_2_PO_4_ (pH 7.4), 300 mM NaCl, 20 mM imidazole, 30% glycerol and 20 mM β-mercaptoethanol (β-Me). Precleared lysates were bound to nickel resins (*Qiagen*) and washed several times with lysis buffer, after which proteins were eluted with 50 mM Na_2_HPO_4_/NaH_2_PO_4_ (pH 8.0), 300 mM NaCl, 250 mM imidazole, 30% glycerol and 20 mM β-Me. N4BP1 proteins for SPR and ITC measurements were expressed and purified from BL21 *E. coli* using GST or immobilized cobalt affinity chromatography followed by size exclusion chromatography and anion exchange chromatography, if required. Size exclusion chromatography was performed in 50 mM HEPES (pH 7.4), 150 mM NaCl and 1 mM DTT, using Superdex S200 or Superdex S75 chromatography columns *(GE Healthcare)*. For anion exchange chromatography, protein samples were separated by varied gradient elution from MonoQ columns *(GE Healthcare)* using 20 mM HEPES (pH 8.5), 1 mM DTT, 0.5% Triton X-100 and 0.1–1.0 M NaCl. For NMR spectroscopy ^15^N/^13^C-labelled N4BP1 (850–893) was expressed in M9 media (6 g/L Na_2_HPO_4_, 3 g/L KH_2_PO_4_, 0.5 g/L NaCl, 0.7 g/L ^15^NH_4_Cl, 2 g/L ^13^C-D-glucose, 10 mL/L 100X MEM vitamin solution *(Gibco)*, 10 μM FeSO_4_, 10 μM CaCl_2_, 2 mM MgSO_4_, pH 7.4). For ITC and NMR experiments, the His-tag was removed from isolated N4BP1 CUE domain by incubation with 3 C protease at 4 °C, overnight. For analytical gel filtration, the His-tag of purified N4BP1 fragments was not removed. Enzymatic synthesis and purification of K48- and K63-linked Ub was essentially carried out as described [58].

### Analytical size exclusion chromatography

Analytical size exclusion chromatography was performed using a Superose^TM^ 12 10/300 L column (*GE Healthcare*) calibrated with the 29,000–700,000 Da GF Marker Kit (*Sigma-Aldrich*). Protein samples were eluted in 50 mM HEPES, pH 8.5, 150 mM NaCl, 1 mM DTT, 0.5% Triton X-100.

### Isothermal titration calorimetry (ITC)

ITC experiments were performed at 293 K using a Microcal PEAQ-ITC calorimeter (*Malvern*). The protein solutions were prepared in a buffer containing 50 mM HEPES (pH 7.4), 50 mM NaCl and 0.5 mM TCEP. Experiments were performed at cell at concentrations 50–100 μM. The injectant concentration in the syringe was usually 10-fold to the titrant. For each titration 20 injections of 2 μl were performed. Integrated data, corrected for heats of dilution, were fitted using a nonlinear least-squares algorithm to obtain a binding curve, using the MicroCal Origin 7.0 software package. Each experiment was repeated at least twice, and average values are reported in Table 1.

### In vitro CASP8 cleavage assay

C-terminally FLAG-tagged N4BP1 was transiently transfected into HEK293T cells, which were washed in 1xPBS 24 h after transfection. Cells were lysed in 1% Triton X-100 buffer (50 mM Tris-HCl, pH 7.5; 40 mM NaCl; 5 mM EDTA; 1% Triton X-100; 1x Protease inhibitor cocktail) and incubated on ice for 20 min. Precleared lysates were incubated with M2 agarose (*Sigma-Aldrich*) at 4 °C for 2 h. After 5 washing steps with modified 1% Triton X-100 buffer (50 mM Tris-HCl, pH 7.5; 500 mM NaCl; 5 mM EDTA; 1% Triton X-100; 1x Protease inhibitor cocktail) and two washing steps with FLAG elution buffer (20 mM Tris-HCl, pH 7.5; 150 mM NaCl; 0.2 mM EDTA; 0.1% Triton X-100; 15% glycerol), FLAG-tagged N4BP1 was eluted from M2 resins with FLAG peptide at concentration 3 μg/ml. Thirty microliters of eluate were incubated with 1 μg of either active or inactive (C360S) 6xHIS-tagged CASP8 cleavage assay buffer (50 mM HEPES, pH 7.2; 50 mM NaCl; 10 mM EDTA; 5% glycerol; 10 mM DTT) at 37 **°**C for 2 h.

### GST and MBP pull-down assays

Pull-down assays were performed as described in [59]. Since the size of FLAG-N4BP1 (613–893) protein was identical to the size of GST diUb, GST diUb beads were additionally cleaved with 1U thrombin (*GE Healthcare*) in 1x thrombin cleavage buffer (20 mM TrisCl, pH 8.4; 150 mM NaCl; 2.5 mM CaCl_2_; 1 mM DTT) at 25 **°**C for 4 h after GST PD wash.

### Pull-down of TNFR1-SC

For isolation of TNFR1-SC complex, cells were stimulated in the presence or absence of HS-TNFα at concentration 1 μg/ml for 5 min. Then, cells were washed twice with ice-cold 1xPBS, lysed in TNFR1-SC lysis/PD buffer (20 mM Tris-HCl, pH 7.5; 150 mM NaCl, 1% Triton X-100; 10% glycerol; 2 mM NEM, 1x Protease inhibitor cocktail, 1x Phosphatase inhibitor cocktail) and incubated at 4 °C for 30 min. Lysates were cleared by centrifugation (15,000 rpm, 4 °C, 30 min) and supernatants precleared with Superflow resin (*IBA GmbH*) at 4 °C for 30 min with rotation. HS-TNFα (1 μg) was added to non-stimulated control and lysates were incubated with prewashed Strep-tactin XT resin (*IBA GmbH*) for 2 h at 4 °C. Next, samples were washed 7 times with TNFR1-SC lysis/PD buffer, re-suspended in 1xLDS buffer supplemented with β-Me and denatured at 70 °C for 10 min.

### Ubiquitin-binding assays

One microgram of monoUb and synthetic diUb chains (*UbiQ Bio*) were incubated with indicated GST protein fusions bound to Glutathione Sepharose 4B resin in incubation buffer (50 mM HEPES pH 7.5; 150 mM NaCl; 1 mM EDTA; 1 mM EGTA; 1% Triton X-100; 10% glycerol and 1 mM DTT) for 2 h at 4 °C. Next, samples were washed four times with incubation buffer prior to elution in 1xLDS buffer supplemented with β-Me.

### Surface plasmon resonance (SPR)

The interactions of TF-N4BP1 (613–893) with monoUb and M1-, K48- and K63-linked diUb were analysed by SPR, using a Biacore S200 (*GE Healthcare*). Experiments were performed in HBS-P^+^ buffer (10 mM HEPES, pH 7.4, 150 mM NaCl, 0.05% Tween 20) at 25 °C. TF-N4BP1 (613–893) (10 μg/ml) was covalently immobilised on a CM5 chip using the amine-coupling kit (*GE Healthcare*) according to the manufacturer’s instructions. Mono- and diUb proteins were dialyzed into HBS-P^+^ buffer prior to the experiments. Experiments were run with a concentration series of mono- and diUb at 30 μl/min with 20 s association and 30 s dissociation phases. Association and dissociation kinetics were too fast to be resolved in these experiments. Data analysis was therefore performed by analysing the plateau levels. K_D_ values were obtained from non-linear least-square fitting using a hyperbolic binding equation in GraphPad Prism 8.

### Immunoprecipitation experiments

For HA IP, cells were washed twice in ice-cold 1x PBS, lysed in HA lysis/IP buffer (20 mM Tris-HCl, pH 7.5; 150 mM NaCl; 0.5% sodium deoxycholate; 1% NP-40; 2 mM NEM; 1 mM PMSF; 1x Protease inhibitor cocktail) and incubated with benzonase endonuclease (4 °C, 30 min). Lysates were centrifuged (13,000 rpm, 4 °C, 15 min), followed by Sepharose CL-4B (*Sigma-Aldrich*) preclearing at 4 °C for 30 min. Then, lysates were incubated with prewashed monoclonal anti-HA agarose (clone HA-7, *Sigma-Aldrich*) for 3 h at 4 °C, with agitation. Samples were washed three times with denaturing buffer and twice with 1x PBS, re-suspended in 1xLDS buffer supplemented with β-Me and denatured at 70 °C for 10 min.

The procedure for FLAG IP was described in in vitro CASP8 cleavage assay protocol. Linear Ub IP was performed as described previously [60].

For endogenous IP cells were washed twice with ice-cold 1x PBS and lysed in endogenous IP buffer (20 mM Tris-HCl, pH 7.5; 150 mM NaCl; 1% Triton X-100; 2 mM NEM; 1x Protease inhibitor cocktail, 1x Phosphatase inhibitor cocktail) on ice for 30 min. Lysates were collected and centrifuged (13,000 rpm, 4 °C, 15 min), followed by incubation with the antibody recognizing desired protein and protein A/G Sepharose (*Sigma-Aldrich*) at 4 °C for either 4 h or overnight. Then, samples were washed four times with an endogenous IP buffer, and eluted by incubation in a 1xLDS buffer supplemented with β-Me (70 °C, 10 min).

### Immunoblotting

Immunoblotting was performed as in ref. [59]. Proteins were first separated by SDS-PAGE and transferred to either 0.22 μm (*Santa Cruz Biotechnology*) or 0.45 μm nitrocellulose membrane (*NitroBind, Maine Manufacturing*) using *Bio-Rad* apparatus for wet blotting. Transfer was performed in 1x transfer buffer (25 mM Tris; 190 mM glycine; 20% methanol), at constant amperage of 200 mA for 2 h. Next, membranes were either stained with 0.5% (m/v) Ponceau solution at RT for 20 min or directly blocked in either 5% BSA in TBS-T (20 mM Tris-HCl, pH 7.6; 150 mM NaCl; 0.1% Tween 20) or in 5% milk solution in TBS-T at RT for 1 h. Incubation with indicated primary antibodies was carried out either at 4°C (overnight) or at RT for 1–2 h. List of antibodies is available in the Table S2. Then, membranes were washed three times with TBS-T for 10 min, incubated with appropriate HRP-conjugated secondary antibodies at RT for 1 h, followed by three washing steps in TBS-T and TBS (20 mM Tris-HCl, pH 7.6; 150 mM NaCl). After incubation of membranes in either Western Blotting Luminol reagent (*Santa Cruz Biotechnology*) or Lumigen TMA-6 (*GE Healthcare)*, protein signals were detected with Super RX-N X-Ray films (*FUJIFILM Corporation*) by using CURIX 60 developing unit (*AGFA*).

For linear Ub IP, immunoblotting was performed as described previously [60]. Eluates containing immunoprecipitated linear polyUb-modified proteins were separated on 4–20% gradient PAGEr^TM^ Gold gels (*Lonza*), transferred onto 0.22 μm nitrocellulose membranes by wet blotting at 30 V for 2 h, blocked in 5% milk solution in PBS-T at RT for 1 h, followed by incubation with 1F11/3F5/Y102L IgG dissolved in 5% milk solution in PBS-T at RT for 1 h. Membranes were washed three times with PBS-T, incubated with secondary HRP-conjugated goat anti-human antibody (RT, 1 h), washed four times with PBS-T and visualized as described above. For linear Ub IPs (where endogenous levels of modified proteins were visualized), as well as for endogenous co-IPs, Clean-Blot IP Detection Reagent (*Thermo Fisher Scientific*) was used instead of secondary HRP-conjugated antibodies.

### Preparation of IP samples for MS analysis

For identification of cleavage sites in N4BP1, cells were transfected with plasmids encoding either FLAG-N4BP1 or N4BP1-FLAG. Twenty-four hours later, cells were washed twice in ice-cold 1x PBS, followed by lysis in denaturing buffer (20 mM Tris-HCl, pH 7.5; 150 mM NaCl; 1 mM EDTA; 0.5% NP-40; 0.5% sodium deoxycholate; 0.5% SDS; 1 mM DTT; 2 mM NEM; 1x Protease inhibitor cocktail; 1x Phosphatase inhibitor cocktail). After benzonase treatment (4°C, 30 min), lysates were cleared by centrifugation (13,000 rpm, 4 °C, 15 min). Next, lysates were incubated with prewashed M2 resins (*Sigma-Aldrich*) for 5 h at 4 °C, with agitation. Samples were washed three times with denaturing buffer and twice with distilled water and re-suspended in 1xLDS buffer supplemented with β-Me and denatured at 70 °C for 10 min.

### Mass spectrometry analysis of N4BP1 cleavage

After elution and denaturation, samples were resolved by SDS-PAGE and gel lanes were cut into 7 slices, reduced with 200 μl of 10 mM DTT, alkylated with 200 μl of 55 mM chloroacetamide and digested with trypsin (final concentration 20 μg/ml) at 750 rpm, 37 **°**C, overnight. Peptides were bound to C_18_ StageTips and separated on EASY-nLC 1000 UHPLC (*Thermo Fisher Scientific*) connected to Q-Exactive HF Hybrid Quadrupole-Orbitrap (*Thermo Fisher Scientific*) mass spectrometer. For peptide separation, 15 cm and 75 μm ID PicoTip fused silica emitters (*New Objective*) were used. Emitters were self-made packed with ReproSil-Pur C18-AQ 3 μm resin (*Dr. Maisch GmbH*). Elution of the peptides from the column was performed using a linear gradient of 7–38% solvent B (80% acetonitrile in 0.1% formic acid) in 20 min with subsequent increase up to 95% solvent B within 5 min, followed by re-equilibration to 5% solvent B. Mass spectrometer was operated in positive ion mode and MS spectra were acquired with following settings: a maximal injection time of 20 ms and a 60,000/15,000 resolution at 200 m/z. Up to 15 most intense ions were selected for collision induced dissociation (CID) fragmentation. Data analysis was performed by using the MaxQuant software suite (version 1.5.3.30) and the internal search engine Andromeda and searched against the Uniprot *Homo sapiens* (released 2016) database. For the identification of cleavage sites in N4BP1, semi-specific tryptic peptides were searched for. Oxidation (M) and acetylation (protein N-terminus) were searched as variable modifications, whereas Cys carbamidomethylation (C) was set as fixed modification. Initial precursor mass tolerance was set to 4.5 ppm and MS/MS mass tolerance to 0.5 Da. Peptide and protein FDR (false discovery rate) was defined to 1%.

### Subcellular fractionation

The cellular fractionation was performed as in [59]. For subcellular fractionation of HEK293T cell line, cells were washed once with ice-cold 1x PBS and re-suspended in ice-cold Fractionation buffer A (10 mM HEPES, pH 7.8; 10 mM KCl; 0.1 mM EDTA; 0.5% Triton X-100; 1 mM DTT; 1 mM PMSF; 1x Protease inhibitor cocktail; 1x Phosphatase inhibitor cocktail). After 10 min incubation on ice, cell suspension was centrifuged (2000 rpm, 4 **°**C, 5 min). Supernatant was designated as cytoplasmic fraction. The remaining cell pellet was washed twice in ice-cold buffer A. Next, cell pellet was re-suspended in Fractionation buffer C (50 mM HEPES, pH 7.8; 420 mM KCl; 0.1 mM EDTA; 5 mM MgCl_2_; 10% glycerol; 1 mM DTT; 1 mM PMSF; 1x Protease inhibitor cocktail; 1x Phosphatase inhibitor cocktail) by passing several times through narrow-gauge syringe, followed by 30 min incubation on ice and centrifugation (13,000 rpm, 4 **°**C, 15 min). Supernatant was designated as nuclear fraction.

For subcellular fractionation of MEFs, cells were washed twice with ice-cold 1x PBS, collected and centrifuged (800 rpm, 4 **°**C, 5 min). After centrifugation, supernatant was aspirated and cell pellet was gently re-suspended in Isotonic lysis buffer (10 mM Tris-HCl, pH 7.5; 300 mM sucrose; 2 mM MgCl_2_; 3 mM CaCl_2_; 1x Protease inhibitor cocktail; 1x Phosphatase inhibitor cocktail). After incubation on ice, cell suspension was centrifuged (800 rpm, 4 **°**C, 5 min). Supernatant was discarded and pellet was re-suspended in Isotonic lysis buffer by passing several times through narrow-gauge needle. Then, cell suspension was again centrifuged (13,000 rpm, 4 **°**C, 20 min). Supernatant was collected and designed as cytoplasmic fraction, while pellet was re-suspended in Extraction buffer (20 mM HEPES, pH 7.9; 420 mM NaCl; 0.2 mM EDTA; 25% glycerol; 1.5 mM MgCl_2_; 1 mM DTT; 1x Protease inhibitor cocktail; 1x Phosphatase inhibitor cocktail) by passing several times through narrow-gauge needle. Suspension was incubated on ice for 30 min with occasional shaking. After centrifugation (13,000 rpm, 4**°**C, 20 min), supernatant was designated as nuclear fraction.

### Luciferase assay

The assay was performed as in [59]. Measurements were done by using either a Wallac Victor^3^ 1420 Multilabel plate reader (*Perkin Elmer*) or Synergy H1 Hybrid Multi-mode reader (*BIOTEK*). All experiments were done at least in biological quadruplicate, where each biological replicate consisted of technical duplicate.

### Real-time PCR

The quantitative real-time PCR was performed with SensiMix SYBR & Fluorescein kit (*Bioline*) in the iCycler iQ5 Real-Time PCR Detection System (*Bio-Rad*). GAPDH was used as an internal control. The Comparative Ct (Threshold Curve) method was used for the quantification of the amount of target, normalized to internal control. List of oligonucleotides is available in the Table S3. Experiment was performed in biological triplicates, where each biological replicate consisted of technical duplicates.

### Immunofluorescence

Cells were seeded on glass coverslips and treated as indicated in the text. Cells were fixed by using 2% PFA, permeabilized with PBS containing 0.2% Triton X-100, blocked in 5% BSA in PBS solution at RT. Next, cells were incubated with appropriate antibodies. Coverslips were mounted with Mowiol containing DAPI, in order to visualize nuclei. Images were acquired with LEICA TCS SP8 confocal laser microscopy. For quantification, at least 200 cells were counted per condition.

### Crystal violet assay

The assay was performed as in ref. [61]. Optical density at 570 nm (OD_570_) was measured by using Wallac Victor^3^ 1420 Multilabel plate reader (*Perkin Elmer*). Experiment was performed in biological triplicates, where each biological replicate consisted of technical quadruplicates.

### Luminescent cell viability assay

Cell viability was determined by using CellTiter-Glo Luminescent Cell Viability Assay kit (*Promega GmbH*) according to manufacturer’s instructions. Briefly, 96-well plate was equilibrated at RT for 30 min, followed by addition of 100 μl of CellTiter-Glo Reagent provided by the manufacturer. Cell lysis was initiated by shaking a 96-well plate for 2 min. To achieve signal stabilization, 96-well plate was additionally incubated at RT for 10 min. Measurement was performed by using Synergy H1 Hybrid Multi-mode reader (*BIOTEK*). Experiments were performed in biological triplicates, where each biological replicate consisted of technical duplicates.

### NMR spectroscopy

All NMR samples (300-500 μM) were prepared in 20 mM phosphate buffer (pH 7.0), 50 mM NaCl, 1 mM DTT, 10% D_2_O. For atom assignments, N4BP1 CUE domain was uniformly ^15^N,^13^C-labelled and assignments were completed using standard triple-resonance assignment methodology [62]. A total of 97% of the potential backbone (disregarding the proline residues) and 87% of the potential side-chain resonances were assigned (the first 3 N-terminal residues from the tag are ignored). Titration experiments involving ^15^N-labelled N4BP1 CUE domain were performed by addition of up to 5 molar equivalents of unlabelled Ub. Titration experiments involving ^15^N-labelled Ub were performed by addition of up to 5 molar equivalents of unlabelled N4BP1 CUE domain. The magnitude of chemical shift perturbations (CSPs) for each resonance was quantified according to the equation Δ${\rm{\delta }}$ = ((${\rm{\delta }}$^H^_bound_-${\rm{\delta }}$^H^_free_)^2^ + ((${\rm{\delta }}$^N^_bound_-${\rm{\delta }}$^N^_free_)/a)^2^)^1/2^, where a = (${\rm{\delta }}$^N^_max_- ${\rm{\delta }}$^N^_min_)/ (${\rm{\delta }}$^H^_max_- ${\rm{\delta }}$^H^_min_). NMR experiments were performed on two types of Bruker spectrometers, an AvanceNEO 600 equipped with a 5 mm ^1^H/^13^C/^15^N inverse triple resonance probe and an Avance III HD 700, equipped with a 5 mm ^1^H/^13^C/^15^N triple-resonance PFG cryoprobe. All spectra were collected at 303.15 K. Data were processed using NMRPipe [63] and analysed using CcpNmr Analysis V2 [64].

### NMR structure determination

An experimentally guided model of N4BP1 CUE domain was generated with NMR chemical shift data in combination with homologous structural information using the standard CS-Rosetta method [65, 66]. Backbone chemical shift data (C^α^, C^β^, C’, N, H^α^ and HN) was included and a total of 20,000 models were generated. The top 10 models with the lowest energy were chosen as the final ensemble. Structural statistics were calculated using several servers including wwwPDB, MolProbity and PROSESS. Favourable Ramachandran statistics were observed, with 100% of residues in most favoured (98%) regions and 0% in outlier regions (Table S4).

### Molecular modelling

The dimer of the C-terminal portion for the mouse sequence of N4BP1 was modelled with Robetta [67] (Comparative Modelling mode). The modelled region encompasses the RNase and CUE domains including their joining linker (residues 613–893 in the UniProt sequence Q6A037). The X-ray structure of the MCPIP1 dimer (PDB ID: 5H9W) was used as template for the dimer of the RNase domains (sequence identity = 52%), while the NMR structure from this work was used for CUE domain. Sequence alignments were generated using PRALINE [68]. Multiple models were generated, which showed high variability in the relative arrangement of the CUE domains with respect to each other and to the RNase domains. This was consistent with the partially disordered and thus highly flexible nature of the linker domain (residues 776-849) as predicted by DISOPRED3 [69]. Correspondingly, the estimates of model local error were generally low for the RNase domain (1.2 Å on average) and high for the linker (> 20 Å). The best model was selected to have a distance between the centres of mass of the two CUE domains compatible with a simultaneous binding to M1-linked diUb (~ 31 Å).

A model of the CUE/monoUb interface was built using HADDOCK2.4 [70] with default parameters. The structure of monoUb was taken from PDB ID: 1UBQ. CSP values were used to define the Ambiguous Interaction Restraints for the calculation. In particular, residues with CSP values greater than the average value calculated over each molecule and with a relative solvent accessible surface area (SASA) larger than 30% were set as active residues. SASA values were calculated using GetArea [71]. The solution with the best HADDOCK score (Z-score = −1.3) was also the one most consistent with the interface model emerging from the experimental data from this work and in particular with the involvement of the nonpolar interface formed between the hydrophobic patch surrounding I44 of Ub and the FP motif of the CUE domain, as well as the polar contact between K48 of Ub and D893 of N4BP1.

The final model of the N4BP1 dimer bound to the M1-linked diUb was built with MODELLER 9.15 [72]. A template of the CUE/diUb complex was built by superimposing a copy of the CUE/monoUb best model from HADDOCK on each Ub molecule in the experimental structure of M1-linked diUb (PDB ID: 2W9N). The best Robetta structure (see above) was used as a template for the N4BP1 dimer. For each MODELLER run, 100 structures were generated and the one with the lowest DOPE score was selected as the final structure.

The resulting N4BP1/M1-linked diUb model was refined by energy minimisation using GROMACS 2016.3 [73]. The system was solvated using a truncated octahedral box of TIP3P water molecules. A minimal distance of 12 Å was set between the protein and the walls of the box. The proteins were described with the Amber99SB*-ILDN [74] force field. The charge of the ionisable residues was set to that of their standard protonation state at pH 7, the systems were then neutralised by adding counter-ions. Each system was minimised through 3 stages with 7000 (positional restraints on heavy atoms) + 5000 steps of steepest descent, followed by 2000 steps of conjugate gradient. The quality of the refined models was evaluated using MolProbity [75]. The refined model had a MolProbity score <=1.62 (92nd percentile) and a clashscore <= 0.7 (99^th^ percentile).

### Statistical analysis

To determine statistical significance in Fig. S2E, an unpaired, two-tailed Student’s *t* test was used. Three independent experimental replicates consisting of technical duplicates were performed. To determine statistical significance in Fig. S2C, a two-way ANOVA test was used. Five independent experimental replicates consisting of technical duplicates were performed. To determine statistical significance in Figs. 2E, 3A, 3C and S2F, a two-way ANOVA test, *post hoc* Sidak’s multiple comparisons test was used. Three independent experimental replicates consisting of technical duplicates were performed in Fig. 2E. Three independent experimental replicates consisting of technical triplicates were performed in Fig. 3C. Three independent experimental replicates consisting of technical quadruplicates were performed in Fig. 3A. To determine statistical significance in Fig. 3F, a two-way ANOVA test, *post hoc* Tukey’s multiple comparisons test was used. Three independent experimental replicates consisting of technical duplicates were performed. For all of the figures, results are shown as means and error bars defined as s.e.m. ****P < 0.0001, ***P < 0.001, **P < 0.01, *P < 0.05 were considered significant, while *P* > 0.05 was considered nonsignificant. No data were excluded for analysis.

## Supplementary information

Supplemental Figures

Supplemental Table

Original Data File

## Data Availability

Coordinates of the structure of N4BP1-CUE and the N4BP1-CUE/Ub complex have been deposited in the PDB-Dev Protein Data Bank (https://pdb-dev.wwpdb.org) under accession codes PDBDEV_00000076 and PDBDEV_00000093, respectively. Chemical shift data have been deposited in the Biological Magnetic Resonance Data Bank (https://bmrb.io) with BMRB entry ID 50688. The model of dimeric N4BP1 in complex with linear Ub2 is available in ModelArchive (modelarchive.org) with the accession code ma-2x3cw. The mass spectrometry proteomics data have been deposited to the ProteomeXchange Consortium via the PRIDE partner repository [76] with the dataset identifier PXD024355. All the plasmids generated in this study will be available upon request. All data is available in the main text or the supplementary materials. All original western blot images are available in the Supplemental Material.
