# Supplementary material for: N4BP1 functions as a dimerization-dependent linear ubiquitin reader which regulates TNF signalling
Source: Cell Death Discov. 2024 Apr 20;10:183. doi: 10.1038/s41420-024-01913-8 (PMC11032371; doi:10.1038/s41420-024-01913-8)
Supplement: Supplementary file 1 — Supplemental Figures [file 41420_2024_1913_MOESM1_ESM.pdf]

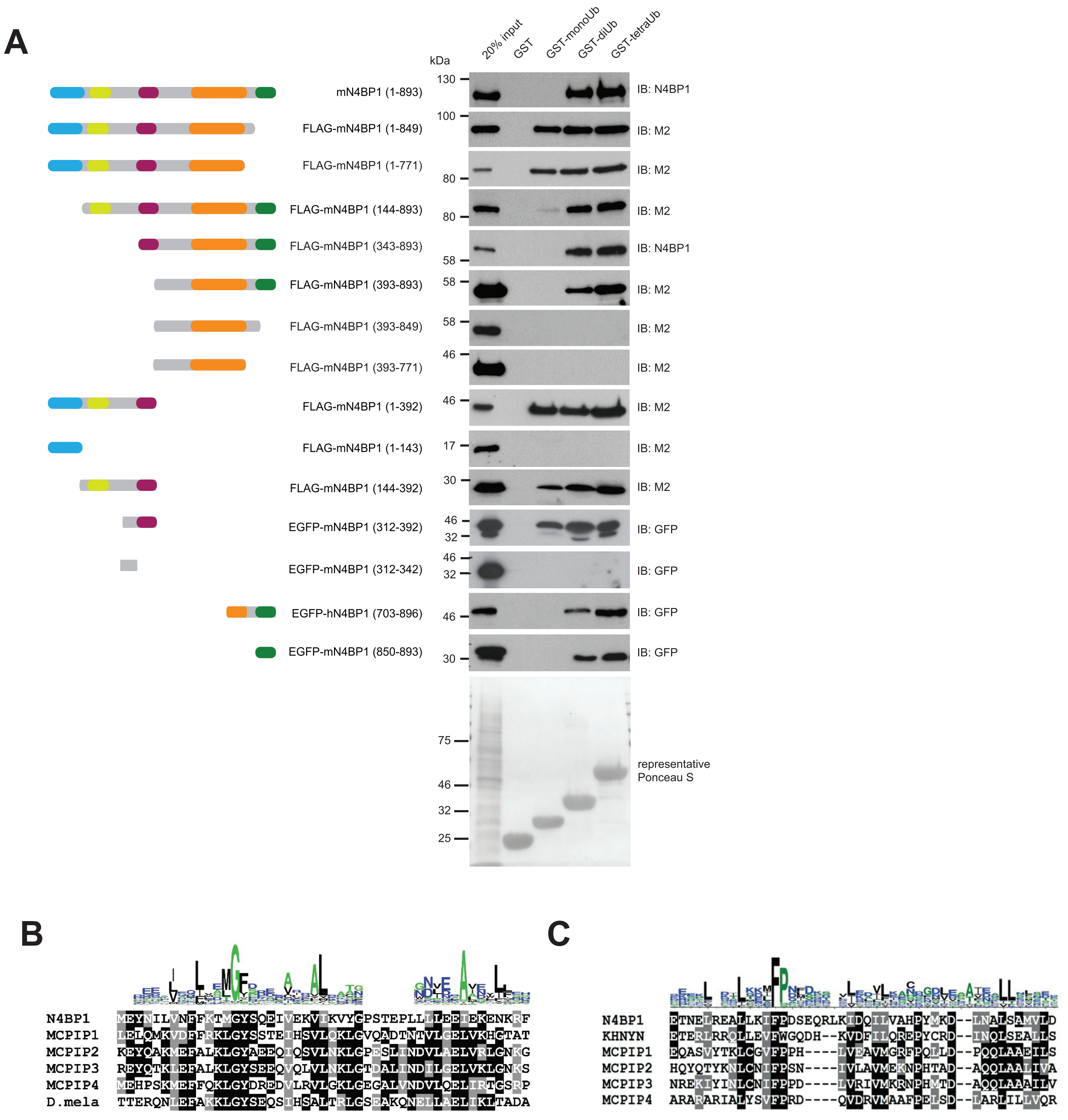

Fig. S1 N4BP1 is a novel linear ubiquitin receptor. A GST pull-down assay with various fragments of N4BP1 transiently overexpressed in HEK293T cells with GST fusions of mono-, di- and tetraUb. GST alone was used as negative binding control. **B** Alignment of N4BP1 UBA domain with classical UBA domains and SeqLogo of the UBA domain consensus. The height of each amino acid is proportional to its conservation at a respective position. The alignment is rendered by box shade (residues invariant or conservatively replaced in at least 50% of the sequences are shown on black or grey background, respectively). **C** SeqLogo of the CUE domain consensus; amino acid frequencies have been derived from an alignment of established CUE domains. Alignment of N4BP1 CUE domain shows residues invariant or conservatively replaced in at least 50% of the sequence shown on black or grey background, respectively.

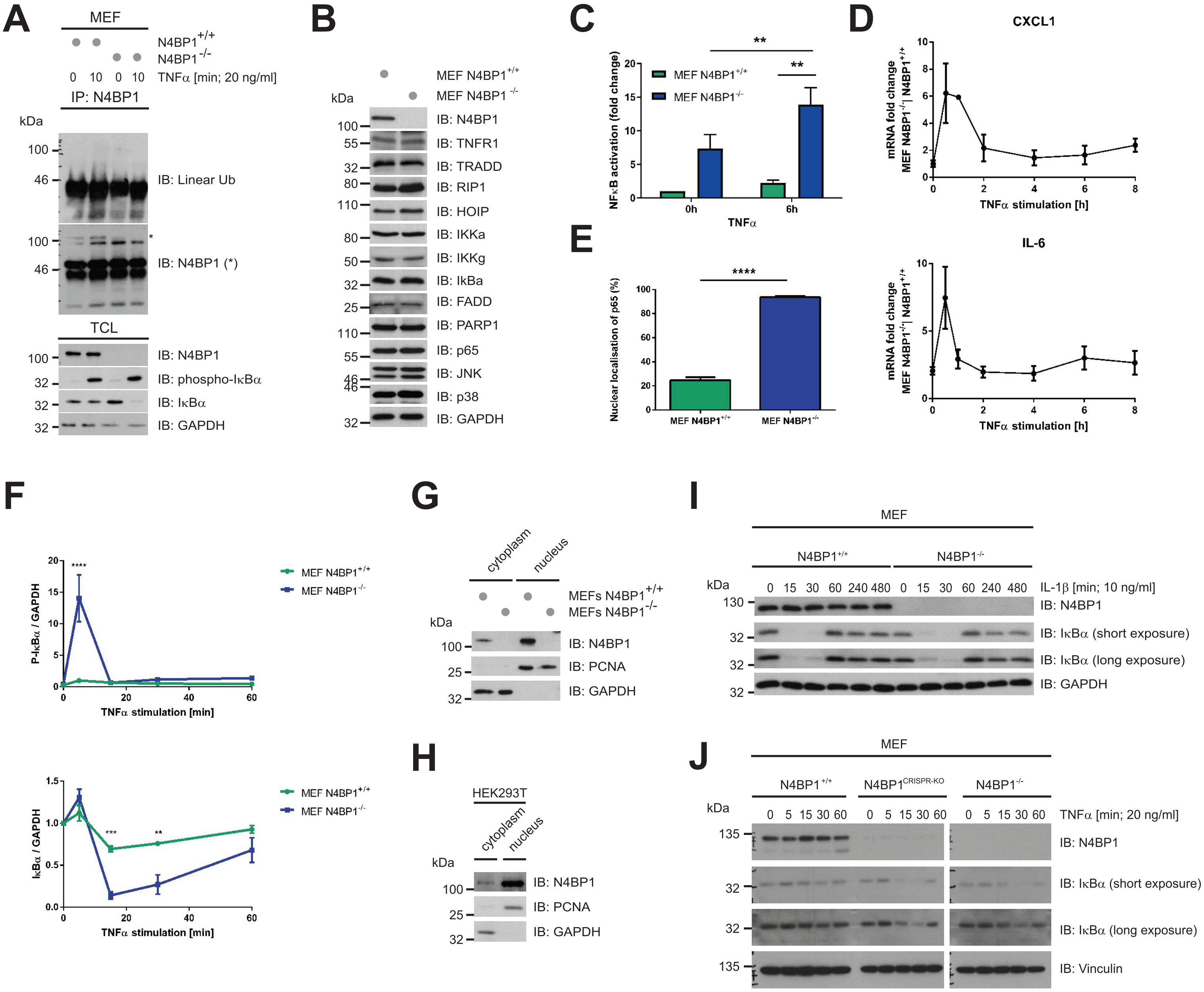

Fig. S2 N4BP1 inhibits TNFα-stimulated NFκB signalling. A Binding of M1-linked HMW Ub species to N4BP1. Immunoprecipitated endogenous N4BP1 and total cell lysates upon 10 min TNFα (20 ng/ml) treatment were analysed by Western blotting with indicated antibodies. **B** Protein levels of N4BP1 and various components of TNFR1-SC and complex II in N4BP1+/+ and N4BP1-/- MEFs. **C** Effect of N4BP1 on NFκB transcriptional activity. N4BP1+/+ and N4BP1-/- MEFs were transiently transfected with pNFκB-Luc and pUT651 plasmids encoding luciferase and β-galactosidase, respectively. After 24 hours, cells were starved for 16 hours, followed by 6 hours stimulation with TNFα (20 ng/ml). Lysates were subjected to luciferase and β-galactosidase assays. Results are shown as means and s.e.m. (n=5). ** P < 0.01, * P < 0.05, determined by two-way ANOVA test. **D** Effect of N4BP1 on TNFα-induced gene expression. N4BP1+/+ and N4BP1-/- MEFs were starved for 16 hours, followed by TNFα stimulation (20 ng/ml) for indicated time periods. mRNA levels of TNFα target genes CXCL1 (left panel) and IL-6 (right panel) were determined by real-time PCR. Results are shown as means and s.e.m. (n=3). **E** Quantification of p65 localization in TNFα-stimulated N4BP1+/+ and N4BP1-/- MEFs from Fig. 3B. Three independent experimental replicates consisting of technical duplicates were performed. At least 200 cells were quantified per condition. Results are shown as means and s.e.m. (n=3). **** P < 0.0001, determined by two-tailed Student's t-test. **F** Quantification of phosphorylation (left panel) and degradation (right panel) of IκBα in N4BP1+/+ and N4BP1-/- MEFs for Fig 3C. Quantification was performed with the use of ImageJ software. Results are shown as means and s.e.m. (n=3). n.s., no statistically significant difference, P > 0.05, * P < 0.05, *** P < 0.001, **** P < 0.0001, determined by two-way ANOVA, post hoc Sidak's multiple comparisons test. (G-H) Western blot analysis of cellular localization of endogenous N4BP1 in MEFs (**G**) and HEK293T cells (**H**). After cellular fractionation of N4BP1+/+MEFs, N4BP1-/- MEFs and HEK293T cells, localization of N4BP1 was determined by using anti-N4BP1 antibody, respectively. PCNA and GAPDH were used as markers of nuclear and cytoplasmic fractions, respectively. **I** Effect of N4BP1 on NFκB signalling pathway upon IL-1β stimulation. After 16 hours starvation, N4BP1+/+ and N4BP1-/- MEFs were treated with IL-1β (10 ng/ml) for indicated time periods, resolved by SDS-PAGE and analysed by Western blot with indicated antibodies. **J** Effect of N4BP1 on NFκB signalling pathway upon TNFα stimulation. After 16 hours starvation, N4BP1+/+, N4BP1CRISPR-KO and N4BP1-/- MEFs were treated with TNFα (20 ng/ml) for indicated time periods, resolved by SDS-PAGE and analysed by Western blot with indicated antibodies.

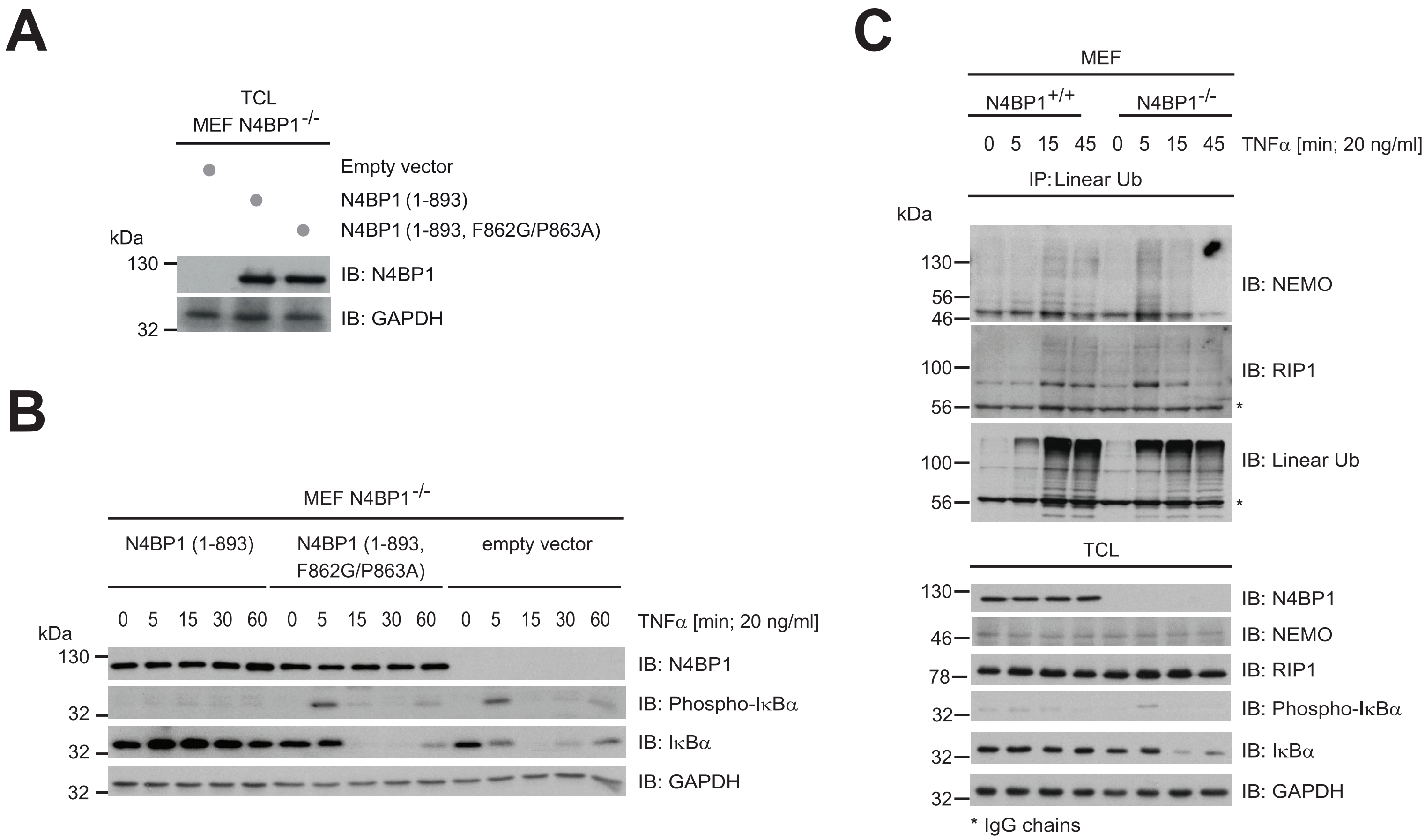

Fig. S3 N4BP1 negatively regulates TNFR1 signalling through linear ubiquitin binding. A Immunoblot showing reconstitution of N4BP1-/- MEFs with empty vector, HA-N4BP1 (1-893) or HA-N4BP1 (1-893, F862G/P863A). **B** Determination of the effect of linear Ub binding-deficient N4BP1 on phosphorylation and degradation of IκBα. After 16 hours of serum starvation, N4BP1-/- MEFs reconstituted with empty vector, HA-N4BP1 (1-893) or HA-N4BP1 (1-893, F862G/P863A) were treated with TNFα (20 ng/ml) for indicated time periods. **C** The effect of N4BP1 on the kinetics of linear polyUb chain assembly by LUBAC. Western blot analysis of immunoprecipitated linear Ub HMW species and total cell lysates from TNFα-treated (20 ng/ml) N4BP1+/+ and N4BP1-/- MEFs for the indicated time periods.

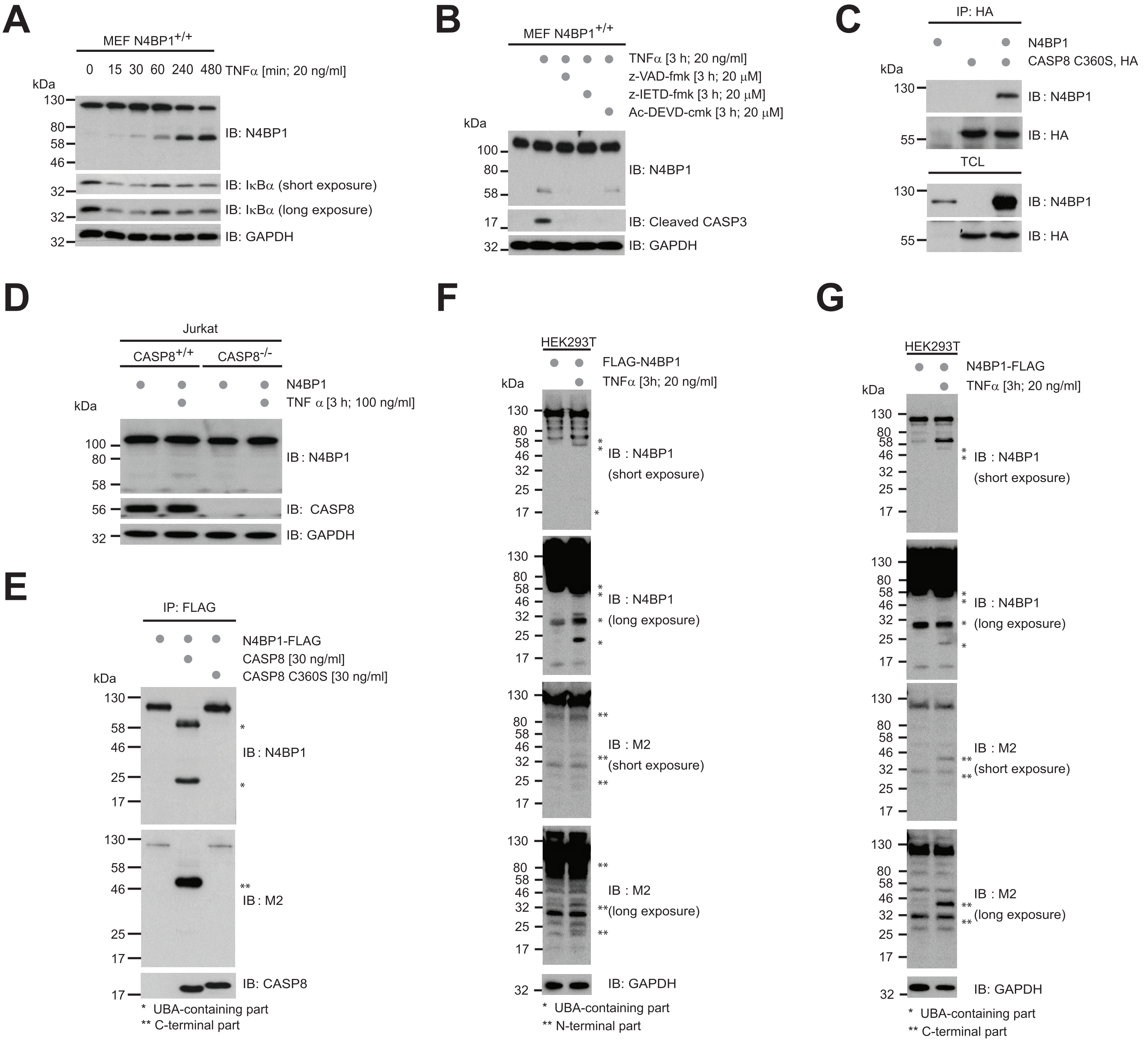

Fig. S4 N4BP1 is cleaved by CASP8. A Proteolytic cleavage of endogenous N4BP1 upon prolonged TNFα stimulation. N4BP1+/+ and N4BP1-/- MEFs were serum starved for 16 hours, followed by TNF stimulation (20 ng/ml) for indicated time points. **B** Protease cleavage of N4BP1 upon TNFα stimulation. N4BP1+/+ MEFs were pre-treated with general CASP inhibitor (zVAD-fmk, 20 μM), CASP8- (z-IETD-fmk, 20 μM) or CASP3-specific (Ac-DEVD-cmk, 20 μM) inhibitors for 1 hour, followed by TNFα stimulation (20 ng/ml) for 3 hours. **C** Analysis of N4BP1 interaction with catalytic-dead CASP8 C360S. HEK293T cells were transiently transfected with indicated plasmids. Twenty-four hours later, immunoprecipitation of HA-CASP8 C360S was performed, followed by Western blotting with indicated antibodies. **D** Comparison of N4BP1 proteolytic processing in TNFα-stimulated CASP8+/+ and CASP8-/- Jurkat cells. CASP8+/+ Jurkat and CASP8-/- Jurkat cells were transfected with N4BP1 by electroporation. Twenty-four hours later, cells were treated with TNFα (100 ng/ml) for 3 hours. **E** In vitro cleavage of N4BP1-FLAG by recombinant CASP8. N4BP1-FLAG was immunoprecipitated under denaturing conditions and eluted with 3xFLAG peptide. Eluate was divided into 3 equal aliquots and each aliquot was incubated for 3 hours either with recombinant active CASP8, catalytic-dead CASP8 (CASP8 C360S) or kept as input. **F** Western blotting showing TNFα-dependent processing of FLAG-N4BP1. HEK293T cells were transiently transfected with FLAG-N4BP1. Twenty-four hours later, cells were starved for 16 hours, followed by TNFα (20 ng/ml) treatment for 6 hours. **G** Immunoblot representing TNFα-dependent cleavage of N4BP1-FLAG. HEK293T cells were transiently transfected with N4BP1-FLAG. Samples were prepared as in (F).

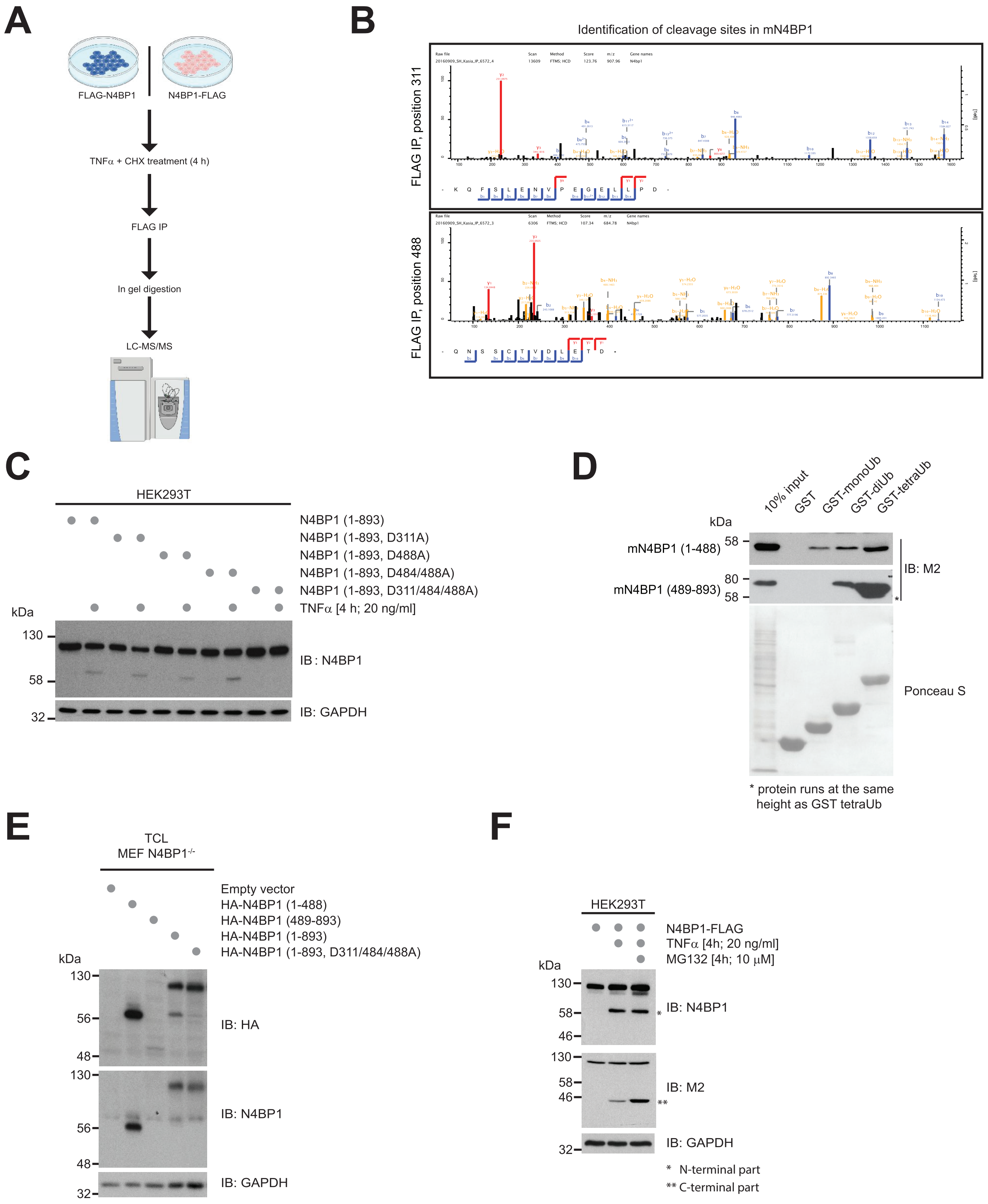

Fig. S5 CASP8 cleaves N4BP1 at multiple positions. A Schematic representation of the MS approach for identification of CASP8 cleavage sites in N4BP1. HEK293T cells transiently expressing either FLAG-N4BP1 or N4BP1-FLAG were treated with TNFα (10 ng/ml) and CHX (0.5 μg/ml) for 4 hours. Samples were resolved by SDS-PAGE and further processed for MS analysis. **B** MS/MS spectra showing identified N4BP1 297QFSLENVPEGELLPD311 (top panel) and 477QNSSCTVDLETD488 (lower panel) peptides obtained upon TNFα +CHX treatment. **C** Western blot analysis of N4BP1-FLAG and its single, double and triple cleavage site mutants in HEK293T upon TNFα stimulation (20 ng/ml, 3 h). Twenty-four hours post transfection, HEK293T cells expressing either N4BP1-FLAG (1-893), N4BP1-FLAG (1-893, D311A), N4BP1-FLAG (1-893, D488A), N4BP1-FLAG (1-893, D484/488A) or N4BP1-FLAG (1-893, D311/484/488A) were serum starved for 16 hours, followed by TNFα stimulation (20 ng/ml) for 4 hours. **D** GST pull-down assay of two major N4BP1 cleavage fragments transiently overexpressed in HEK293T cells with recombinant GST fusions of mono-, di- and tetraUb. GST alone was used as negative binding control. **E** Reconstitution of N4BP1-/- MEFs with empty vector, HA-N4BP1 (1-488), HA-N4BP1 (489-893), HA-N4BP1 (1-893) and HA-N4BP1 (1-893, D311/484/488A). **F** Stabilization of C-terminal cleavage fragment of N4BP1 upon inhibition of 26S proteasome. HEK293T cells were transiently transfected with N4BP1-FLAG. Twenty-four hours later, cells were starved for 16 hours, followed by TNFα (20 ng/ml) and MG132 (10 μM) treatment for 4 hours.

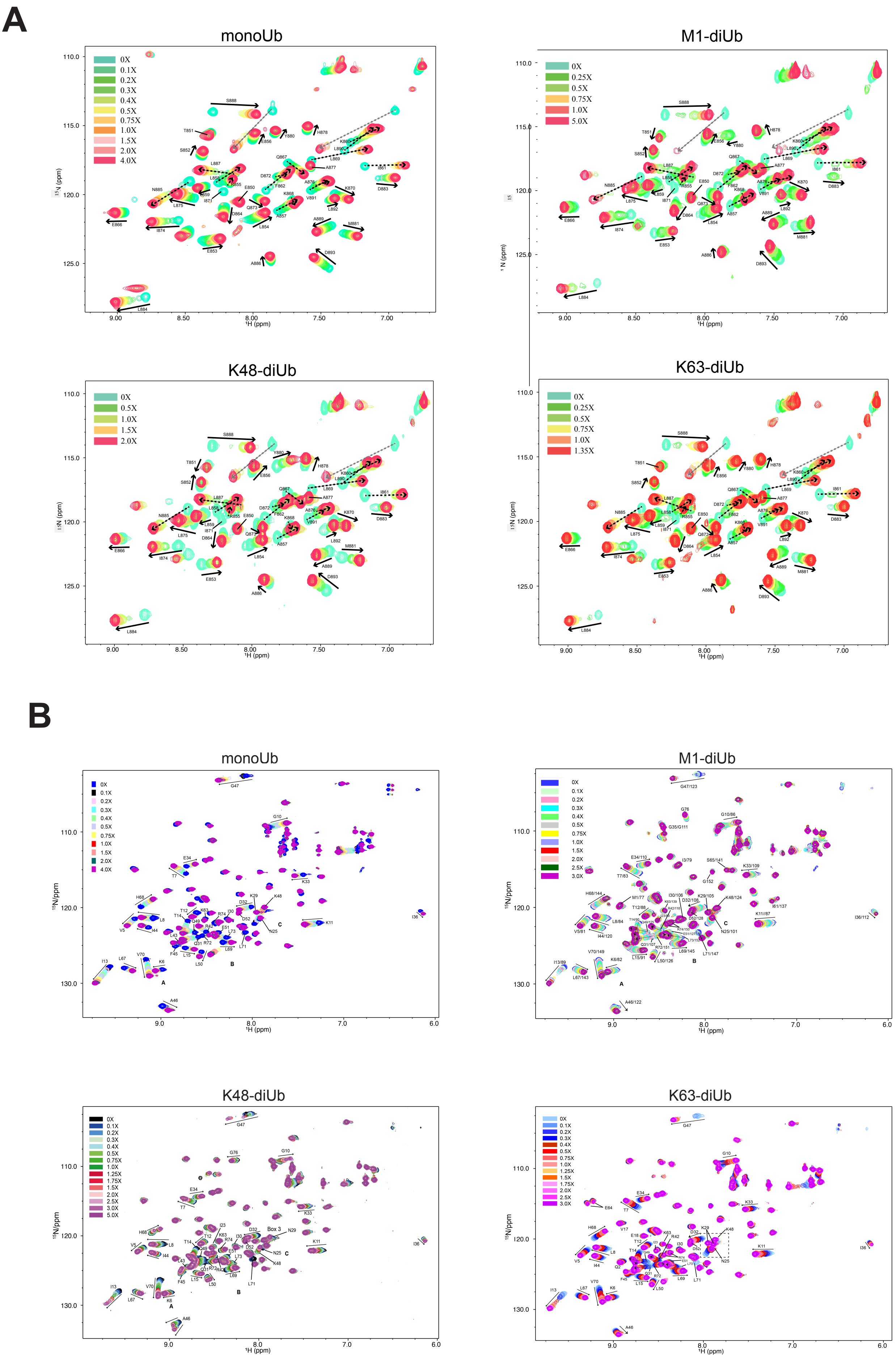

Fig. S6 NMR titrations of 15N-labelled binding partners of complexes between ubiquitin linkages and the CUE domain of N4BP1. A Overlay of 1H-15N-HSQC spectra of the N4BP1 (850-893) with increasing amounts of either monoUb, M1-diUb, K63-diUb or K48-diUb. Peaks are coloured according to the Ub:N4BP1 ratio of the titration. **B** Overlay of 1H-15N-HSQC spectra of monoUb, M1-diUb, K63-diUb and K48-diUb with increasing amounts of the N4BP1 (850-893). Peaks are coloured according to the Ub:N4BP1 ratio of the titration.

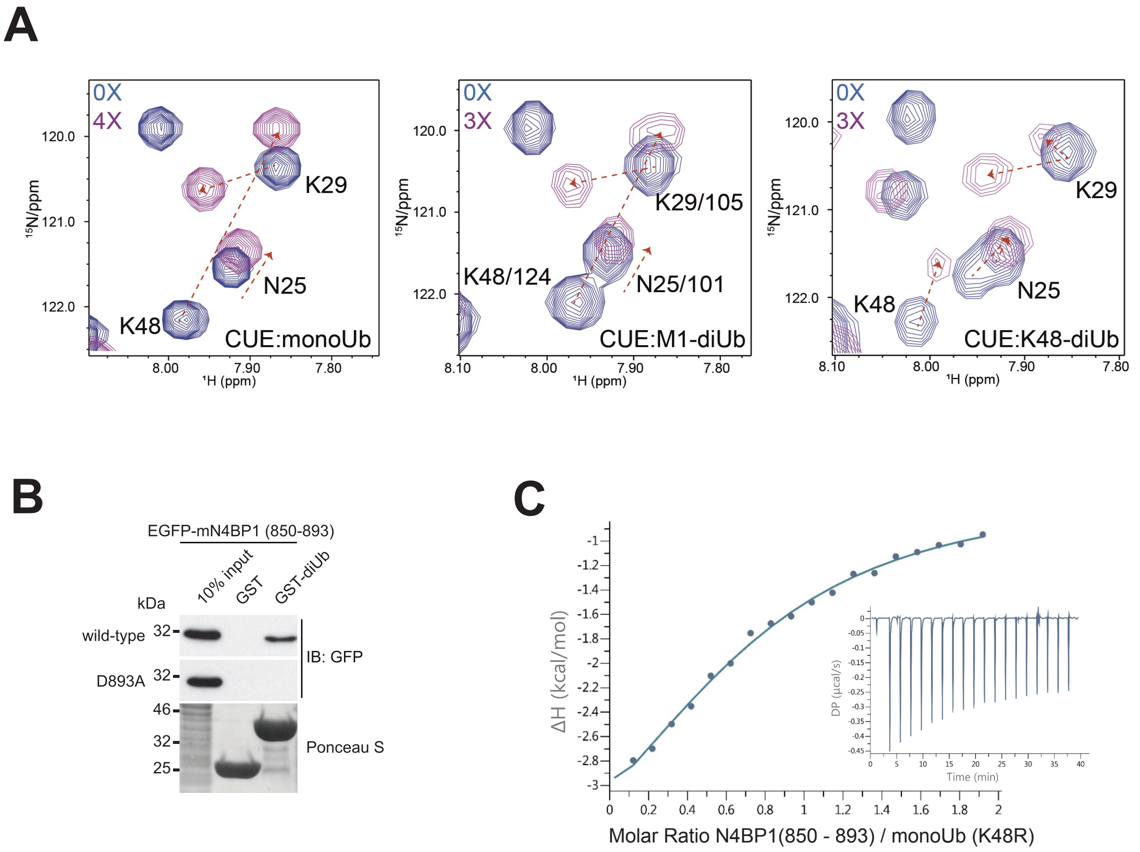

Fig. S7 Specific recognition of K48 in ubiquitin by N4BP1 discriminates against K48 linked ubiquitin chain interaction. A Overlay of an expanded region from the 1H-15N-HSQC spectra of monoUb, M1-diUb and K48-diUb showing amide proton and nitrogen shifts of N25, K29 and K48 in the absence and presence of N4BP1 (850-893). **B** GST pull-down assay of mouse N4BP1 CUE domain (850-893) transiently overexpressed as EGFP fusion in HEK293T cells with GST fusions of mono- and diUb. The mutation D893A diminishes Ub binding by disrupting the polar interaction between N4BP1 and Ub. **C** ITC analysis of N4BP1 CUE with Ub K48R shows a similar binding affinity as Ub wt.

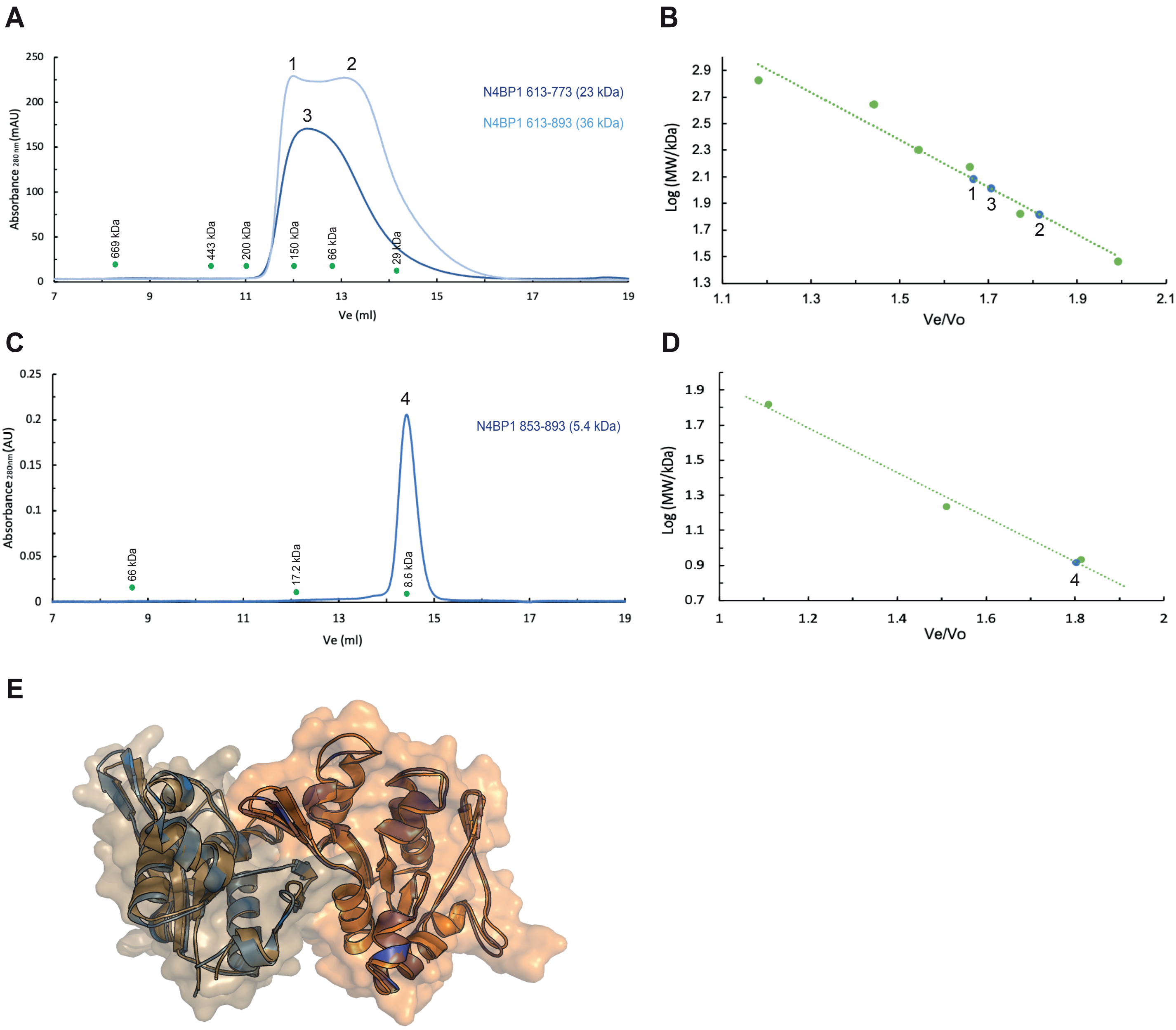

Fig. S8 The RNase domain of N4BP1 induces dimerization. A Overlay of elution profiles of N4BP1 (613-774) and N4BP1 (613-893) samples loaded on an analytical Superose 12 column. Green dots correspond to elution volumes of molecular weight standards, which are plotted against the standardised elution volume Ve/Vo (**B**). The molecular weights of elution peaks 1 and 2 of N4BP1 (613-893) and peak 3 of N4BP1 (613-774) are indicated as blue dots and correspond to 121 kDa and 65 kDa for peak 1-2 which indicate dimeric higher order (tetrameric) species and 65 kDa for peak 3, indicating dimeric species. **C, D** Same analysis as in (A, B) for N4BP1 (850-893). The sample was loaded on an analytical Superdex75 column. The elution peak 4 corresponds to an apparent molecular weight of 8.2 kDa which is smaller than the theoretical CUE domain dimer. **E** Homology model of the RNase domain of N4BP1 in cartoon and surface presentation is coloured in pale and bright orange. The dimeric structure of the RNase domain of MCPIP1 (PDB ID: 5H9W) was used as a template (shown in pale and bright blue).

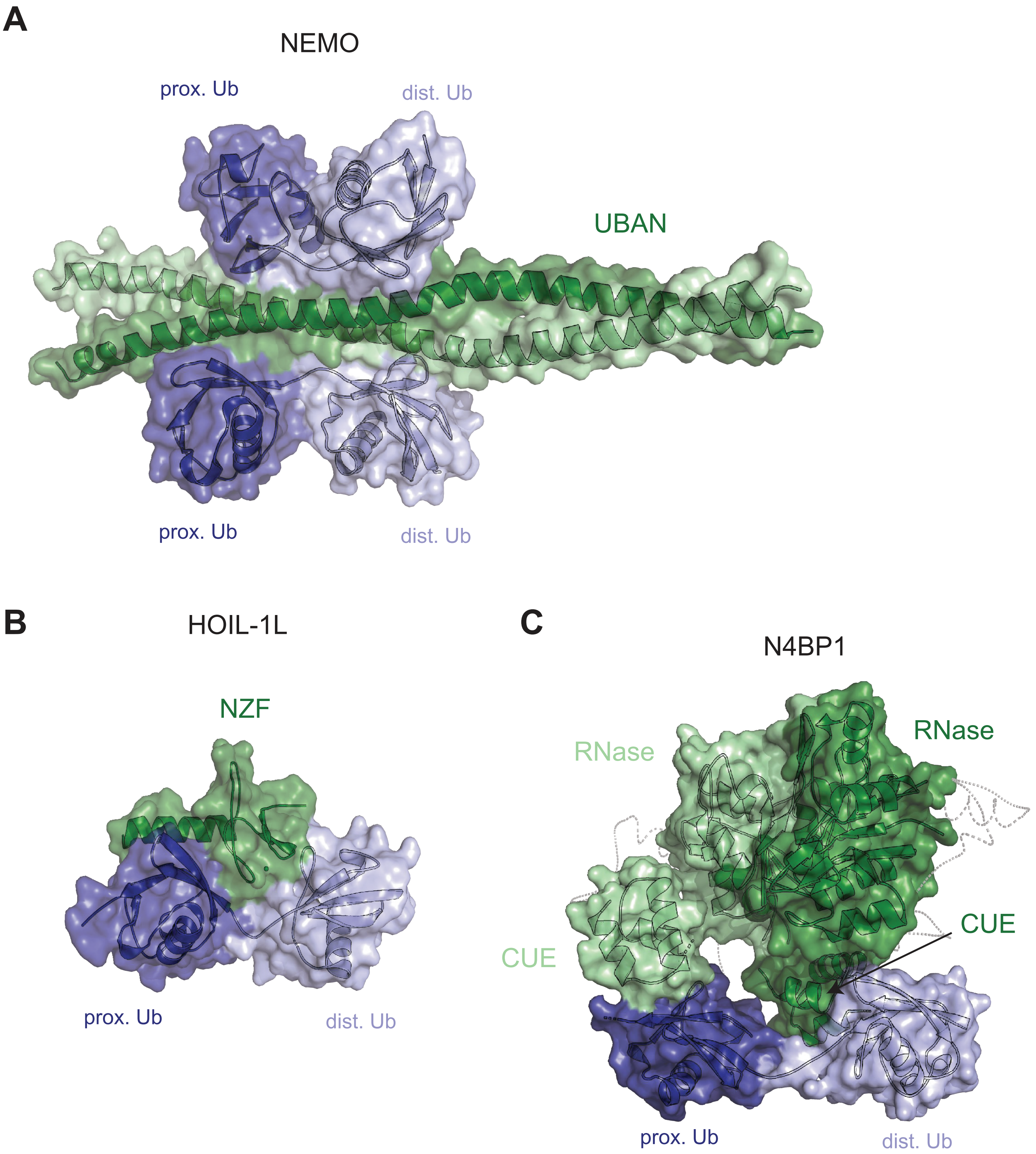

Fig. S9 Comparison of different LUBID interaction modes. A The UBAN domain of NEMO forms a parallel homodimer. The symmetric coiled-coil generates an interface which simultaneously interacts with two M1-linked Ub chains (PDB ID: 2ZVO). **B** The NZF of HOIL-1L and its C-terminal α-helical extension binds to the proximal and distal Ub of linear Ub chain linkages (PDB ID: 3B08). **C** Structural model of N4BP1 in complex with M1-diUb. Homodimerization of N4BP1 is facilitated by the RNase domains, which enables the recognition of the proximal and distal Ub of M1-linked diUb by two CUE domains.

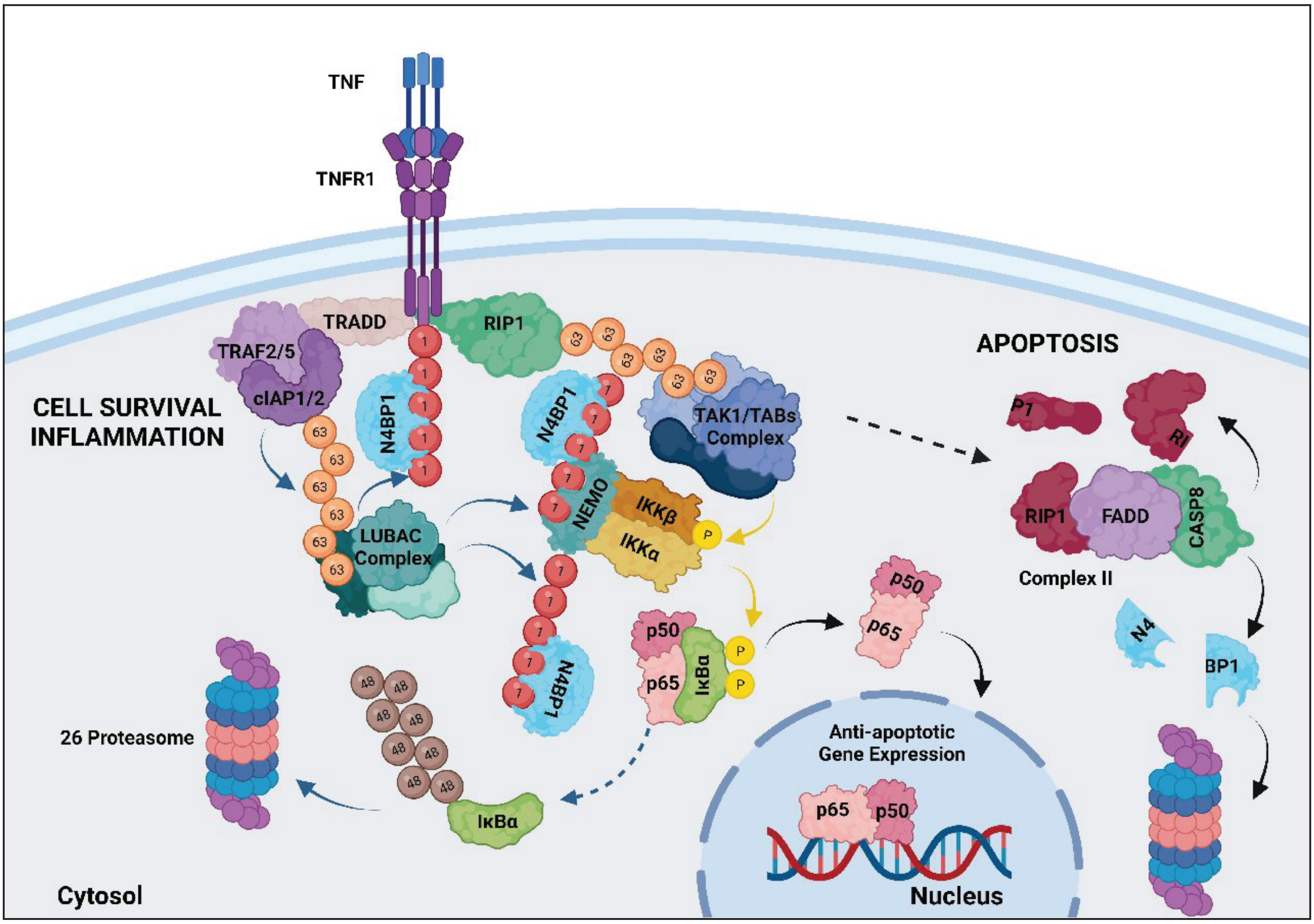

Fig. S10 Regulation of TNFR1 signalling by N4BP1. Upon binding of TNFα, transmembrane TNFR1 undergoes trimerization, which enables formation of TNFR1-SC. Initially, TNFR1 trimer independently binds TRADD and RIP1. TRADD functions as a scaffold protein, which recruits Ub ligases cIAP1 and cIAP2 through TRAF2/5 to TNFR1-SC. cIAP1/2 attaches K63-linked Ub chains on RIP1 and modifies itself, which is a prerequisite for LUBAC recruitment. Once recruited, LUBAC assembles M1 linkages on TNFR1-SC components, which are recognized by adapter subunit NEMO of IKK complex. Subsequently, LUBAC conjugates M1 linkages on NEMO, which is essential for the IKK activation. Next, IKK-mediated phosphorylation of NFκB inhibitor IκBα leads to its K48 ubiquitination by SCF$^{\beta\text{-TrCP}}$. Subsequent 26S proteasome-mediated removal of IκBα enables nuclear translocation of NFκB/REL transcription factors and the induction of NFκB-dependent gene expression. We propose that N4BP1 regulates prosurvival TNFR1 signalling by recognizing linear Ub chains of nascent TNFR1-SC through its LUBIN. N4BP1 likely competes with linear Ub-specific DUBs (CYLD and OTULIN) for binding to M1 linkages, similarly to other linear Ub readers. During apoptosis, active CASP8 recognizes and cleaves N4BP1. N4BP1 (489-893) cleavage fragment, which contains LUBIN, undergoes proteasomal degradation. Consequently, the removal of C-terminal N4BP1 cleavage fragment promotes cell death.

Table S1 List of plasmids.

Plasmid	Gene	Species	Source
pUT651	*LacZ*	E. coli	Ivan Dikic
CASP8, pcDNA3, C360S, HA	*CASP8*	Human	Adrian Ting
CASP8, pDuet1, 6His	*CASP8*	Human	This study
CASP8, pDuet1, C360S, 6His	*CASP8*	Human	This study
pSG5 Large T	*Large T antigen*	Simian virus	Addgene #9053
MCPIP1 (112-132)+N4BP1 (613-774) pCold-I	*MCPIP1*	Human	This study
	N4bp1	Mouse	This study
MCPIP1 (112-132)+N4BP1 (613-893) pCold-I	*MCPIP1*	Human	This study
	N4bp1	Mouse	This study
N4BP1 pcDNA3.1	*N4bp1*	Mouse	Michael Kuehn
N4BP1 pcDNA3.1, D311A	*N4bp1*	Mouse	This study
N4BP1 pcDNA3.1, D488A	*N4bp1*	Mouse	This study
N4BP1 pcDNA3.1, D484/488A	*N4bp1*	Mouse	This study
N4BP1 pcDNA3.1, D311/484/488A	*N4bp1*	Mouse	This study
N4BP1 pcDNA3.1, F862G, P863A	*N4bp1*	Mouse	This study
N4BP1 (343-893) pcDNA3.1	*N4bp1*	Mouse	This study
N4BP1 pcDNA3.1, FLAG	*N4bp1*	Mouse	This study
N4BP1 (1-893) pFLAG-CMV1	*N4bp1*	Mouse	This study
N4BP1 (1-849) pFLAG-CMV1	*N4bp1*	Mouse	This study
N4BP1 (1-771) pFLAG-CMV1	*N4bp1*	Mouse	This study
N4BP1 (144-893) pFLAG-CMV1	*N4bp1*	Mouse	This study
N4BP1 (393-893) pFLAG-CMV1	*N4bp1*	Mouse	This study
N4BP1 (393-849) pFLAG-CMV1	*N4bp1*	Mouse	This study
N4BP1 (393-771) pFLAG-CMV1	*N4bp1*	Mouse	This study
N4BP1 (1-392) pFLAG-CMV1	*N4bp1*	Mouse	This study
N4BP1 (1-143) pFLAG-CMV1	*N4bp1*	Mouse	This study
N4BP1 (144-392) pFLAG-CMV1	*N4bp1*	Mouse	This study
N4BP1 (1-488), NpFLAG-CMV1	*N4bp1*	Mouse	This study
N4BP1 (489-893), NpFLAG-CMV1	*N4bp1*	Mouse	This study
N4BP1 (613-893), NpFLAG-CMV1	*N4bp1*	Mouse	This study
N4BP1 (613-849), NpFLAG-CMV1	*N4bp1*	Mouse	This study
N4BP1 (613-773), NpFLAG-CMV1	*N4bp1*	Mouse	This study
N4BP1 (312-392) pEGFP-C1	*N4bp1*	Mouse	This study
N4BP1 (312-342) pEGFP-C1	*N4bp1*	Mouse	This study

N4BP1 (703-896) pEGFP-C1	*N4BP1*	Human	This study
N4BP1 (850-893) pEGFP-C1	*N4bp1*	Mouse	This study
N4BP1 (850-893) pEGFP-C1, F862G, P863A	*N4bp1*	Mouse	This study
N4BP1 (850-893) pEGFP-C1, D893A	*N4bp1*	Mouse	This study
N4BP1 (1-893), pcDNA3, HA	*N4bp1*	Mouse	This study
N4BP1 (1-893), pBabe-puro, HA	*N4bp1*	Mouse	This study
N4BP1 (1-893), D311A, D484A, D488A, pBabe-puro, HA	*N4bp1*	Mouse	This study
N4BP1 (1-488), pBabe-zeo, HA	*N4bp1*	Mouse	This study
N4BP1 (489-893), pBabe-puro, HA	*N4bp1*	Mouse	This study
N4BP1 (850-893) pGEX-4T1	*N4bp1*	Mouse	This study
N4BP1 (706-893) pGEX-4T1	*N4bp1*	Mouse	This study
N4BP1 (343-893) pGEX-4T1	*N4bp1*	Mouse	This study
N4BP1 (613-774) pCold-TF	*N4bp1*	Mouse	This study
N4BP1 (613-893) pCold-TF	*N4bp1*	Mouse	This study
N4BP1 (850-893), pET47	*N4bp1*	Mouse	This study
LentiCRISPRv2-sgN4BP1	*N4bp1*	Mouse	This study<br>(modified RRID:Addgene_52961)
hTNF (77-233)-pRSET-His-PP, STREP	*TNFα*	Human	This study
Ub, pGEX-4T1	*UBB*	Human	This study
Ub, pGEX-6P1	*UBB*	Human	This study
Ub, pGEX-6P1, K48A	*UBB*	Human	This study
Ub, pGEX-6P1, K48R	*UBB*	Human	This study
diUb, pET47	*UBB*	Human	This study
diUb-pGEX-4T1	*UBB*	Human	This study
diUb-pGEX-6P1	*UBB*	Human	This study
tetraUb-pGEX-4T2	*UBB*	Human	Errol Friedberg, Caixia Guo
hexaUb (GV, deltaGG) YTH9	*UBB*	Human	This study
UBE2R1, pET15b, 6His-SUMO1	*UBE2R1*	Yeast	MRC Protein Phosphorylation and Ubiquitylation Unit, DU No. 51247
UBE2D3, pGEX-6P1	*UBE2D3*	Human	This study
UBE2N, pET15b, 6His 3C, E60C	*UBC13*	Human	MRC Protein Phosphorylation and Ubiquitylation Unit, DU No. 24653
UBA1, pET28	*UBA1*	Mouse	This study
UBE2V1, pET15b, 6His 3C	*UBE2V1*	Human	MRC Protein Phosphorylation and Ubiquitylation Unit, DU No. 20496

Table S2 List of antibodies.

Protein/tag	Antibody	Source	Application
CASP3, cleaved	5A1E (9664)	Cell Signaling	WB
CASP8	D35G2 (4790)	Cell Signaling	WB
FADD	sc-6036	Santa Cruz Biotechnology	IP, WB
GAPDH	14C10 (2118)	Cell Signaling	WB
IκBα	112B2 (9247)	Cell Signaling	WB
N4BP1 (mouse-specific)	ab133610	Abcam	IP, WB
N4BP1 (human-specific)	ab169329	Abcam	WB
p65	C-20 (sc-372)	Santa Cruz Biotechnology	IF
PARP	9542	Cell Signaling	WB
Phospho-p38	9216	Cell Signaling	WB
Phospho-IκBα	5A5 (9246)	Cell Signaling	WB
Phospho-JNK	9255	Cell Signaling	WB
PCNA	PC10 (sc-56)	Santa Cruz Biotechnology	WB
RIP1	610459	BD Transduction Laboratories	IP, WB
TRADD	sc-7868	Santa Cruz Biotechnology	WB
TRAF2	4724 (C192)	Cell Signaling	WB
Ubiquitin (linear)	1F11/3F5/Y102L	Genentech	IP, WB
Ubiquitin	3933	Cell Signaling	WB
Ubiquitin	P4D1 (sc-8017)	Santa Cruz Biotechnology	WB
FLAG	M2	Sigma-Aldrich	IP, WB
GFP	sc-9996	Santa Cruz Biotechnology	WB
HA	HA.11	Covance	WB
STREP	34850	Qiagen	WB
	goat anti-mouse IgG HRP conjugate	Bio-Rad	2nd Ab
	goat anti-rabbit HRP conjugate	DAKO	2nd Ab
	donkey anti-goat HRP conjugate	Santa Cruz Biotechnology	2nd Ab
	goat anti-human HRP conjugate	Jackson ImmunoResearch	2nd Ab
	donkey anti-rabbit Cy5 conjugate	Jackson ImmunoResearch	IF

WB=Western blot, IP=immunoprecipitation, 2nd Ab=secondary antibody

Table S3 List of oligonucleotides used for RT PCR.

Oligonucleotide	Sequence (5´-3´)	Company
Gapdh, Sense	ACCACAGTCCATGCCATCAC	*Eurofins Genomics*
Gapdh, Antisense	CACCACCCTGTTGCTGTAGCC	*Eurofins Genomics*
Cxcl1, Sense	GCCTATCGCCAATGAGCTG	*Eurofins Genomics*
Cxcl1, Antisense	TGGGGACACCTTTTAGCATC	*Eurofins Genomics*
Il-6, Sense	CCGGAGAGGAGACTTCACAG	*Eurofins Genomics*
Il-6, Antisense	GGAAATTGGGGTAGGAAGGA	*Eurofins Genomics*

Table S4 Summary of the structure statistics for the N4BP1 CUE CS-Rosetta model.

Experimental restraints input for CS-Rosetta	
$^{13}C^{\alpha}$ shifts	44
$^{13}C^{\beta}$ shifts	44
$^{13}C'$ shifts	43
^{15}N shifts	40
$^{1}H^{N}$ shifts	40
$^{1}H^{\alpha}$ shifts	44
Total restraints	255
Average pairwise RMSD* (Å)	
C^{α}	0.49
Backbone atoms	0.48
Heavy atoms	0.80
All atoms	1.01
Structure quality**	
Ramachandran favoured regions (%)	100
Ramachandran Outliers (%)	0
Ramachandran distribution Z-score	
Whole	-1.43 ± 0.90
Helix	-0.69 ±0.60
Sheet	None
Loop	-1.64 ± 1.39
Favored rotamers (%)	100
Poor rotamers (%)	0
C^{β} deviations >0.25 Å	0
Bad bonds (%)	0
Bad angles (%)	0
All-atom clashscore***	1.4

* Pairwise RMSD was calculated using the structure ensemble containing 10 best refined models.
**Quality data for model 5 from the Structure ensemble. Model 5 is the overall representative, medoid model (most similar to other models in the ensemble).
***Clashscore is the number of serious steric overlaps (>0.4 Å) per 1000 atoms.
