# Supplementary material for: N4BP1 functions as a dimerization-dependent linear ubiquitin reader which regulates TNF signalling
Source: Cell Death Discov. 2024 Apr 20;10:183. doi: 10.1038/s41420-024-01913-8 (PMC11032371; doi:10.1038/s41420-024-01913-8)
Supplement: Supplementary file 3 — Original Data File [file 41420_2024_1913_MOESM3_ESM.pdf]

FIGURE 1A - SOURCE DATA

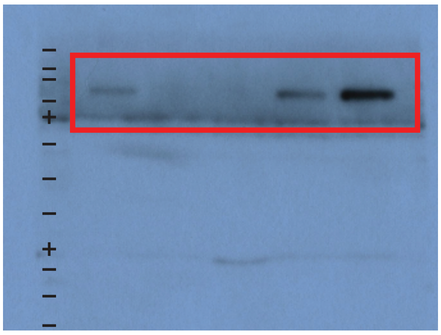

Whole gel 1
IB:N4BP1

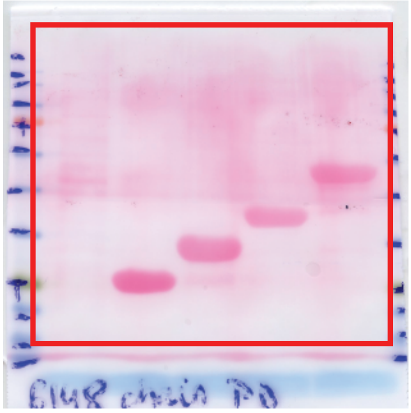

Whole gel 1
Ponceau staining

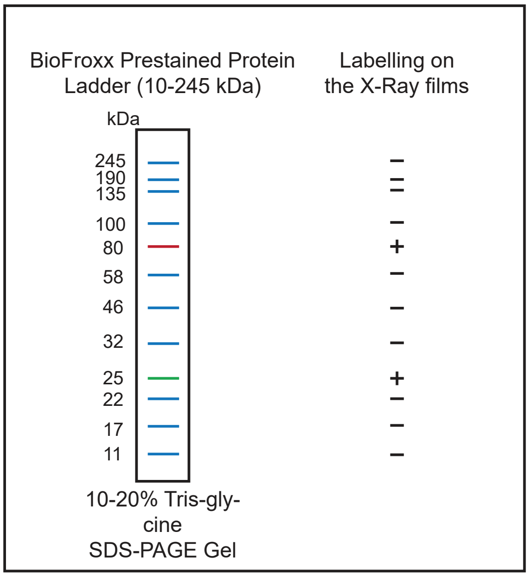

Order (marked area):

1. Input
2. GST alone
3. PD GST-monoUb
4. PD GST-diUb
5. PD GST-tetraUb

FIGURE 1C - SOURCE DATA

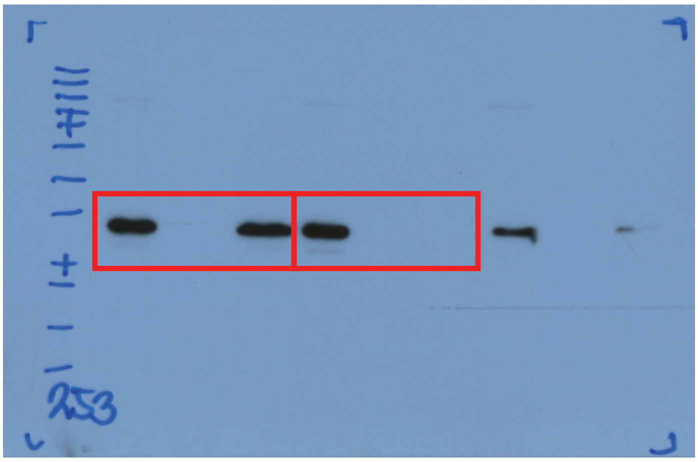

Whole gel
IB:GFP

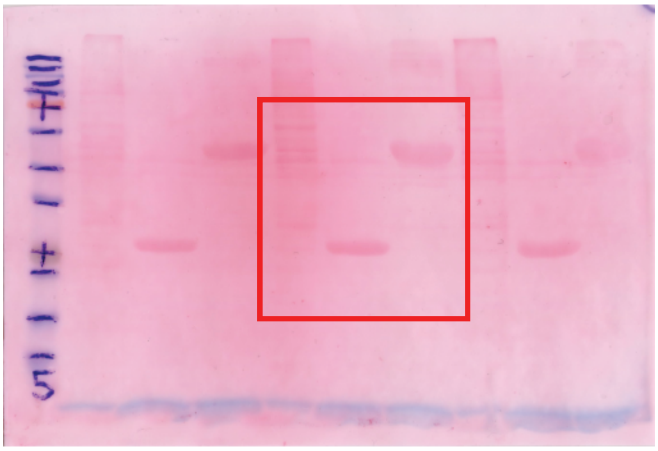

Whole gel
Ponceau staining

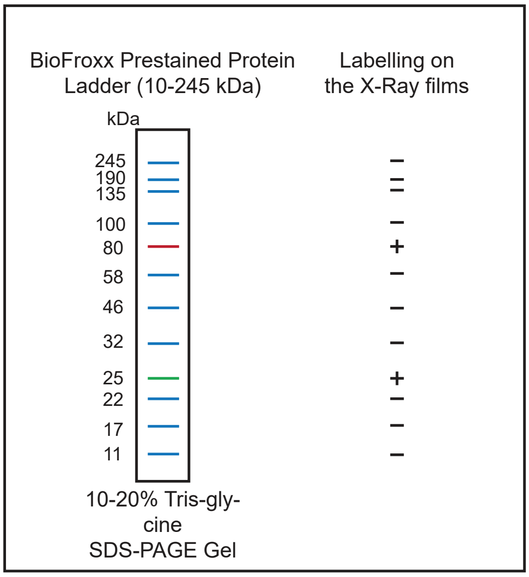

Order (marked area):

1. Input
2. GST alone
3. PD GST-tetraUb

FIGURE 1D - SOURCE DATA

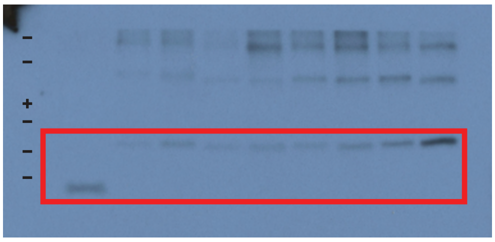

Gel 1 (Input): bottom part
IB: Ub

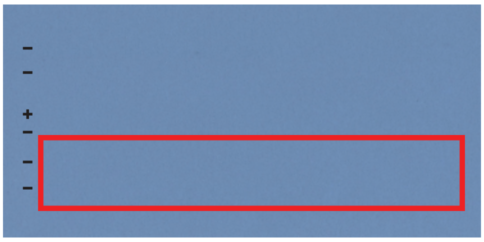

Gel 2 (PD: bottom part
IB: Ub

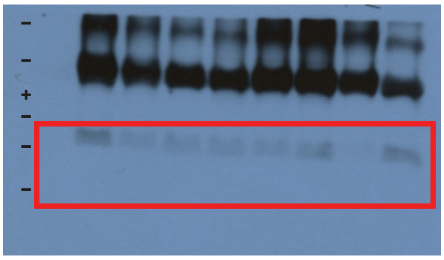

Gel 3 (PD): bottom part
IB: Ub

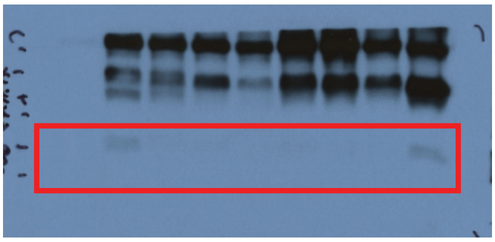

Gel 4 (PD): bottom part
IB: Ub

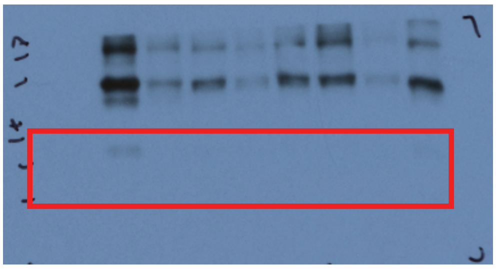

Gel 5 (PD): bottom part
IB: Ub

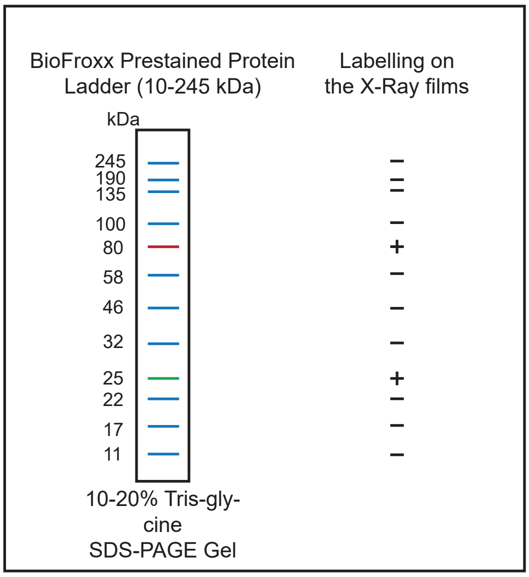

Order of gels:

1. Input (20%), IB:Ub
2. GST alone, GST PD, IB:Ub
3. mN4BP1(850-893)-GST, GST PD, IB:Ub
4. mN4BP1(706-893)-GST, GST PD, IB:Ub
5. mN4BP1(343-893)-GST, GST PD, IB:Ub

Order on each gel (left to right):

1. monoUb
2. M1-linked diUb
3. K6-linked diUb
4. K11-linked diUb
5. K27-linked diUb
6. K29-linked diUb
7. K33-linked diUb
8. K48-linked diUb
9. K63-linked diUb

FIGURE 2A - SOURCE DATA

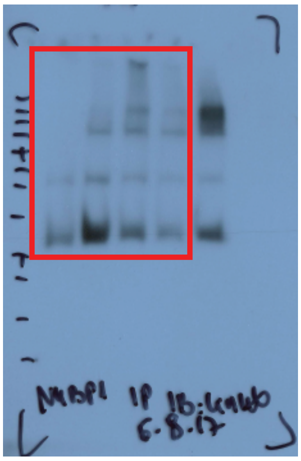

Whole gel 1 (IP)
IB: Linear Ub

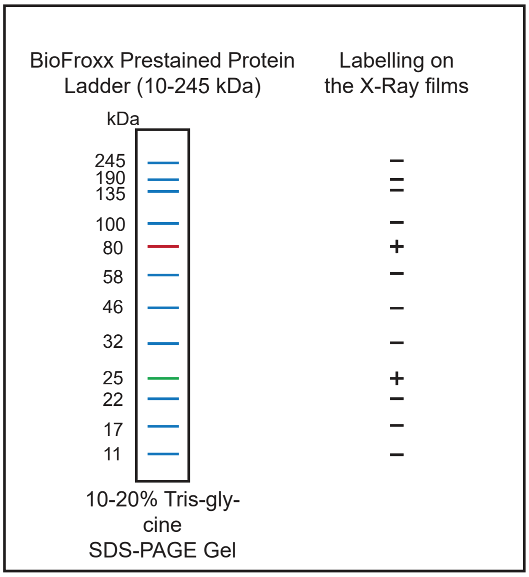

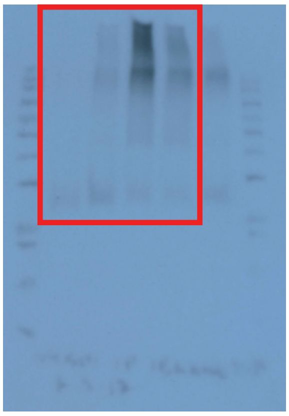

Whole gel 1 (IP)
IB: Ub (P4D1),
reprobed after stripping

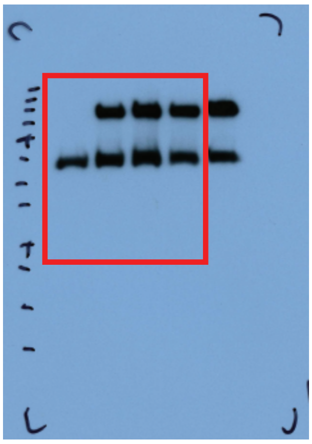

Whole gel 1 (IP)
IB: N4BP1

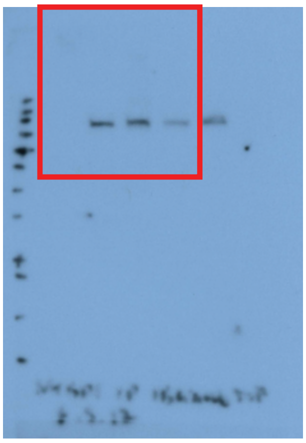

Whole gel 2 (IP)
IB: K48 polyUb

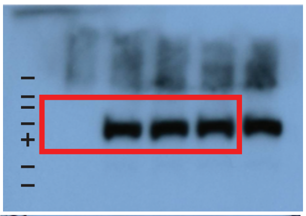

Gel 3 (TCL): top part
IB:N4BP1

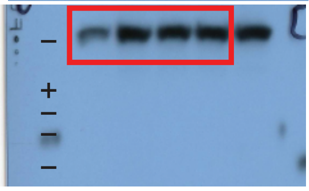

Gel 3 (TCL): bottom part
IB:GAPDH

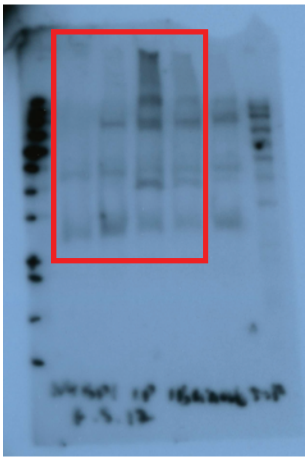

Whole gel 2 (IP)
IB: K63 poly Ub,
reprobed after stripping

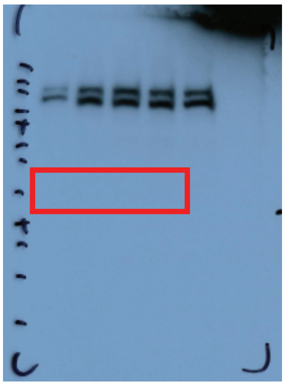

Whole gel 4 (TCL)
IB:IP-kappaBalpha

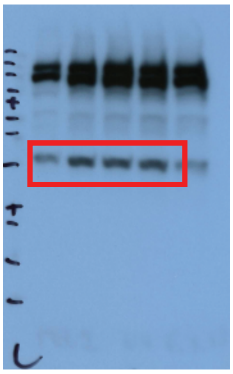

Whole gel 5 (TCL)
IB:IkappaBalpha

Order:
1. MEF N4BP1$^{-/-}$ IP N4BP1, TNF [20ng/ml; 0min]
2. MEF N4BP1$^{+/+}$ IP N4BP1, TNF [20ng/ml; 0min]
3. MEF N4BP1$^{+/+}$ IP N4BP1, TNF [20ng/ml; 5min]
4. MEF N4BP1$^{+/+}$ IP N4BP1, TNF [20ng/ml; 15min]
5. MEF N4BP1$^{+/+}$ IP N4BP1, TNF [20ng/ml; 7min]

FIGURE 2C - SOURCE DATA

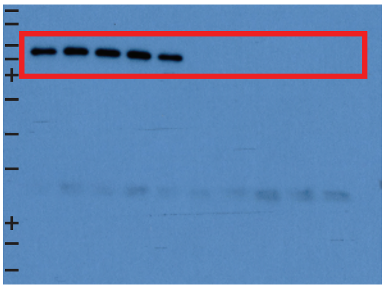

Whole gel 1 (TCL)
IB: N4BP1

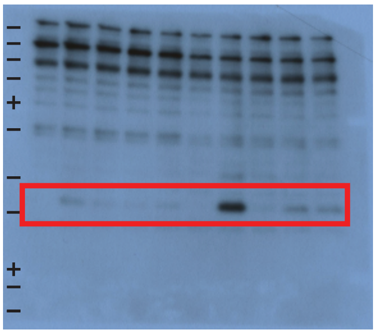

Whole gel 2 (TCL)
IB: P-IkappaBalpha

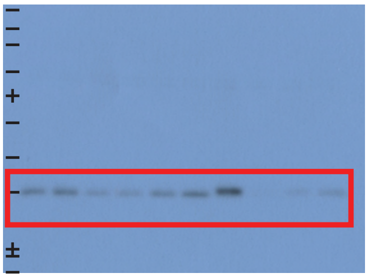

Whole gel 3 (TCL)
IB: IkappaBalpha (short exposure)

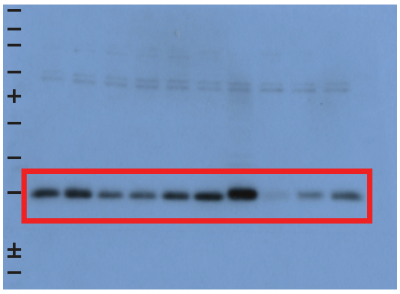

Whole gel 3 (TCL)
IB: IkappaBalpha (long exposure)

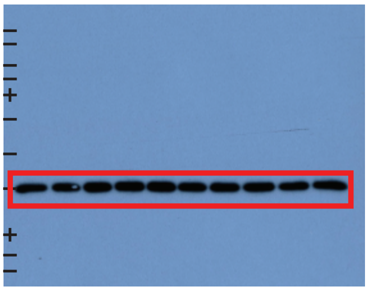

Whole gel 4 (TCL)
IB: GAPDH

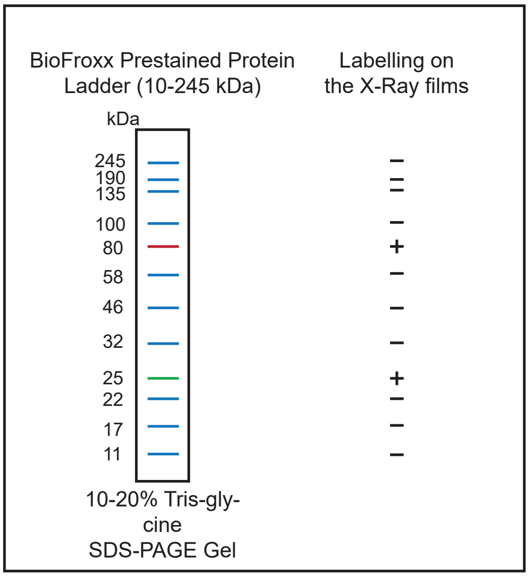

Order on each gel (left to right):

1. MEF N4BP1$^{+/+}$ TCL, TNF [20ng/ml; 0min]
2. MEF N4BP1$^{+/+}$ TCL, TNF [20ng/ml; 5min]
3. MEF N4BP1$^{+/+}$ TCL, TNF [20ng/ml; 15min]
4. MEF N4BP1$^{+/+}$ TCL, TNF [20ng/ml; 30min]
5. MEF N4BP1$^{+/+}$ TCL, TNF [20ng/ml; 60min]
6. MEF N4BP1$^{-/-}$ TCL, TNF [20ng/ml; 0min]
7. MEF N4BP1$^{-/-}$ TCL, TNF [20ng/ml; 5min]
8. MEF N4BP1$^{-/-}$ TCL, TNF [20ng/ml; 15min]
9. MEF N4BP1$^{-/-}$ TCL, TNF [20ng/ml; 30min]
10. MEF N4BP1$^{-/-}$ TCL, TNF [20ng/ml; 60min]

FIGURE 2D - SOURCE DATA

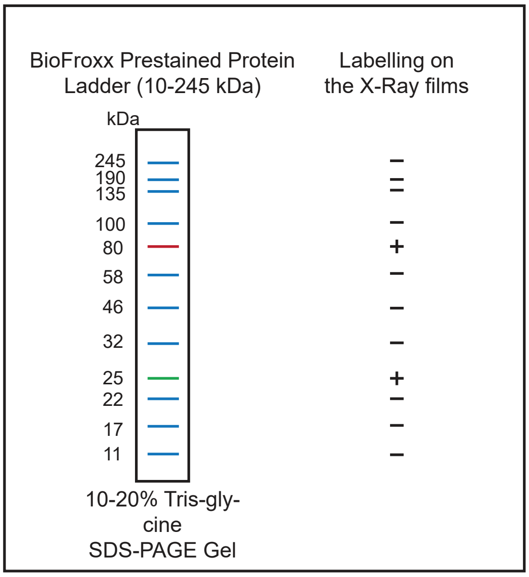

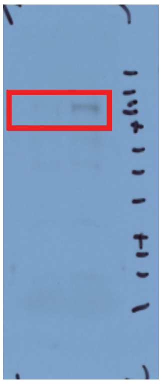

Whole gel 1 (PD)
IB: N4BP1

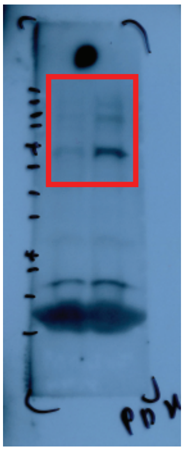

Whole gel 2 (PD)
IB: RIP1

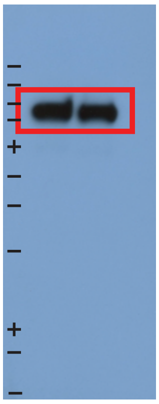

Whole gel 4 (TCL)
IB:N4BP1

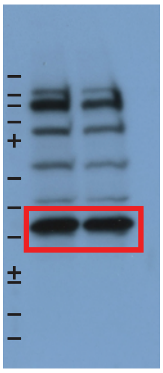

Whole gel 4 (TCL)
IB:GAPDH,
reprobed after stripping

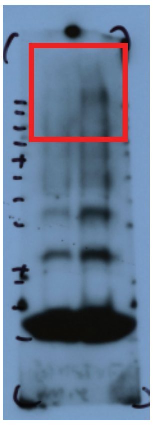

Whole gel 2 (PD)
IB: Linear Ub,
reprobed after stripping

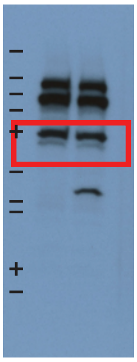

Whole gel 5 (TCL)
IB: RIP1,
reprobed after stripping

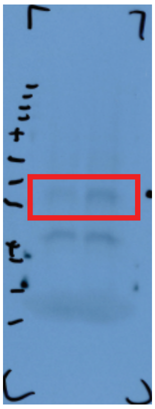

Whole gel 1 (PD)
IB: TRADD,
reprobed after stripping

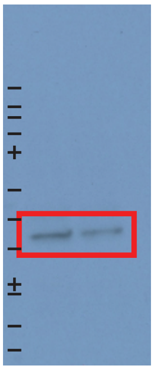

Whole gel 4 (TCL)
IB: TRADD,
reprobed after stripping

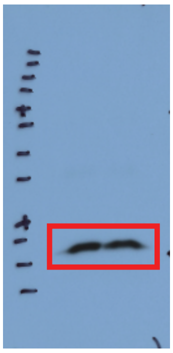

Whole gel 3 (PD)
IB: STREP

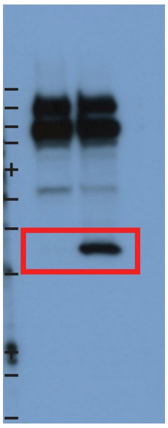

Whole gel 5 (TCL)
IB:P-IkappaBalpha

Order:
1. MEF N4BP1$^{+/+}$ PD STREP, HIS-STREP-TNF [1µg/ml; 0min]
2. MEF N4BP1$^{+/+}$ PD STREP, HIS-STREP-TNF [1µg/ml; 5min]

FIGURE 3B - SOURCE DATA

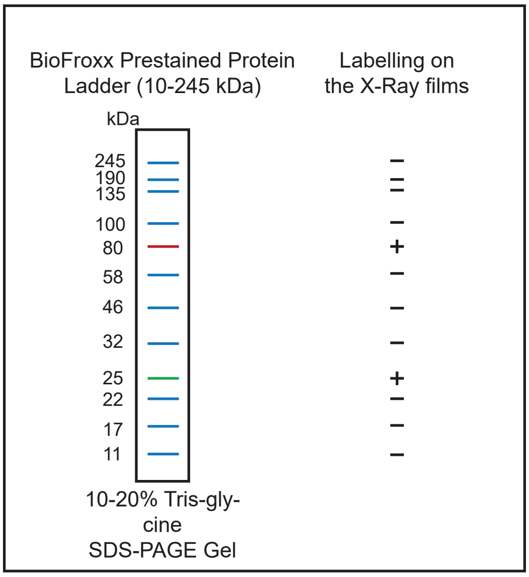

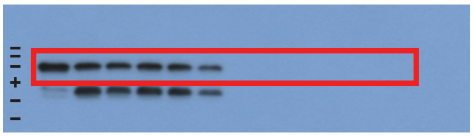

Gel 1 (TCL): top part
IB: N4BP1

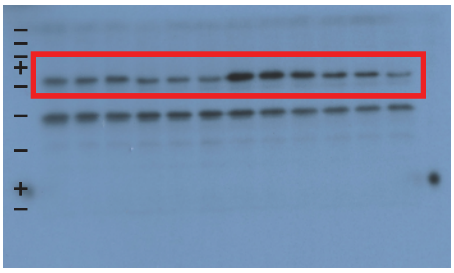

Whole gel 2 (TCL)
IB: CASP8

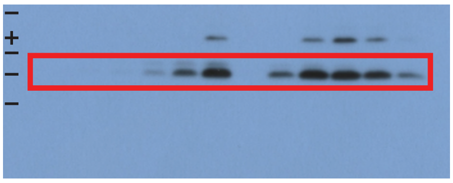

Gel 1 (TCL): bottom part
IB: Cleaved CASP3

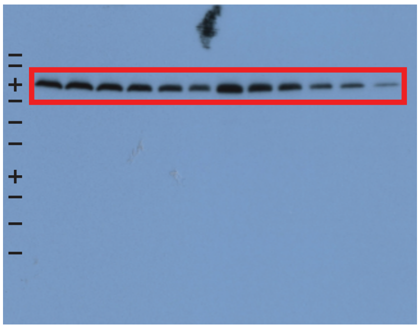

Whole gel 3 (TCL)
IB: RIP1

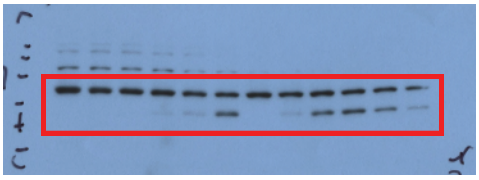

Gel 4 (TCL): top part
IB: PARP

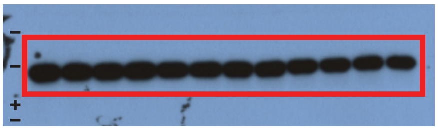

Gel 4 (TCL): bottom part
IB:GAPDH

Order:

1. MEF N4BP1$^{+/+}$ TCL, TNF + CHX [0h]
2. MEF N4BP1$^{+/+}$ TCL, TNF + CHX [2h]
3. MEF N4BP1$^{+/+}$ TCL, TNF + CHX [4h]
4. MEF N4BP1$^{+/+}$ TCL, TNF + CHX [6h]
5. MEF N4BP1$^{+/+}$ TCL, TNF + CHX [8h]
6. MEF N4BP1$^{+/+}$ TCL, TNF + CHX [12h]
7. MEF N4BP1$^{-/-}$ TCL, TNF + CHX [0h]
8. MEF N4BP1$^{-/-}$ TCL, TNF + CHX [2h]
9. MEF N4BP1$^{-/-}$ TCL, TNF + CHX [4h]
10. MEF N4BP1$^{-/-}$ TCL, TNF + CHX [6h]
11. MEF N4BP1$^{-/-}$ TCL, TNF + CHX [8h]
12. MEF N4BP1$^{-/-}$ TCL, TNF + CHX [12h]

FIGURE 3D - SOURCE DATA

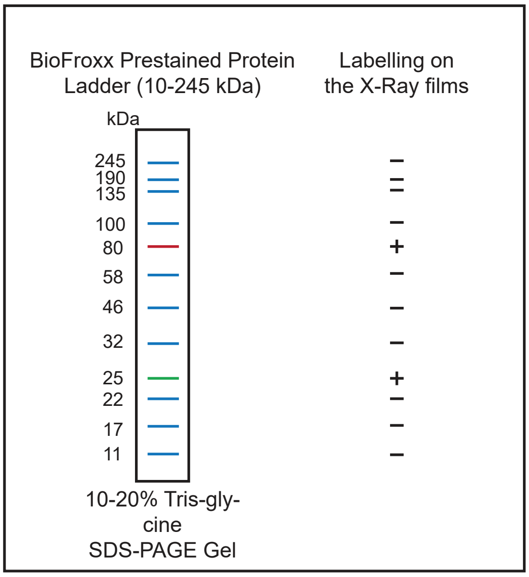

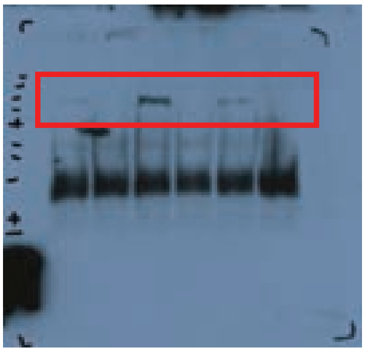

Whole gel 1 (IP)
IB: N4BP1,
reprobed after stripping

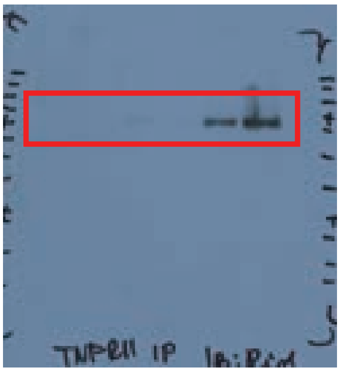

Whole gel 1 (IP)
IB: RIP1

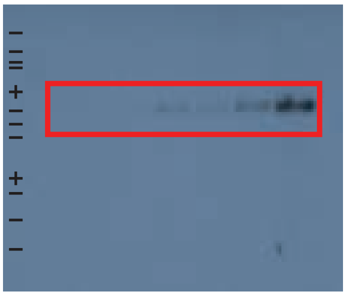

Whole gel 2 (IP)
IB: CASP8

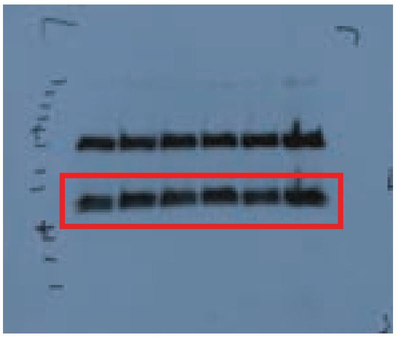

Whole gel 2 (IP)
IB: FADD,
reprobed after stripping

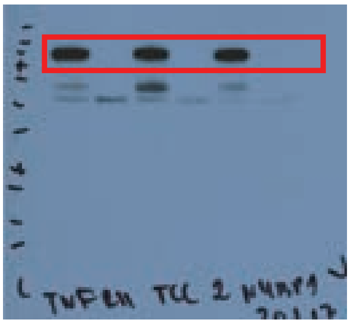

Whole gel 1 (TCL)
IB: N4BP1,
reprobed after stripping (1)

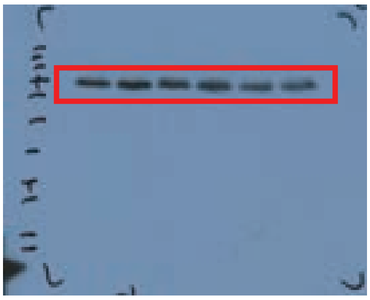

Whole gel 2 (TCL)
IB: RIP1

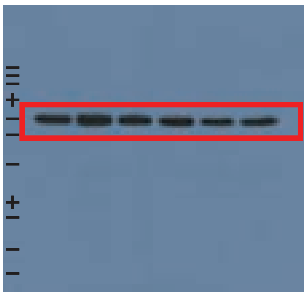

Whole gel 1 (TCL)
IB: CASP8

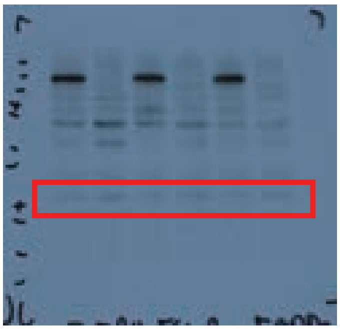

Whole gel 1 (TCL)
IB: FADD,
reprobed after stripping (2)

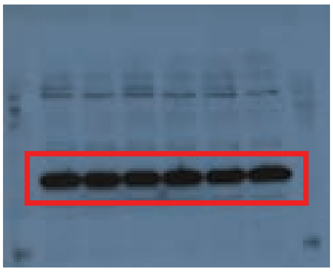

Whole gel 2 (TCL)
IB: GAPDH,
reprobed after stripping

Order:
1. MEF N4BP1$^{+/+}$ IP FADD
2. MEF N4BP1$^{-/-}$ IP FADD
3. MEF N4BP1$^{+/+}$ IP FADD, TNF + CHX
4. MEF N4BP1$^{-/-}$ IP FADD, TNF + CHX
5. MEF N4BP1$^{+/+}$ IP FADD, TNF + CHX + z-VAD-fmk
6. MEF N4BP1$^{-/-}$ IP FADD, TNF + CHX + z-VAD-fmk

SUPPLEMENTARY FIGURE 1A - SOURCE DATA

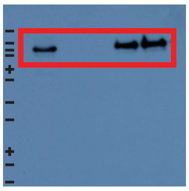

Whole gel 1 (PD)
IB: N4BP1

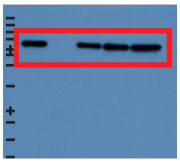

Whole gel 2 (PD)
IB: FLAG (M2)

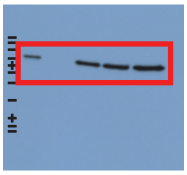

Whole gel 3 (PD)
IB: FLAG (M2)

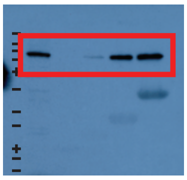

Whole gel 4 (PD)
IB: FLAG (M2)

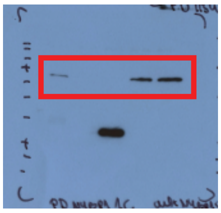

Whole gel 5 (PD)
IB: N4BP1

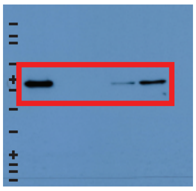

Whole gel 6 (PD)
IB: FLAG (M2)

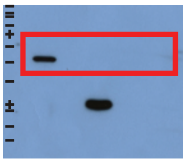

Whole gel 7 (PD)
IB: FLAG (M2)

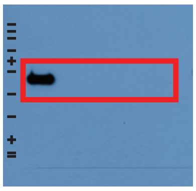

Whole gel 8 (PD)
IB: FLAG (M2)

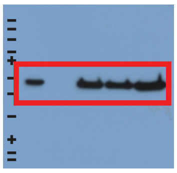

Whole gel 9 (PD)
IB: FLAG (M2)

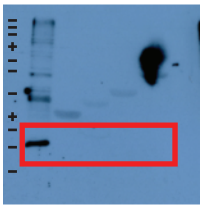

Whole gel 10 (PD)
IB: FLAG (M2)

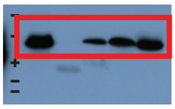

Gel 11 (PD): bottom part
IB: FLAG (M2)

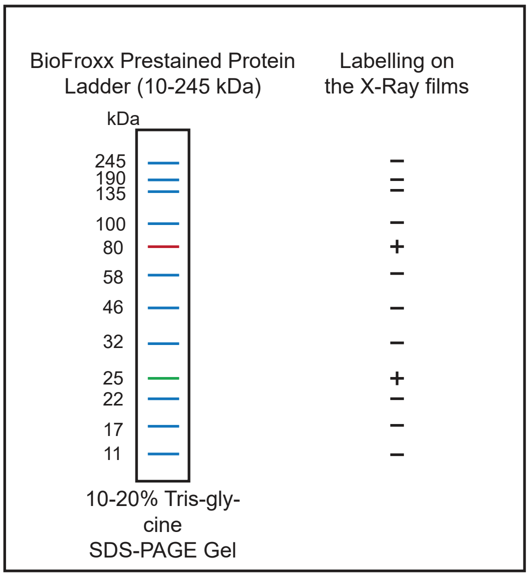

I do not have it.
Kori? 6713
312-392

Whole gel 12 (PD)
IB: GFP

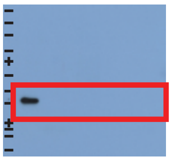

Whole gel 13 (PD)
IB: GFP

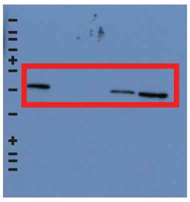

Whole gel 14 (PD)
IB: GFP

I do not have it.
Kori?
6712 850-893

Whole gel 15 (PD)
IB: GFP

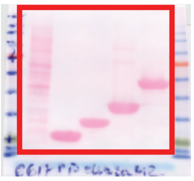

Whole gel 4 (PD)
Ponceau staining
representative image

Order of gels:

1. HeLa, mN4BP1(1-893), GST PD, IB:N4BP1
2. HeLa, mN4BP1(1-849), GST PD, IB:FLAG(M2)
3. HeLa, mN4BP1(1-771), GST PD, IB:FLAG(M2)
4. HeLa, mN4BP1(144-893), GST PD, IB:FLAG(M2)
5. HeLa, mN4BP1(343-893), GST PD, IB:N4BP1
6. HeLa, mN4BP1(393-893), GST PD, IB:FLAG(M2)
7. HeLa, mN4BP1(393-849), GST PD, IB:FLAG(M2)
8. HeLa, mN4BP1(393-771), GST PD, IB:FLAG(M2)
9. HeLa, mN4BP1(1-392), GST PD, IB:FLAG(M2)
10. HeLa, mN4BP1(1-143), GST PD, IB:FLAG(M2)
11. HeLa, mN4BP1(144-392), GST PD, IB:FLAG(M2)
12. HeLa, mN4BP1(312-392), GST PD, IB:GFP
13. HeLa, mN4BP1(312-342), GST PD, IB:GFP
14. HeLa, hN4BP1(703-896), GST PD, IB:GFP
15. HeLa, mN4BP1(850-893), GST PD, IB:GFP
16. Representative Ponceau staining prior to immunobloting

Order on each gel (left to right):

1. Input
2. GST alone
3. GST-Ub
4. GST-diUb
5. GST-tetraUb)

SUPPLEMENTARY FIGURE 2A - SOURCE DATA

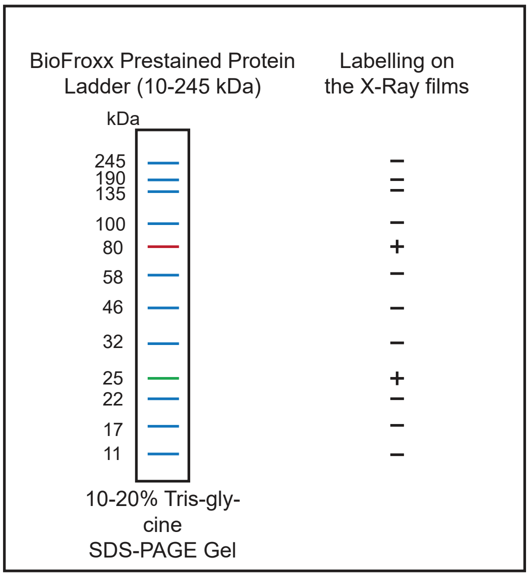

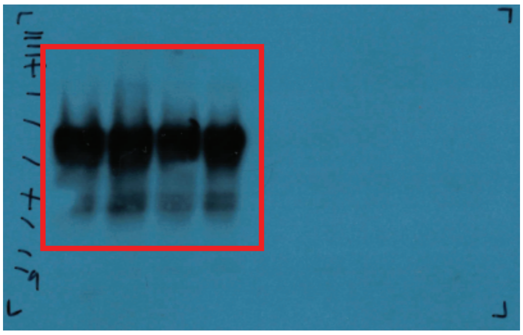

Whole gel 1 (IP N4BP1)
IB: Linear Ub

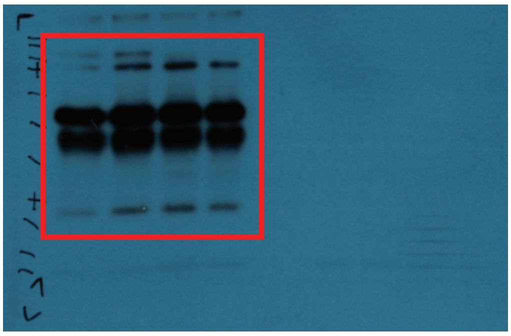

Whole gel 1 (IP N4BP1)
IB: N4BP1 (after stripping)

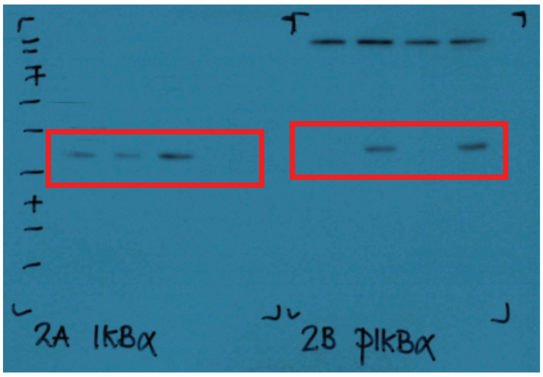

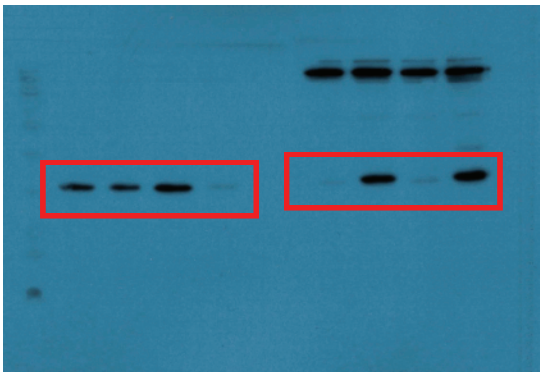

Whole gel 2 (TCL)
Left: IkappaBalpha
Right: phosphosIkappaBalpha

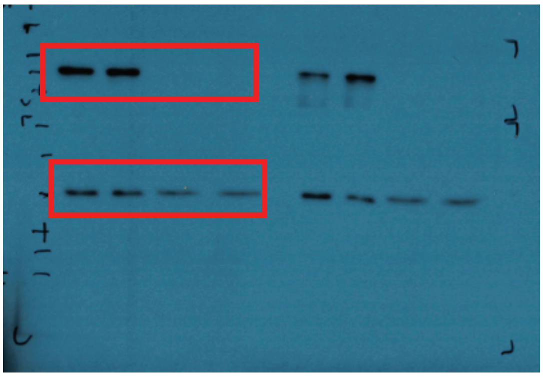

Whole gel 2 (TCL)
Top: N4BP1 (after stripping)
Bottom: GAPDH (after stripping)

Order on each gel (left to right):

1. MEF N4BP1$^{+/+}$ IP N4BP1, TNFalpha [20ng/ml; 0min]
2. MEF N4BP1$^{+/+}$ IP N4BP1, TNFalpha [20ng/ml; 10min]
3. MEF N4BP1$^{-/-}$ IP N4BP1, TNFalpha [20ng/ml; 0min]
4. MEF N4BP1$^{-/-}$ IP N4BP1, TNFalpha [20ng/ml; 10min]

SUPPLEMENTARY FIGURE 2B - SOURCE DATA

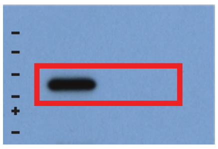

Whole gel 1 (TCL)
IB: N4BP1

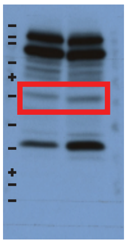

Whole gel 2 (TCL)
IB: TNFR1
Reprobed

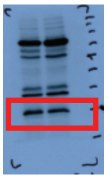

Whole gel 3 (TCL)
IB: TRADD
Reprobed after stripping

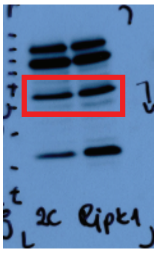

Whole gel 4 (TCL)
IB: RIP1

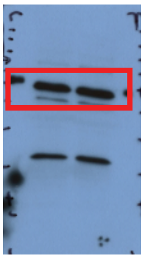

Whole gel 5 (TCL)
IB: HOIP

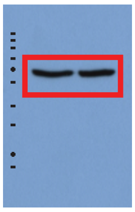

Whole gel 6 (TCL)
IB: IKKa

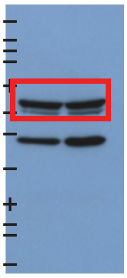

Whole gel 7 (TCL)
IB: IKKg

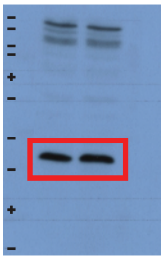

Whole gel 8 (TCL)
IB: IkBa

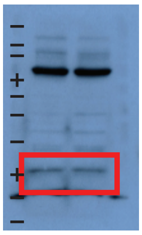

Whole gel 6 (TCL)
IB: FADD,
Reprobed after stripping

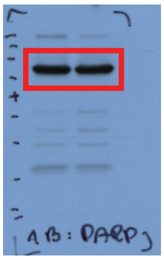

Whole gel 10 (TCL)
IB: PARP1

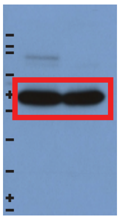

Whole gel 11 (TCL)
IB: p65

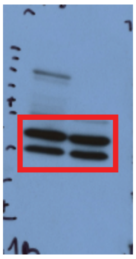

Whole gel 12 (TCL)
IB: JNK

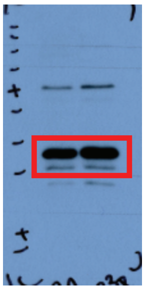

Whole gel 13 (TCL)
IB: p38

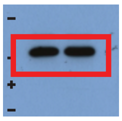

Whole gel 14 (TCL)
IB: GAPDH

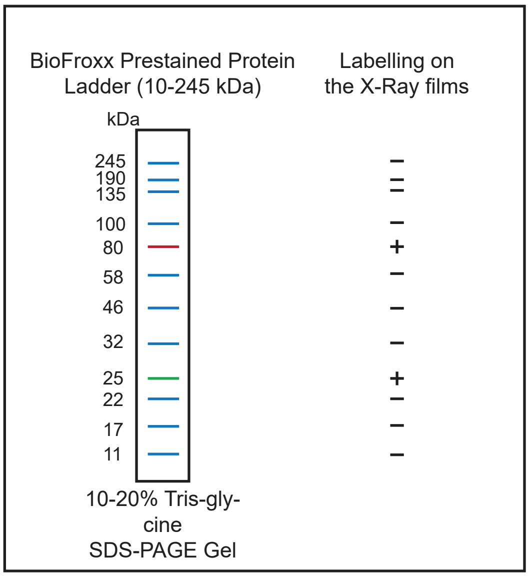

Order on each gel (left to right):

1. MEF N4BP1$^{+/+}$, TCL
2. MEF N4BP1 $^{-/-}$, TCL

SUPPLEMENTARY FIGURE 2G - SOURCE DATA

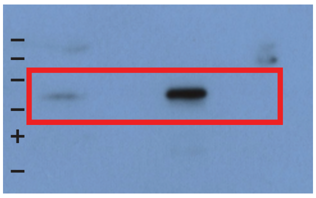

Gel 1 (Cell fractions): top part
IB: N4BP1

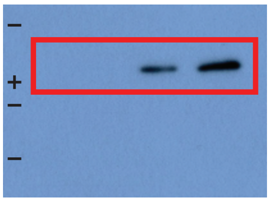

Gel 2 (Cell fractions): bottom part
IB: PCNA

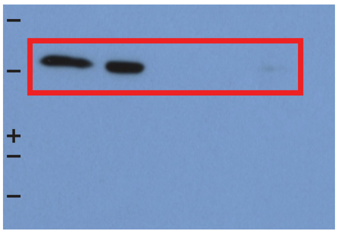

Gel 1 (Cell fractions): bottom part
IB: GAPDH

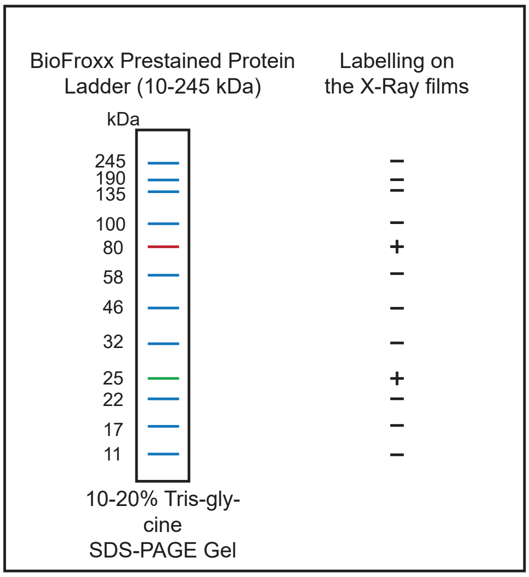

Order on each gel (left to right):

1. MEF N4BP1$^{+/+}$, cytoplasmic fraction
2. MEF N4BP1 $^{-/-}$, cytoplasmic fraction
3. MEF N4BP1$^{+/+}$, nuclear fraction
4. MEF N4BP1 $^{-/-}$, nuclear fraction

SUPPLEMENTARY FIGURE 2H - SOURCE DATA

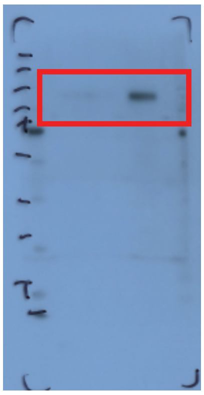

Gel 1 (Cell fractions): top part
IB: N4BP1

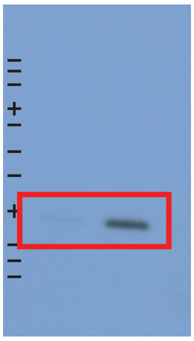

Gel 2 (Cell fractions): bottom part
IB: PCNA

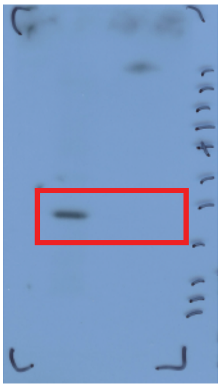

Gel 1 (Cell fractions): bottom part
IB: GAPDH

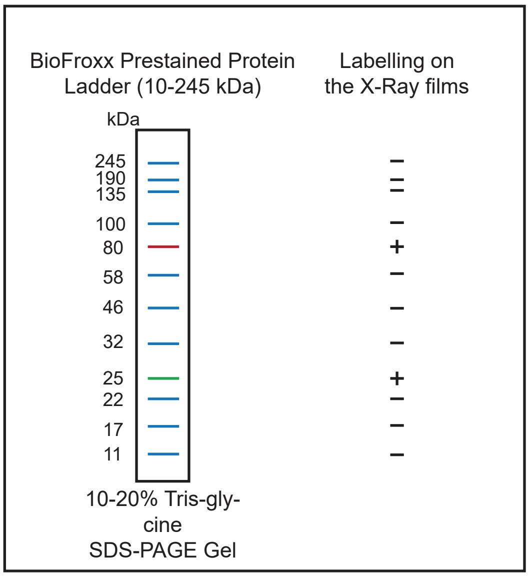

Order on each gel (left to right):

1. HEK293T, cytoplasmic fraction
2. HEK293T, nuclear fraction

SUPPLEMENTARY FIGURE 2I - SOURCE DATA

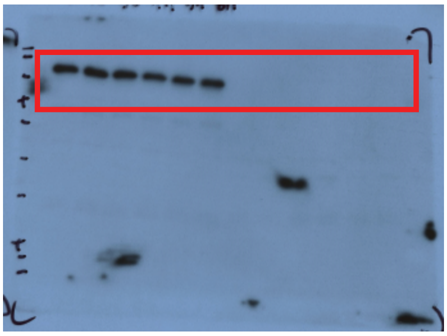

Whole gel 1 (TCL)
IB: N4BP1

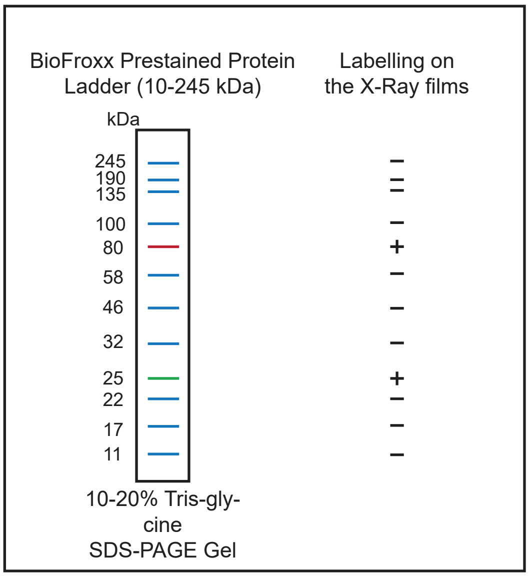

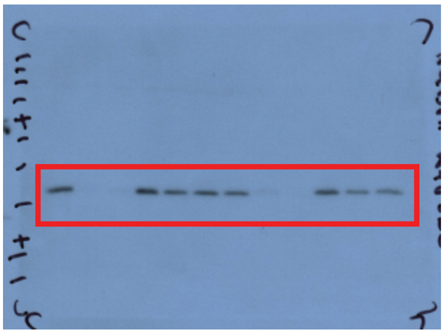

Whole gel 2 (TCL)
IB: IkappaBalpha (short exposure)

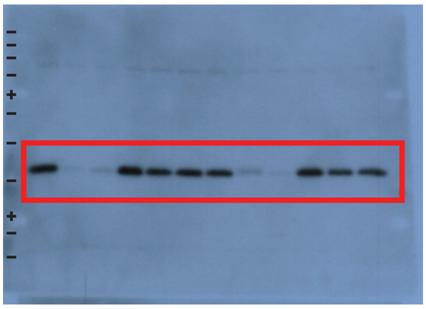

Whole gel 2 (TCL)
IB: IkappaBalpha (long exposure)

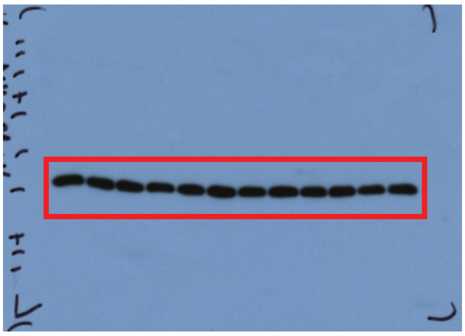

Whole gel 1 (TCL)
IB: GAPDH,
Reprobed after stripping

1. Order on each gel (left to right):
2. MEF N4BP1$^{+/+}$ TCL, IL-1beta [10ng/ml; 0min]
3. MEF N4BP1$^{+/+}$ TCL, IL-1beta [10ng/ml; 15min]
4. MEF N4BP1$^{+/+}$ TCL, IL-1beta [10ng/ml; 30min]
5. MEF N4BP1$^{+/+}$ TCL, IL-1beta [10ng/ml; 60min]
6. MEF N4BP1$^{+/+}$ TCL, IL-1beta [10ng/ml; 240min]
7. MEF N4BP1$^{+/+}$ TCL, IL-1beta [10ng/ml; 480min]
8. MEF N4BP1$^{-/-}$ TCL, IL-1beta [10ng/ml; 0min]
9. MEF N4BP1$^{-/-}$ TCL, IL-1beta [10ng/ml; 15min]
10. MEF N4BP1$^{-/-}$ TCL, IL-1beta [10ng/ml; 30min]
11. MEF N4BP1$^{-/-}$ TCL, IL-1beta [10ng/ml; 60min]
12. MEF N4BP1$^{-/-}$ TCL, IL-1beta [10ng/ml; 240min]
13. MEF N4BP1$^{-/-}$ TCL, IL-1beta [10ng/ml; 480min]

SUPPLEMENTARY FIGURE 2J - SOURCE DATA

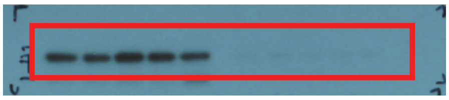

Gel 1 (top) (TCL)
IB: N4BP1
Samples 1-10

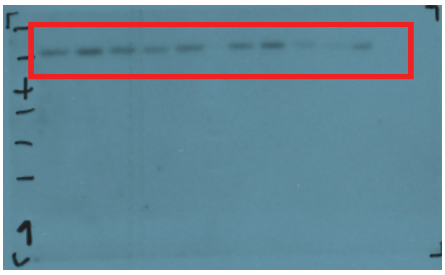

Gel 1 (bottom) (TCL)
IB: IkappaBalpha
Samples 1-10

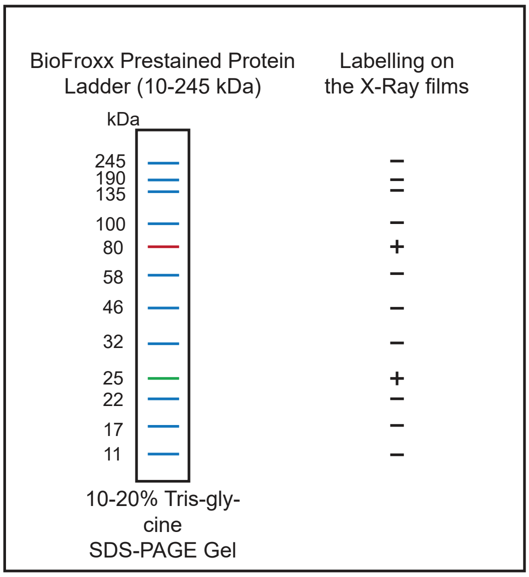

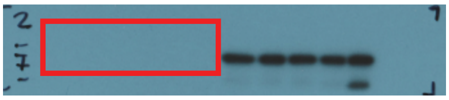

Gel 2 (top) (TCL)
IB: N4BP1
Samples 11-15

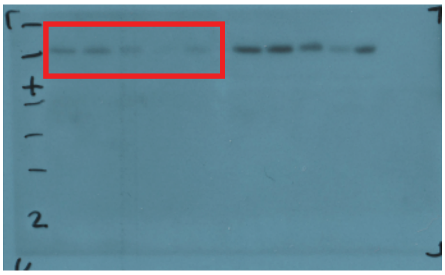

Gel 2 (bottom) (TCL)
IB: IkappaBalpha
Samples 11-15

Order on each gel (left to right):

1. MEF N4BP1$^{+/+}$ TCL, TNFalpha [20ng/ml; 0min]
2. MEF N4BP1$^{+/+}$ TCL, TNFalpha [20ng/ml; 5min]
3. MEF N4BP1$^{+/+}$ TCL, TNFalpha [20ng/ml; 15min]
4. MEF N4BP1$^{+/+}$ TCL, TNFalpha [20ng/ml; 30min]
5. MEF N4BP1$^{+/+}$ TCL, TNFalpha [20ng/ml; 60min]
6. MEF N4BP1$^{CRISPR-KO}$ TCL, TNFalpha [20ng/ml; 0min]
7. MEF N4BP1$^{CRISPR-KO}$ TCL, TNFalpha [20ng/ml; 5min]
8. MEF N4BP1$^{CRISPR-KO}$ TCL, TNFalpha [20ng/ml; 15min]
9. MEF N4BP1$^{CRISPR-KO}$ TCL, TNFalpha [20ng/ml; 30min]
10. MEF N4BP1$^{CRISPR-KO}$ TCL, TNFalpha [20ng/ml; 60min]
11. MEF N4BP1$^{-/-}$ TCL, TNFalpha [20ng/ml; 0min]
12. MEF N4BP1$^{-/-}$ TCL, TNFalpha [20ng/ml; 5min]
13. MEF N4BP1$^{-/-}$ TCL, TNFalpha [20ng/ml; 15min]
14. MEF N4BP1$^{-/-}$ TCL, TNFalpha [20ng/ml; 30min]
15. MEF N4BP1$^{-/-}$ TCL, TNFalpha [20ng/ml; 60min]

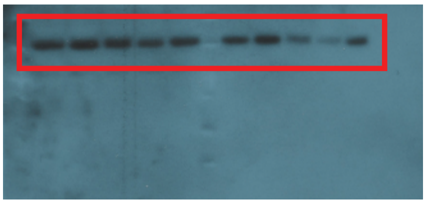

Gel 1 (bottom) (TCL)
IB: IkappaBalpha
Samples 1-10

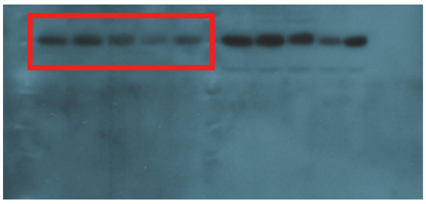

Gel 2 (bottom) (TCL)
IB: IkappaBalpha
Samples 11-15

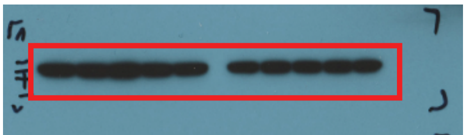

Gel 1 (top) (TCL)
IB: Vinculin (after stripping)
Samples 1-10

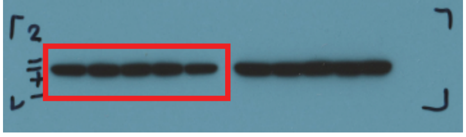

Gel 2 (top) (TCL)
IB: Vinculin (after stripping)
Samples 11-15

SUPPLEMENTARY FIGURE 3A - SOURCE DATA

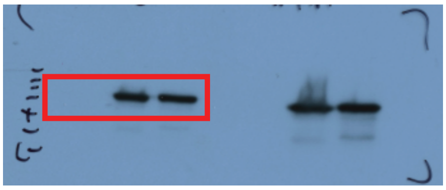

Gel 1 (TCL): top part
IB: N4BP1

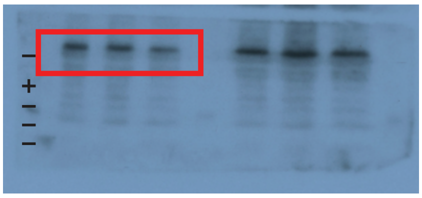

Gel 1 (TCL): bottom part
IB: GAPDH

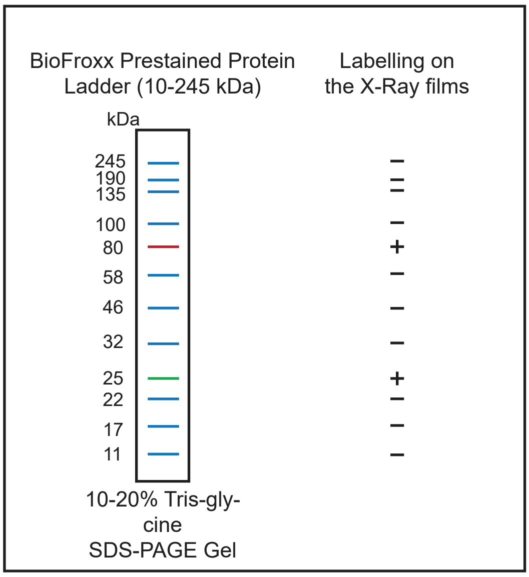

Order on each gel (left to right):

1. MEF N4BP1 $^{-/-}$, reconstituted with empty vector, TCL
2. MEF N4BP1 $^{-/-}$, reconstituted with HA-N4BP1(1-893), TCL
3. MEF N4BP1 $^{-/-}$, reconstituted with HA-N4BP1(1-893, F863G, P864A), TCL

SUPPLEMENTARY FIGURE 3B - SOURCE DATA

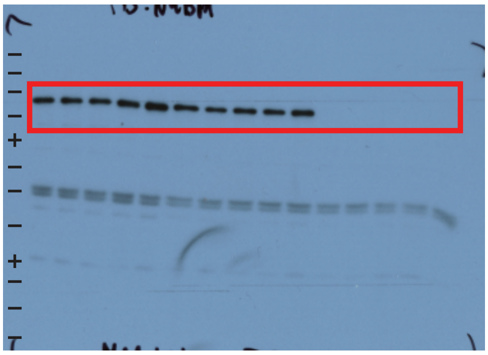

Whole gel 1 (TCL)
IB: N4BP1

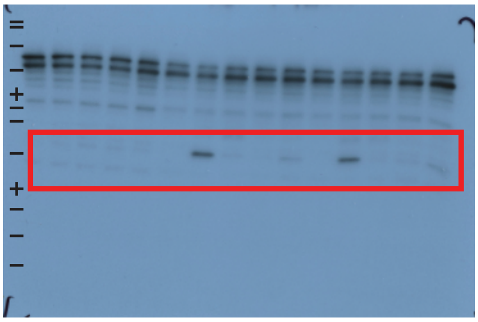

Whole gel 2 (TCL)
IB: P-IkappaBalpha

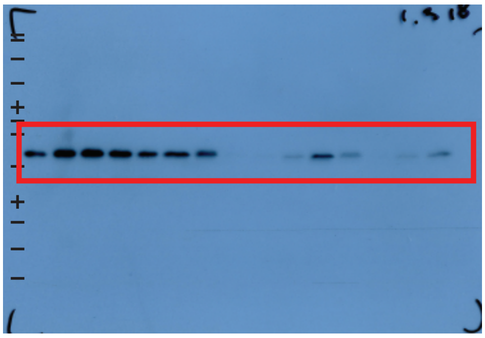

Whole gel 3 (TCL)
IB: IkappaBalpha

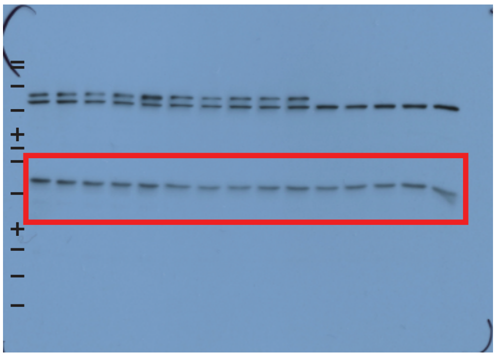

Whole gel 4 (TCL)
IB: GAPDH

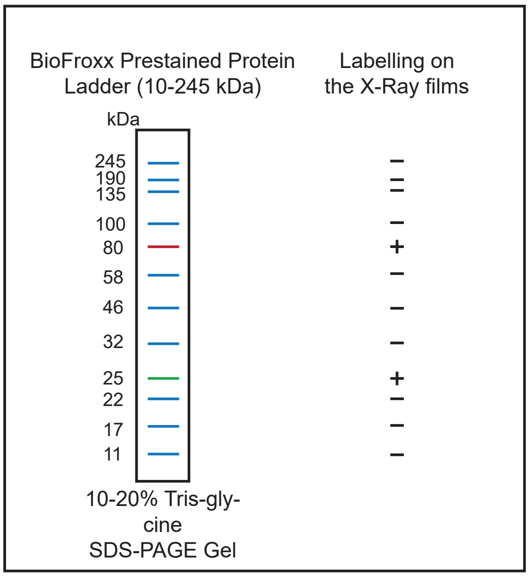

Order on each gel (left to right):

1. MEF N4BP1 $^{-/-}$, reconstituted with HA-N4BP1(1-893), TCL, TNFalpha [20ng/ml; 0min]
2. MEF N4BP1 $^{-/-}$, reconstituted with HA-N4BP1(1-893), TCL, TNFalpha [20ng/ml; 5min]
3. MEF N4BP1 $^{-/-}$, reconstituted with HA-N4BP1(1-893), TCL, TNFalpha [20ng/ml; 15min]
4. MEF N4BP1 $^{-/-}$, reconstituted with HA-N4BP1(1-893), TCL, TNFalpha [20ng/ml; 30min]
5. MEF N4BP1 $^{-/-}$, reconstituted with HA-N4BP1(1-893), TCL, TNFalpha [20ng/ml; 60min]
6. MEF N4BP1 $^{-/-}$, reconstituted with HA-N4BP1(1-893, F863G, P864A), TCL, TNFalpha [20ng/ml; 0min]
7. MEF N4BP1 $^{-/-}$, reconstituted with HA-N4BP1(1-893, F863G, P864A), TCL, TNFalpha [20ng/ml; 5min]
8. MEF N4BP1 $^{-/-}$, reconstituted with HA-N4BP1(1-893, F863G, P864A), TCL, TNFalpha [20ng/ml; 15min]
9. MEF N4BP1 $^{-/-}$, reconstituted with HA-N4BP1(1-893, F863G, P864A), TCL, TNFalpha [20ng/ml; 30min]
10. MEF N4BP1 $^{-/-}$, reconstituted with HA-N4BP1(1-893, F863G, P864A), TCL, TNFalpha [20ng/ml; 60min]
11. MEF N4BP1 $^{-/-}$, reconstituted with empty vector, TCL, TNFalpha [20ng/ml; 0min]
12. MEF N4BP1 $^{-/-}$, reconstituted with empty vector, TCL, TNFalpha [20ng/ml; 5min]
13. MEF N4BP1 $^{-/-}$, reconstituted with empty vector, TCL, TNFalpha [20ng/ml; 15min]
14. MEF N4BP1 $^{-/-}$, reconstituted with empty vector, TCL, TNFalpha [20ng/ml; 30min]
15. MEF N4BP1 $^{-/-}$, reconstituted with empty vector, TCL, TNFalpha [20ng/ml; 60min]

SUPPLEMENTARY FIGURE 3C - SOURCE DATA

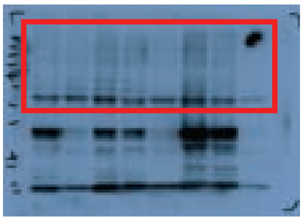

Whole gel 1 (IP)
IB: NEMO

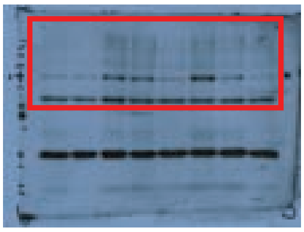

Whole gel 2 (IP)
IB: RIP1

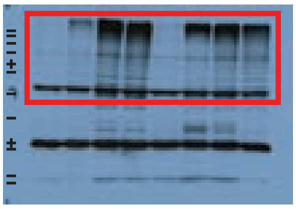

Whole gel 2 (IP)
IB: LinUb
Reprobed after stripping

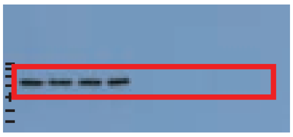

Gel 3 (TCL): top part
IB: N4BP1

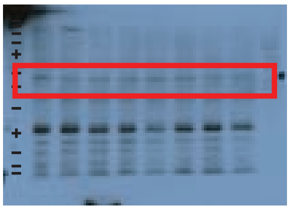

Whole gel 4 (TCL)
IB: NEMO

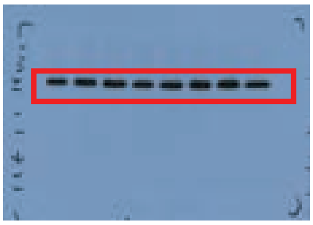

Whole gel 5 (TCL)
IB: RIP1

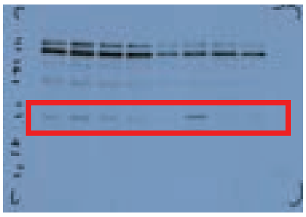

Whole gel 6 (TCL)
IB: Phospho-IkappaBalpha

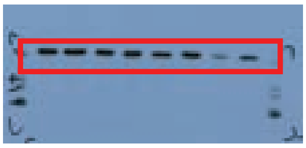

Gel 3 (TCL): bottom part
IB: IkappaBalpha

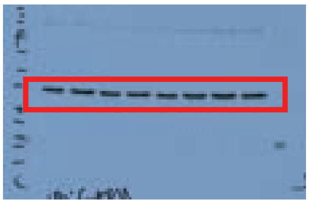

Whole gel 7 (TCL)
IB: GAPDH

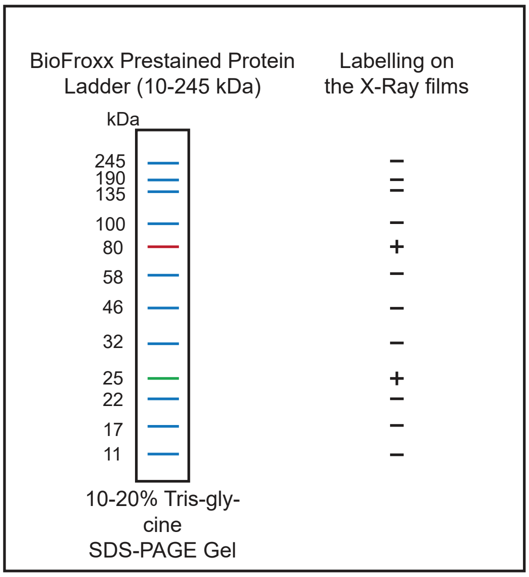

Order on each gel (left to right):

1. MEF N4BP1$^{+/+}$, TNFalpha [20ng/ml; 0min]
2. MEF N4BP1$^{+/+}$, TNFalpha [20ng/ml; 5min]
3. MEF N4BP1$^{+/+}$, TNFalpha [20ng/ml; 15min]
4. MEF N4BP1$^{+/+}$, TNFalpha [20ng/ml; 45min]
5. MEF N4BP1 $^{-/-}$, TNFalpha [20ng/ml; 0min]
6. MEF N4BP1 $^{-/-}$, TNFalpha [20ng/ml; 5min]
7. MEF N4BP1 $^{-/-}$, TNFalpha [20ng/ml; 15min]
8. MEF N4BP1 $^{-/-}$, TNFalpha [20ng/ml; 45min]

SUPPLEMENTARY FIGURE 4A - SOURCE DATA

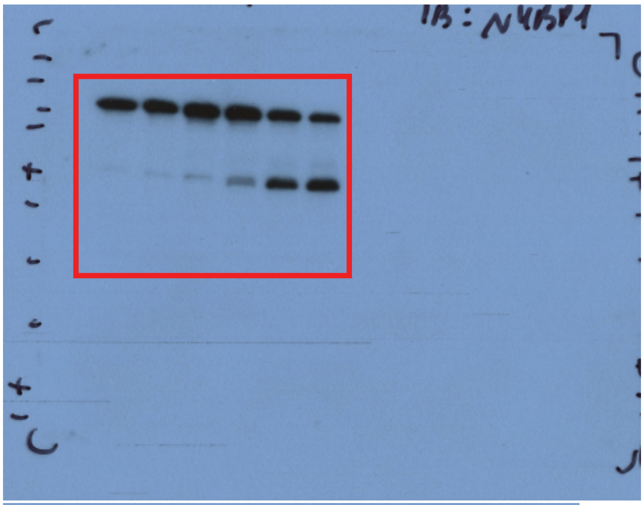

Whole gel 1 (TCL)
IB: N4BP1

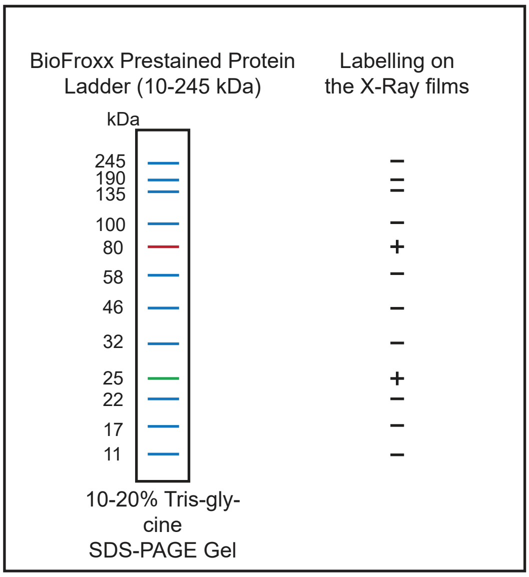

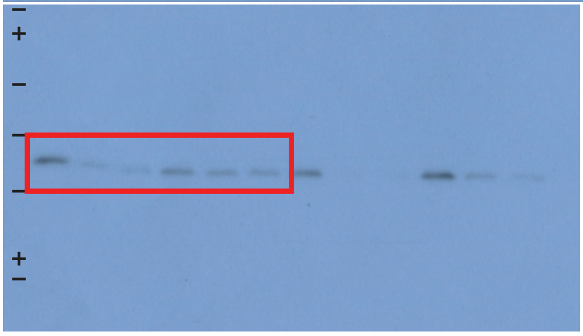

Whole gel 2 (TCL)
IB: IkappaBalpha (short)

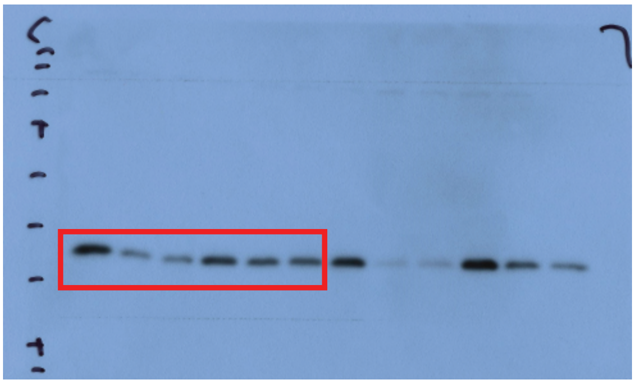

Whole gel 2 (TCL)
IB: IkappaBalpha (long)

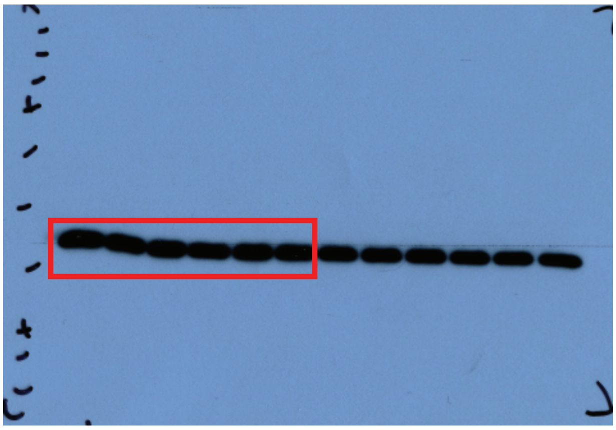

Whole gel 1 (TCL)
IB: GAPDH
Reprobed after stripping

1. Order on each gel (left to right):
2. MEF N4BP1$^{+/+}$ TCL, TNFalpha [20ng/ml; 0min]
3. MEF N4BP1$^{+/+}$ TCL, TNFalpha [20ng/ml; 15min]
4. MEF N4BP1$^{+/+}$ TCL, TNFalpha [20ng/ml; 30min]
5. MEF N4BP1$^{+/+}$ TCL, TNFalpha [20ng/ml; 60min]
6. MEF N4BP1$^{+/+}$ TCL, TNFalpha [20ng/ml; 240min]
7. MEF N4BP1$^{+/+}$ TCL, TNFalpha [20ng/ml; 480min]

SUPPLEMENTARY FIGURE 4B - SOURCE DATA

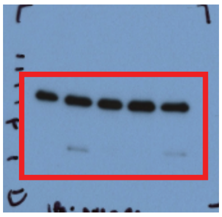

Gel 1 (TCL): top part
IB: N4BP1

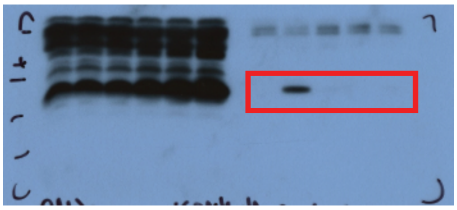

Gel 2 (TCL): bottom part
IB: Cleaved Caspase 3

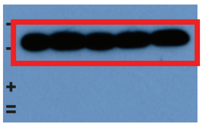

Gel 1 (TCL): bottom part
IB: GAPDH

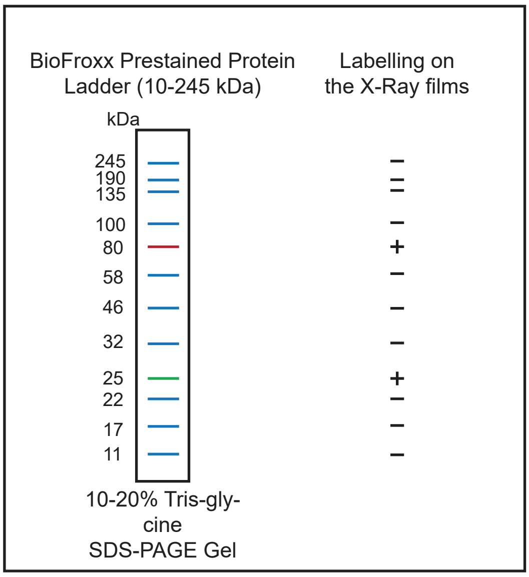

Order on each gel (left to right):

1. MEF N4BP1$^{+/+}$ TCL, TNFalpha [20ng/ml; 0h], z-VAD-fmk [20uM, 0h], z-IETD-fmk [20uM, 0h], ac-DEVD-cmk [20uM, 0h]
2. MEF N4BP1$^{+/+}$ TCL, TNFalpha [20ng/ml; 3h], z-VAD-fmk [20uM, 0h], z-IETD-fmk [20uM, 0h], ac-DEVD-cmk [20uM, 0h]
3. MEF N4BP1$^{+/+}$ TCL, TNFalpha [20ng/ml; 3h], z-VAD-fmk [20uM, 3h], z-IETD-fmk [20uM, 0h], ac-DEVD-cmk [20uM, 0h]
4. MEF N4BP1$^{+/+}$ TCL, TNFalpha [20ng/ml; 3h], z-VAD-fmk [20uM, 0h], z-IETD-fmk [20uM, 3h], ac-DEVD-cmk [20uM, 0h]
5. MEF N4BP1$^{+/+}$ TCL, TNFalpha [20ng/ml; 3h], z-VAD-fmk [20uM, 0h], z-IETD-fmk [20uM, 0h], ac-DEVD-cmk [20uM, 3h]

SUPPLEMENTARY FIGURE 4C - SOURCE DATA

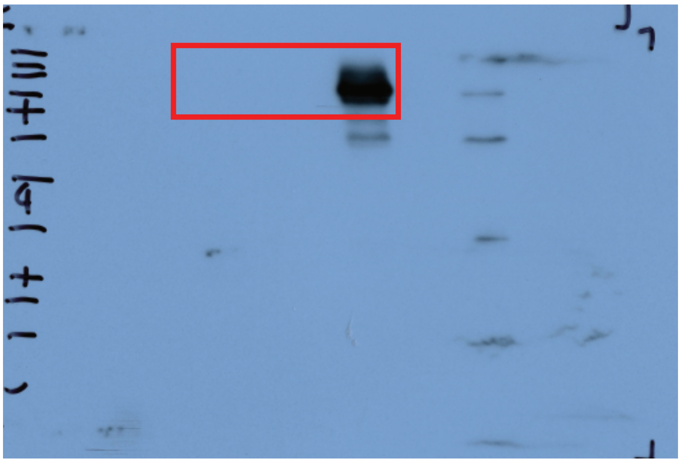

Whole gel 1 (IP)
IB: N4BP1

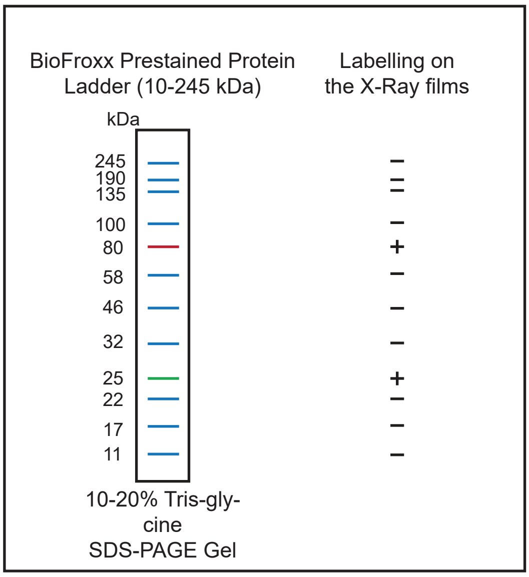

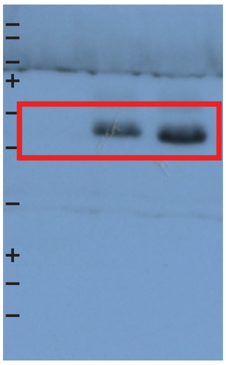

Whole gel 1 (IP)
IB: HA
Reprobed after stripping

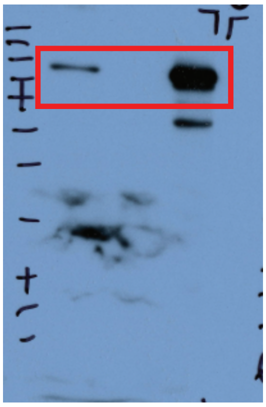

Whole gel 2 (TCL)
IB: N4BP1

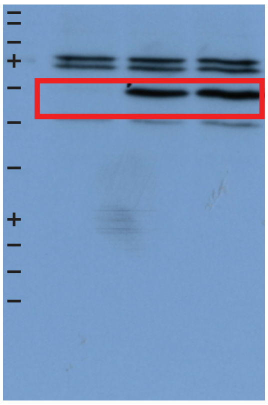

Order on each gel (left to right):

1. HEK293T, overexpressed mN4BP1 (1-893), empty vector
2. HEK293T, overexpressed HA-CASPASE 8 C360S, empty vector
3. HEK293T, overexpressed mN4BP1 (1-893), HA-CASPASE 8 C360S

SUPPLEMENTARY FIGURE 4D - SOURCE DATA

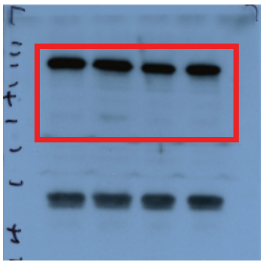

Whole gel 1 (TCL)
IB: N4BP1

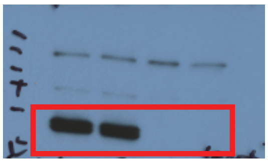

Gel 2 (TCL): top part
IB: Caspase 8

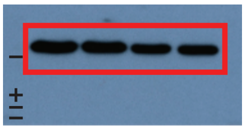

Gel 2 (TCL): bottom part
IB: GAPDH

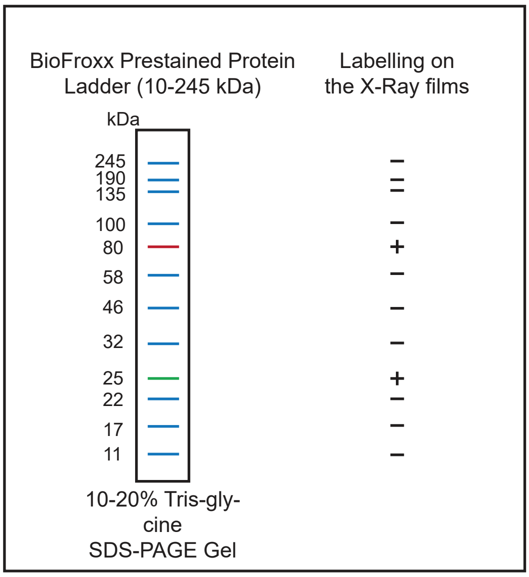

Order on each gel (left to right):

1. JURKAT Caspase 8$^{+/+}$, TNFalpha [100ng/ml, 0h]
2. JURKAT Caspase 8$^{+/+}$, TNFalpha [100ng/ml, 3h]
3. JURKAT Caspase 8$^{-/-}$, TNFalpha [100ng/ml, 0h]
4. JURKAT Caspase 8$^{-/-}$, TNFalpha [100ng/ml, 3h]

SUPPLEMENTARY FIGURE 4E - SOURCE DATA

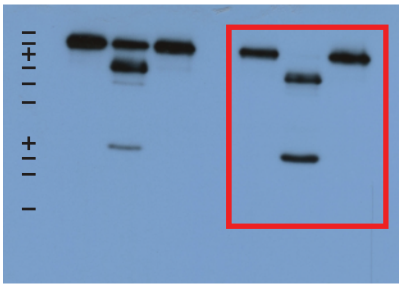

Whole gel 1 (in vitro cleavage assay)
IB: N4BP1

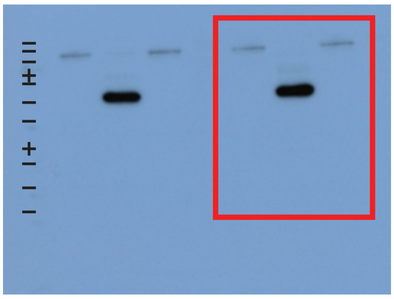

Whole gel 1 (in vitro cleavage assay)
IB: FLAG (M2)
Reprobed after stripping

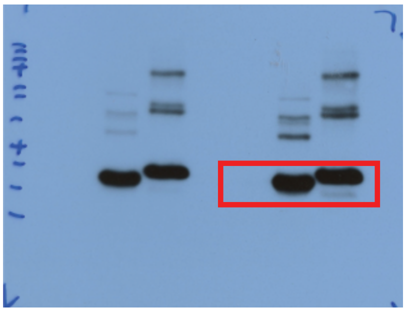

Whole gel 1 (in vitro cleavage assay)
IB: CASPASE 8
Reprobed after stripping (2)

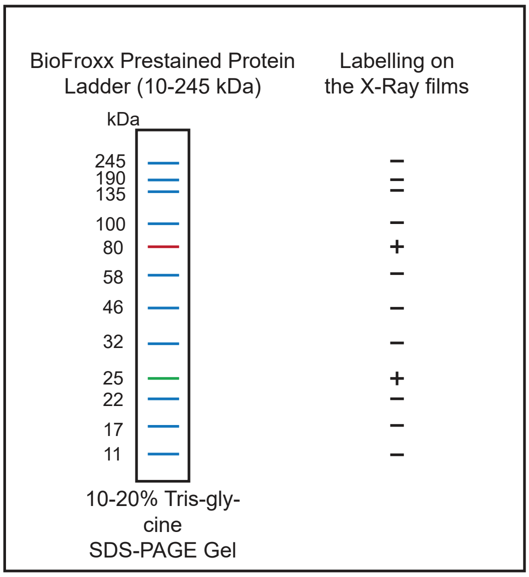

Order on each gel (left to right):

1. HEK293T, overexpressed mN4BP1 (1-893)-FLAG, FLAG IP
2. HEK293T, overexpressed mN4BP1 (1-893)-FLAG, FLAG IP followed by in vitro cleavage assay with CASPASE 8 [0.03 ug/ul, 3h]
3. HEK293T, overexpressed mN4BP1 (1-893)-FLAG, FLAG IP followed by in vitro cleavage assay with CASPASE 8 C360S [0.03 ug/ul, 3h]

SUPPLEMENTARY FIGURE 4F - SOURCE DATA

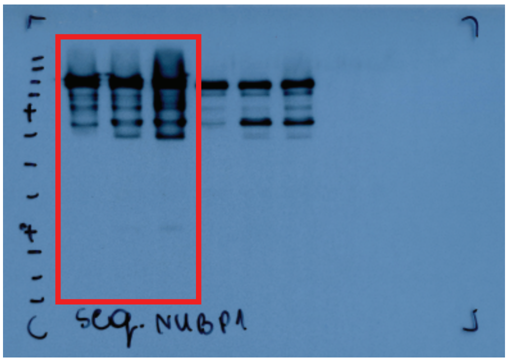

Whole gel 1 (TCL)
IB: N4BP1 (short)

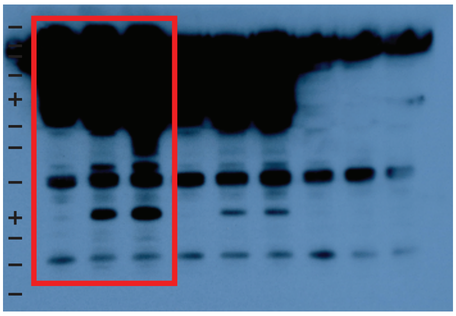

Whole gel 1 (TCL)
IB: N4BP1 (long)

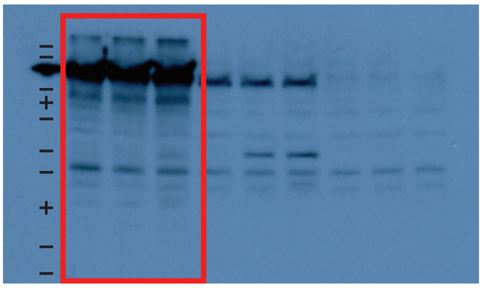

Whole gel 2 (TCL)
IB: FLAG (M2) (short)

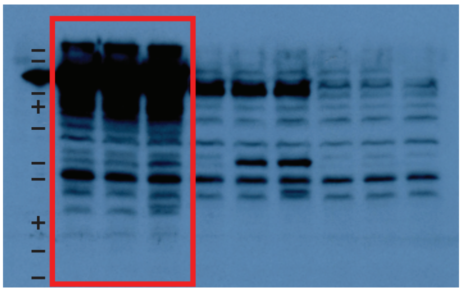

Whole gel 2 (TCL)
IB: FLAG (M2) (long)

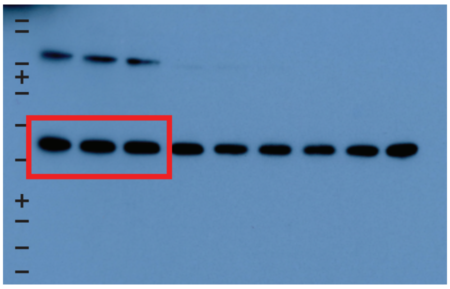

Whole gel 1 (TCL)
IB: GAPDH
Reprobed after stripping

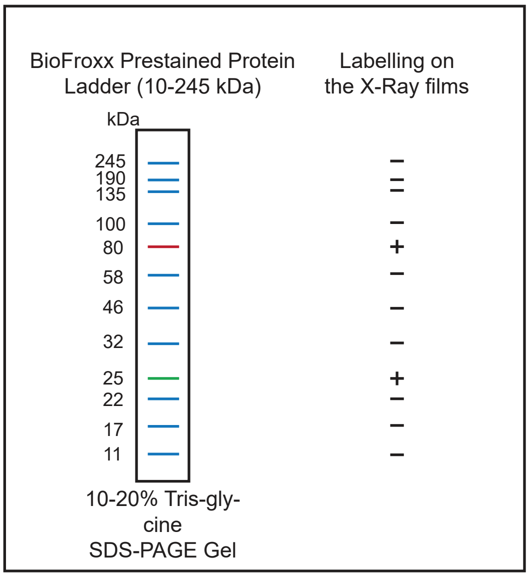

Order on each gel (left to right):

1. HEK293T, overexpressed FLAG-mN4BP1 (1-893), TNFalpha [20ng/ml, 0h], MG132 [10uM, 0h]
2. HEK293T, overexpressed FLAG-mN4BP1 (1-893), TNFalpha [20ng/ml, 6h], MG132 [10uM, 0h]
3. HEK293T, overexpressed FLAG-mN4BP1 (1-893), TNFalpha [20ng/ml, 6h], MG132 [10uM, 6h]

SUPPLEMENTARY FIGURE 4G - SOURCE DATA

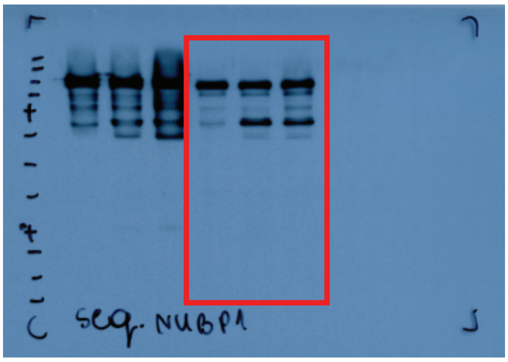

Whole gel 1 (TCL)
IB: N4BP1 (short)

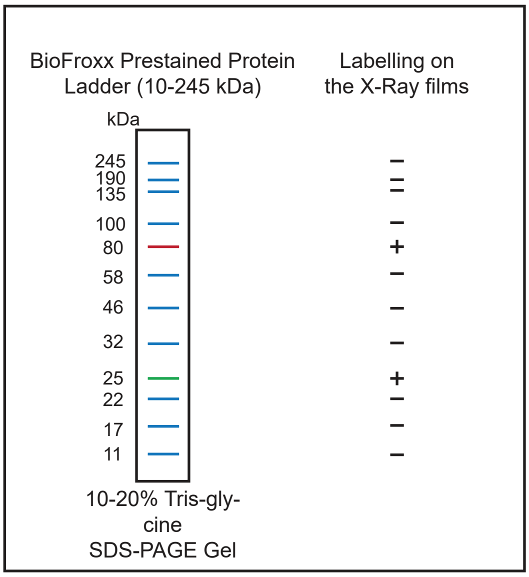

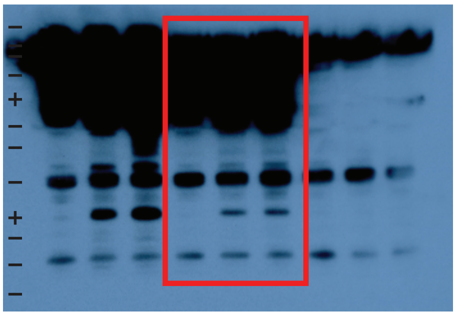

Whole gel 1 (TCL)
IB: N4BP1 (long)

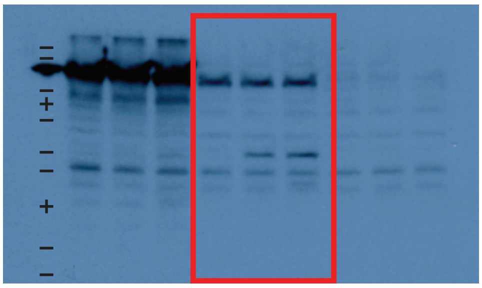

Whole gel 2 (TCL)
IB: FLAG (M2) (short)

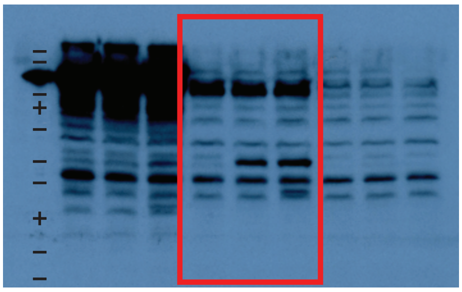

Whole gel 2 (TCL)
IB: FLAG (M2) (long)

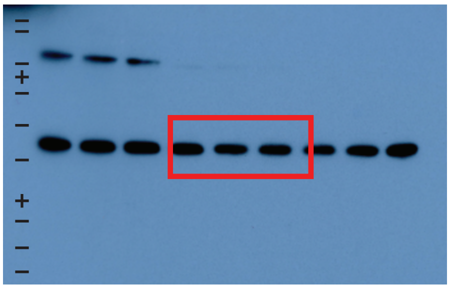

Whole gel 1 (TCL)
IB: GAPDH
Reprobed after stripping

Order on each gel (left to right):

1. HEK293T, overexpressed mN4BP1 (1-893)-FLAG, TNFalpha [20ng/ml, 0h], MG132 [10uM, 0h]
2. HEK293T, overexpressed mN4BP1 (1-893)-FLAG, TNFalpha [20ng/ml, 6h], MG132 [10uM, 0h]
3. HEK293T, overexpressed mN4BP1 (1-893)-FLAG, TNFalpha [20ng/ml, 6h], MG132 [10uM, 6h]

SUPPLEMENTARY FIGURE 5C - SOURCE DATA

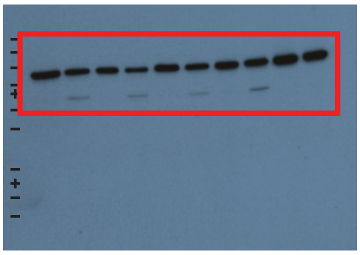

Whole gel 1 (TCL)
IB: N4BP1

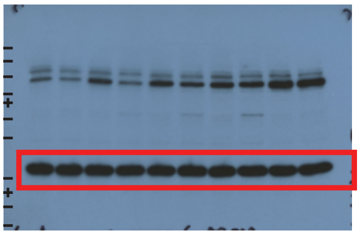

Whole gel 1 (TCL)
IB: GAPDH
Reprobed after stripping

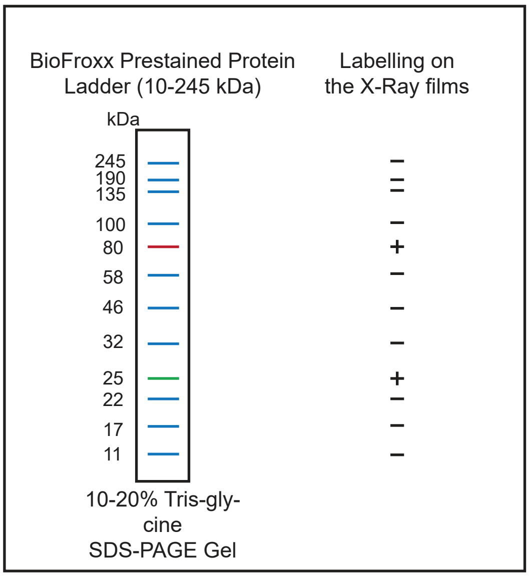

Order on each gel (left to right):

1. HEK293T, overexpressed mN4BP1 (1-893), TNFalpha [20ng/ml, 0h]
2. HEK293T, overexpressed mN4BP1 (1-893), TNFalpha [20ng/ml, 4h]
3. HEK293T, overexpressed mN4BP1 (1-893, D311A), TNFalpha [20ng/ml, 0h]
4. HEK293T, overexpressed mN4BP1 (1-893, D311A), TNFalpha [20ng/ml, 4h]
5. HEK293T, overexpressed mN4BP1 (1-893, D488A), TNFalpha [20ng/ml, 0h]
6. HEK293T, overexpressed mN4BP1 (1-893, D488A), TNFalpha [20ng/ml, 4h]
7. HEK293T, overexpressed mN4BP1 (1-893, D484/488A), TNFalpha [20ng/ml, 0h]
8. HEK293T, overexpressed mN4BP1 (1-893, D484/488A), TNFalpha [20ng/ml, 4h]
9. HEK293T, overexpressed mN4BP1 (1-893, D311/484/488A), TNFalpha [20ng/ml, 0h]
10. HEK293T, overexpressed mN4BP1 (1-893, D311/484/488A), TNFalpha [20ng/ml, 4h]

SUPPLEMENTARY FIGURE 5D - SOURCE DATA

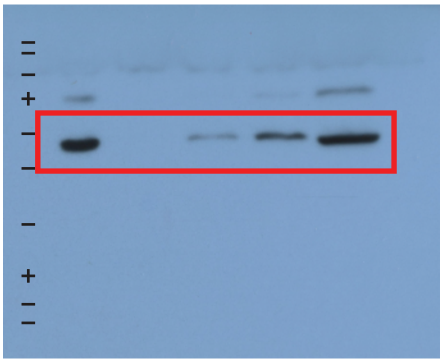

Whole gel 1 (PD)
IB: FLAG (M2)

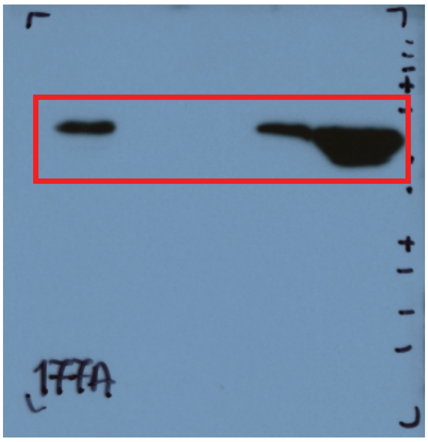

Whole gel 2 (PD)
IB: FLAG (M2)

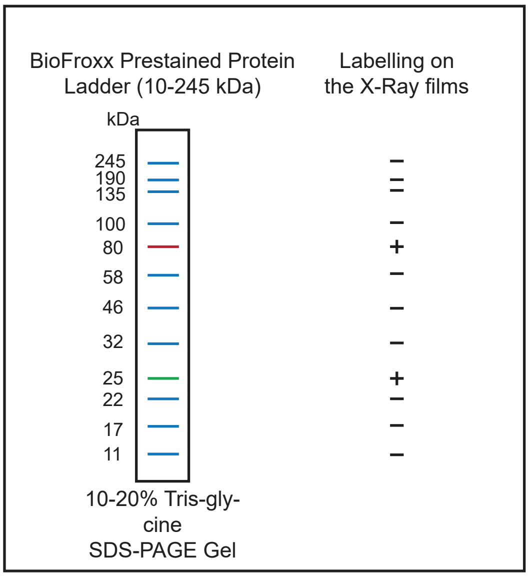

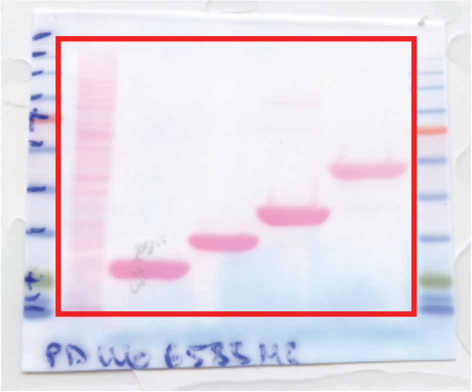

Whole gel 3 (PD)
Ponceau staining
representative image

Order of gels:
1. HEK293T, FLAG-mN4BP1(1-488), GST PD, IB:FLAG (M2)
2. HEK293T, FLAG-mN4BP1(489-893), GST PD, IB:FLAG (M2)
3. Representative Ponceau staining prior to immunobloting

Order on each gel (left to right):
1. Input
2. GST alone
3. GST-Ub
4. GST-diUb
5. GST-tetraUb)

SUPPLEMENTARY FIGURE 5E - SOURCE DATA

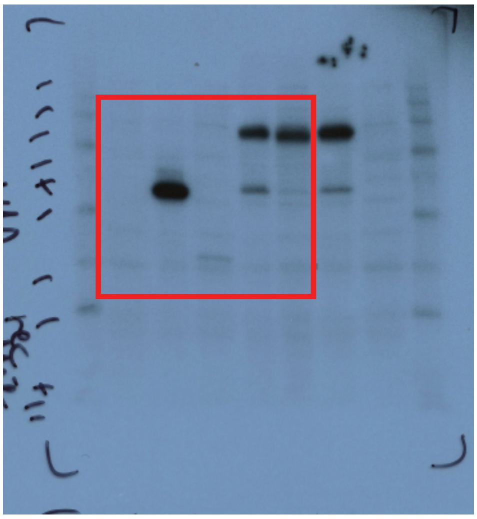

Whole gel 1 (TCL)
IB: HA

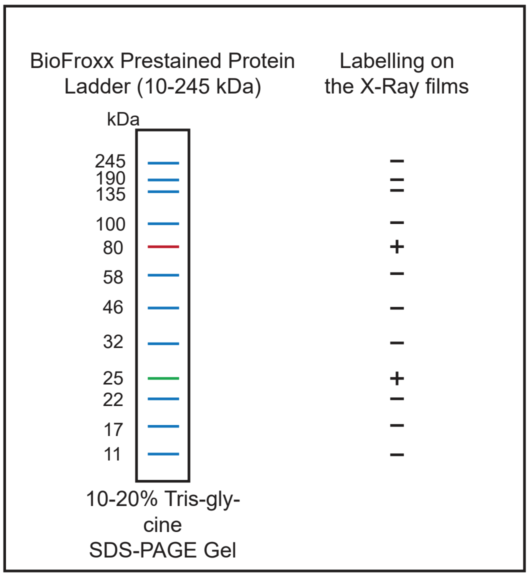

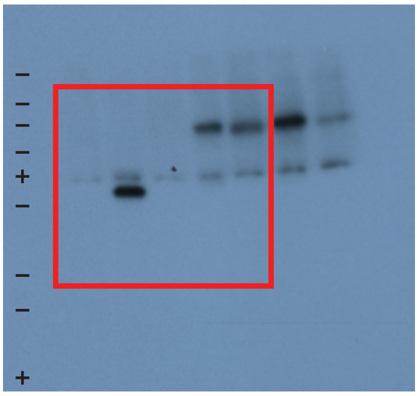

Whole gel 2 (TCL)
IB: N4BP1

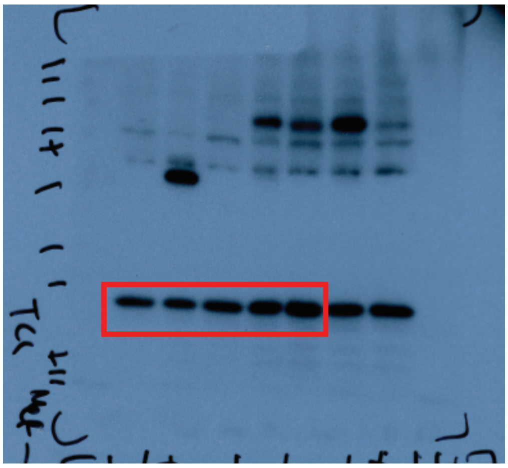

Whole gel 1 (TCL)
IB: GAPDH
Reprobed after stripping

Order on each gel (left to right):

1. MEF N4BP1 $^{-/-}$, reconstituted with empty vector, TCL
2. MEF N4BP1 $^{-/-}$, reconstituted with HA-N4BP1(1-488), TCL
3. MEF N4BP1 $^{-/-}$, reconstituted with HA-N4BP1(489-893), TCL
4. MEF N4BP1 $^{-/-}$, reconstituted with HA-N4BP1(1-893), TCL
5. MEF N4BP1 $^{-/-}$, reconstituted with HA-N4BP1(1-893, D311/484/488A), TCL

SUPPLEMENTARY FIGURE 5F - SOURCE DATA

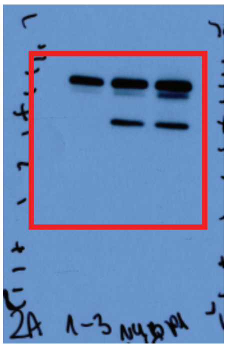

Gel 1 (TCL): left part
IB: N4BP1

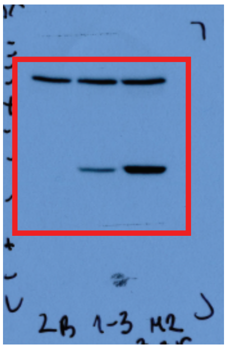

Gel 1 (TCL): right part
IB: FLAG (M2)

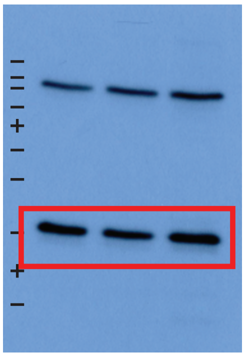

Gel 1 (TCL): right part
IB: GAPDH
Reprobed after stripping

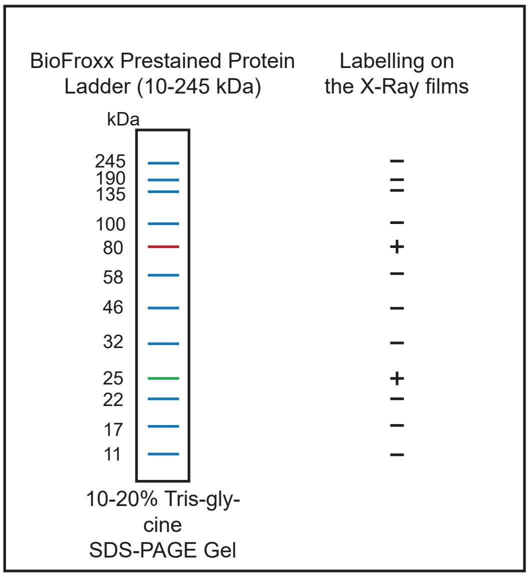

Order on each gel (left to right):

1. HEK293T, overexpressed mN4BP1 (1-893)-FLAG, TNFalpha [20ng/ml, 0h], MG132 [10uM, 0h]
2. HEK293T, overexpressed mN4BP1 (1-893)-FLAG, TNFalpha [20ng/ml, 6h], MG132 [10uM, 0h]
3. HEK293T, overexpressed mN4BP1 (1-893)-FLAG, TNFalpha [20ng/ml, 6h], MG132 [10uM, 6h]

FIGURE 7B - SOURCE DATA

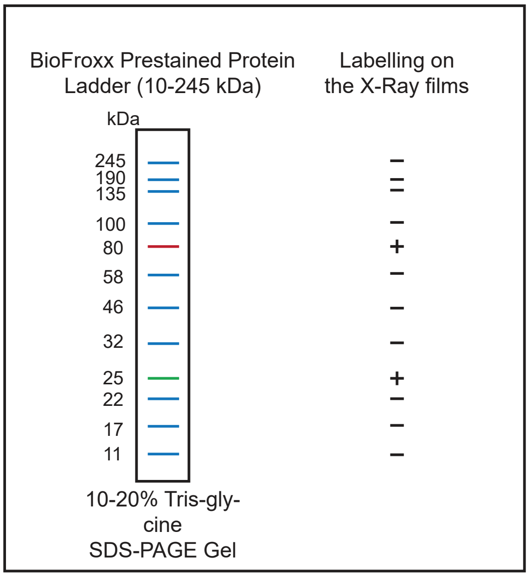

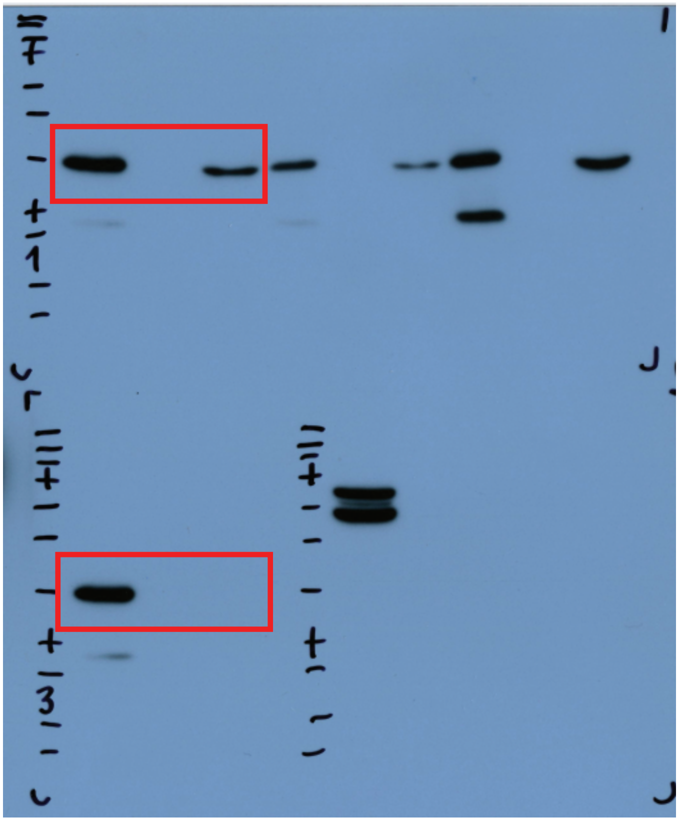

Whole gel 1

IB:GFP

Whole gel 2

IB:GFP

Order (marked area):
1. Input
2. GST alone
3. PD GST-diUb

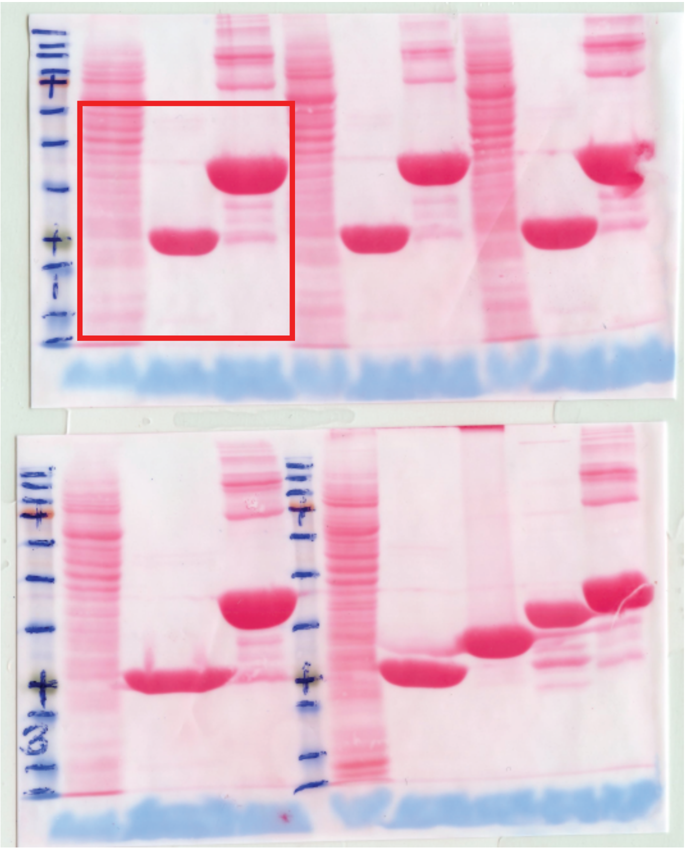

Whole gels

Ponceau staining
